# Supplementary figures and images for: Exploring the potential impact of the proposed UK TV and online food advertising regulations: a concept mapping study
Source: BMJ Open. 2022 Jun 16;12(6):e060302. doi: 10.1136/bmjopen-2021-060302 (PMC9207937; doi:10.1136/bmjopen-2021-060302)

Appendix 2: Maps 1-4 produced by each workshop

Workshop 1 Map

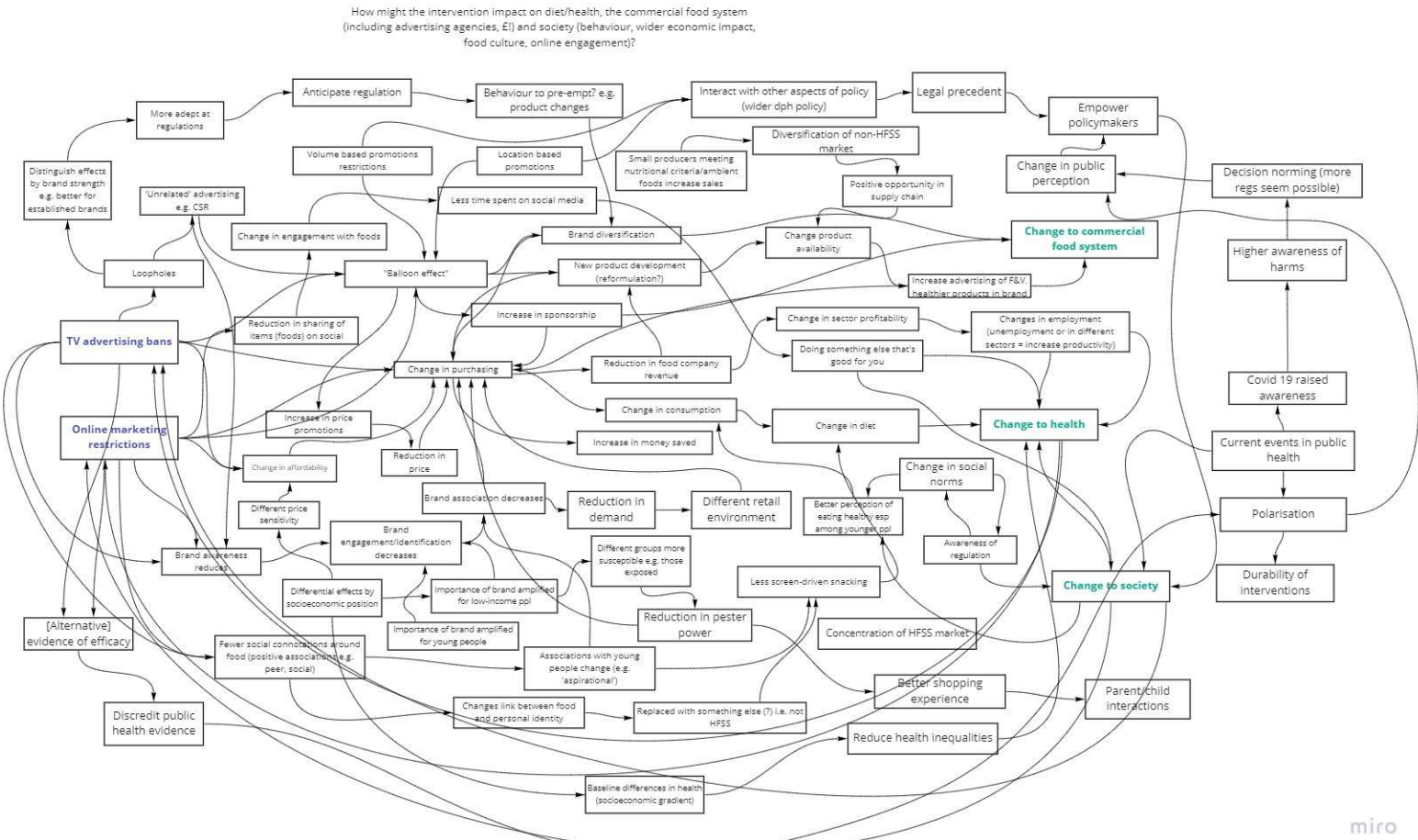

Workshop 2 Map

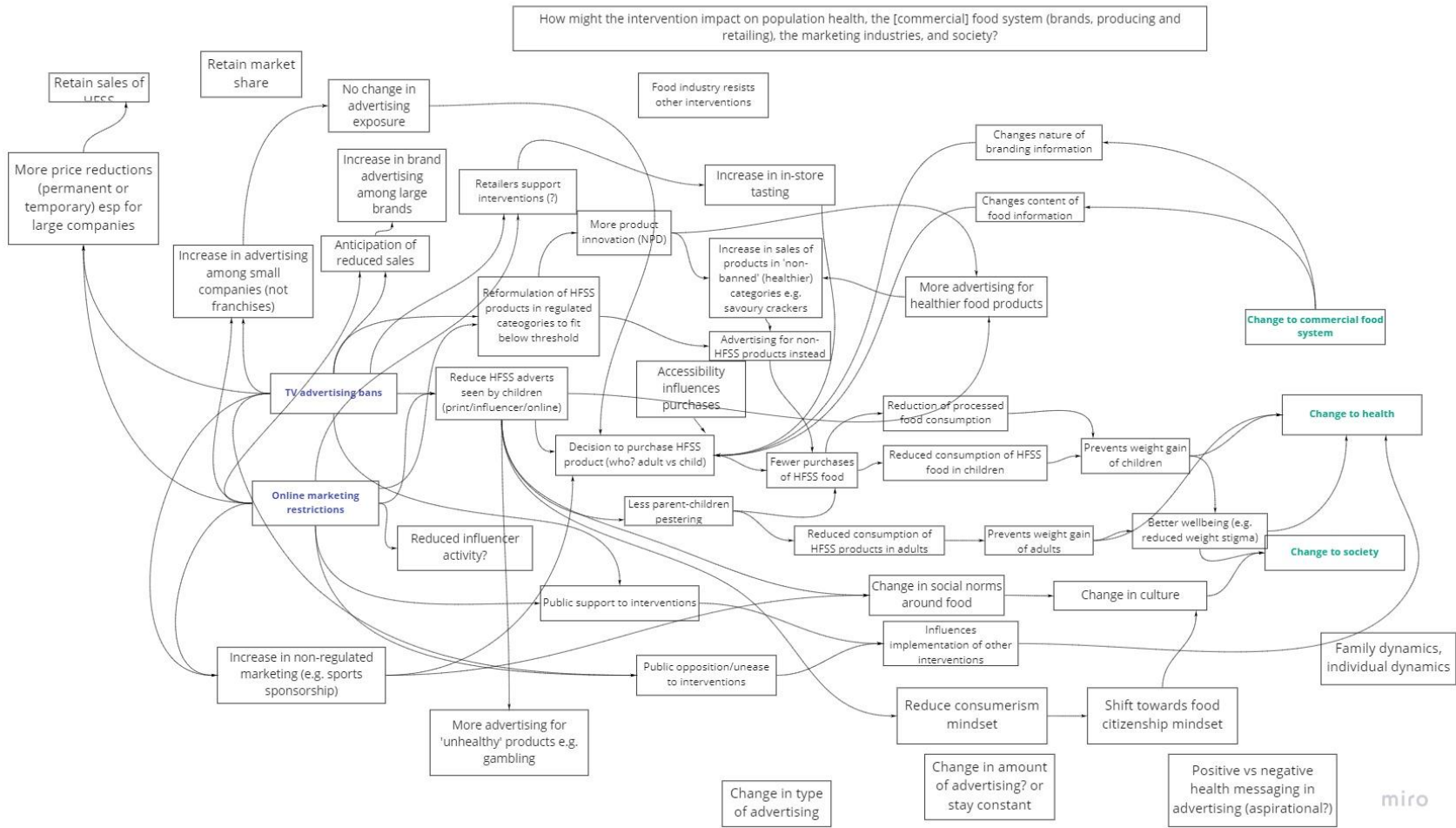

## Workshop 3 Map

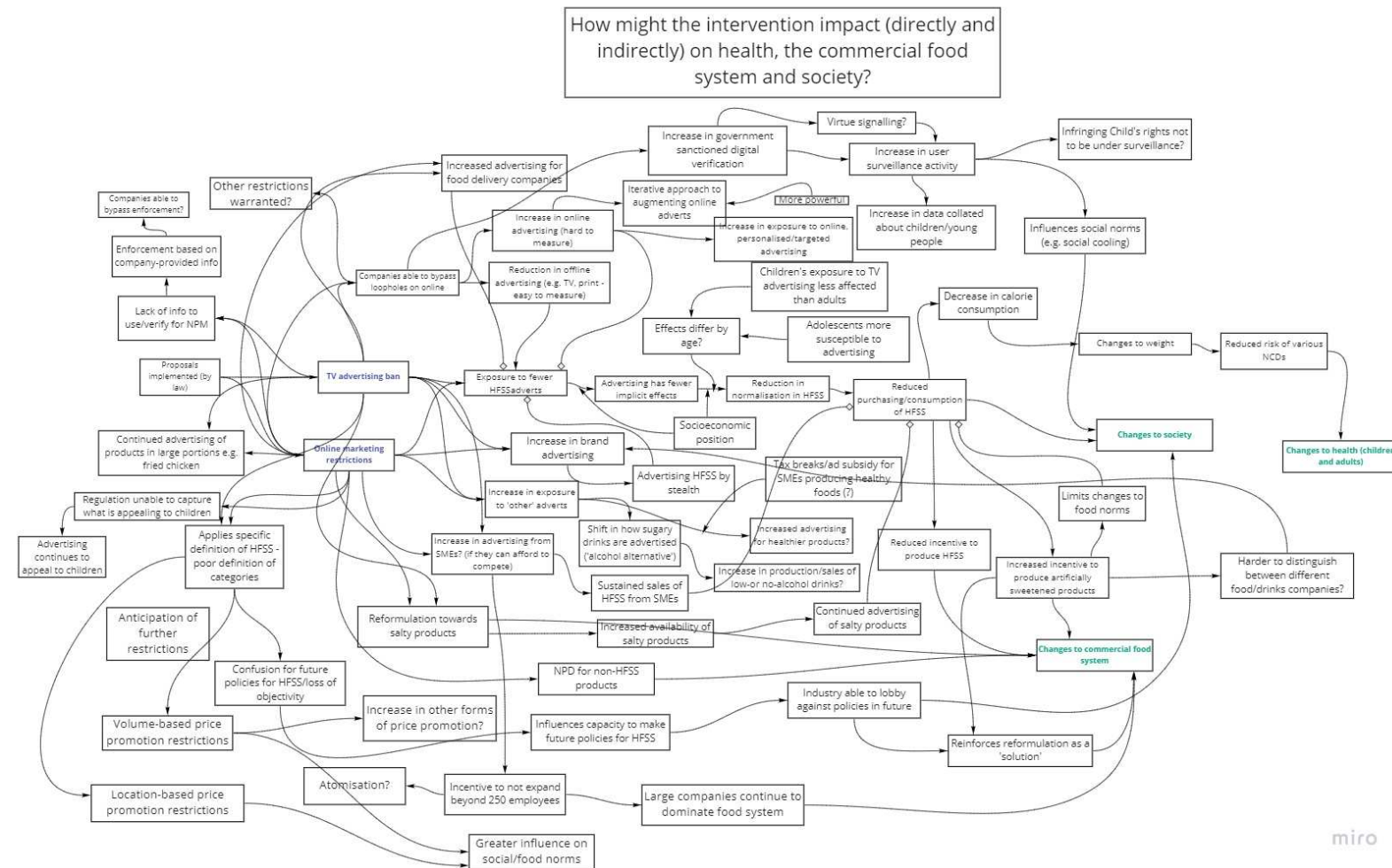

Workshop 4 Map

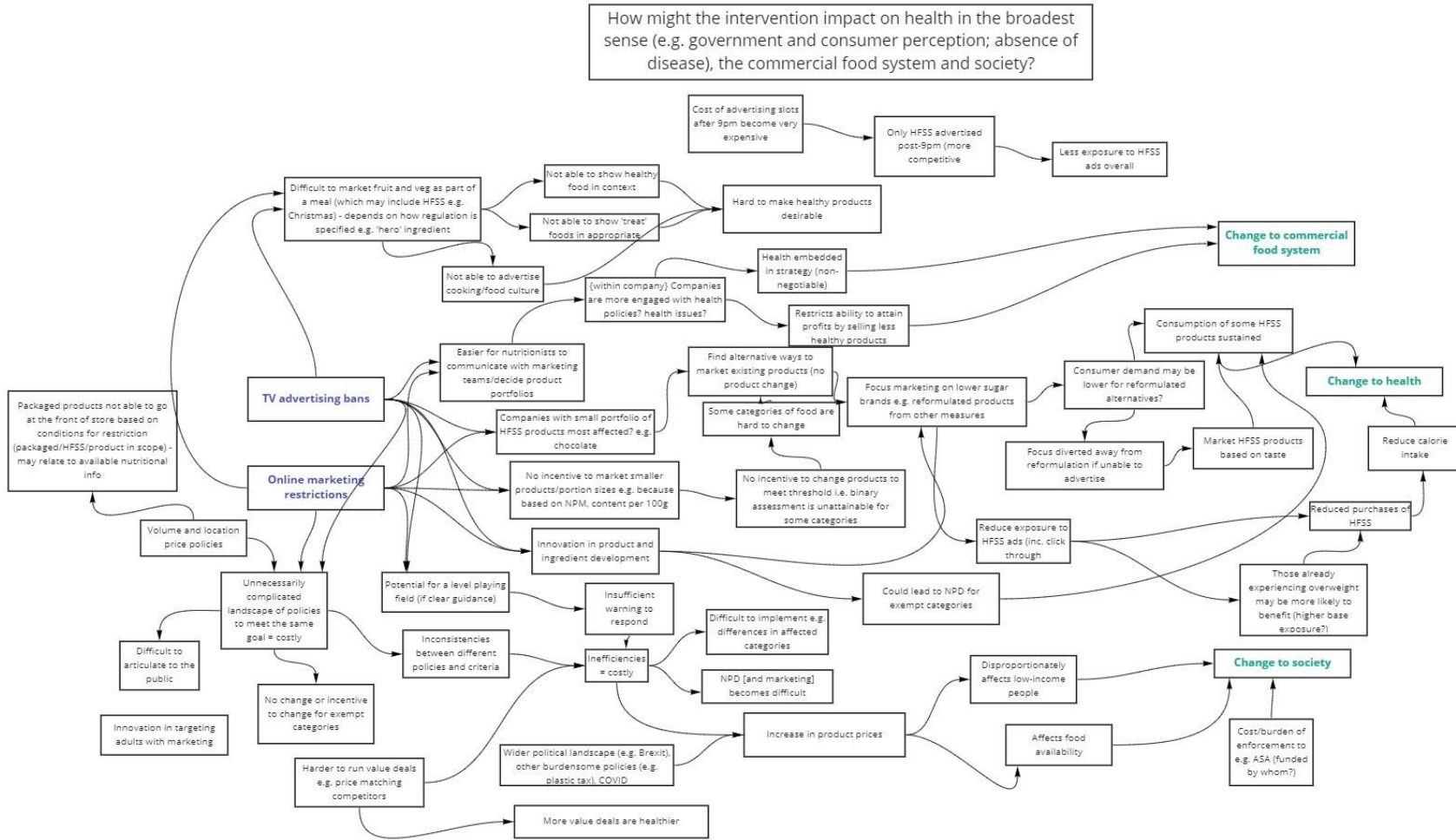

Supplement: Supplementary data [file bmjopen-2021-060302supp002.pdf]
