# Supplementary material for: Exploring the potential impact of the proposed UK TV and online food advertising regulations: a concept mapping study
Source: BMJ Open. 2022 Jun 16;12(6):e060302. doi: 10.1136/bmjopen-2021-060302 (PMC9207937; doi:10.1136/bmjopen-2021-060302)
Supplement: Supplementary data [file bmjopen-2021-060302supp003.pdf]

Appendix 3: Concepts from workshop maps and resulting concepts in the final map

Workshop 1 (non-industry)	Workshop 2 (non-industry)	Workshop 3 (non-industry)	Workshop 4 (industry)	Final map
More adept at regulations	Anticipation of reduced sales	Anticipating further restrictions		**Anticipation**
Behaviour to pre-empt? e.g., product changes		Proposals implemented by law		
Anticipate regulation				
Change product availability			Affects food availability	**Availability of HFSS products**
	Prevents weight gain of children	Changes to weight		**Bodyweight**
	Prevents weight gain of adults			
		Decrease in calorie consumption	Reduced calorie intake	**Calorie consumption**
Parent/child interactions	Less parent-children pestering			**Child purchasing requests for HFSS products**
Reduction in pester power				
Better shopping experience				
				**Commercial food system**
			{within company} companies are more engaged with health policies? Health issues?	**Company engagement with health issues**
			Health embedded in strategy (non-negotiable)	
			Easier for nutritionists to communicate with marketing teams	
Reduction in food company revenue			Restricts ability to attain profits by selling less healthy products	**Company profitability**

**Workshop 1 (non-industry)**	**Workshop 2 (non-industry)**	**Workshop 3 (non-industry)**	**Workshop 4 (industry)**	**Final map**
Change in sector profitability			Cost/burden of enforcement to e.g. ASA (funded by whom?)	
			Inefficiencies = costly	
Less screen-driven snacking	Reduced consumption of HFSS products in adults	Reduced purchasing/ consumption of HFSS	Consumption of some HFSS products sustained	**Consumption of regulated HFSS products**
Change in consumption	Reduction of processed food consumption			
Doing something else that's good for you	Reduced consumption of HFSS products in children			
Change in diet				**Consumption of unregulated products**
		Applies specific definition of HFSS - poor definitions of categories	Inconsistencies between different policies and criteria	**Definitions**
		Enforcement based on company-provided info		
		Lack of info to use/verify for NPM		
Reduction in demand				**Demand for regulated HFSS products**
Increase in money saved				
			Consumer demands may be lower for reformulated alternatives?	**Demand for unregulated products**
		Increase in user surveillance activity		**Digital surveillance**
		Infringing Child's rights not to be under surveillance?		
		Increase in surveillance activity		

Forde H, *et al. BMJ Open* 2022; 12:e060302. doi: 10.1136/bmjopen-2021-060302

**Workshop 1 (non-industry)**	**Workshop 2 (non-industry)**	**Workshop 3 (non-industry)**	**Workshop 4 (industry)**	**Final map**
		Increase in data collated about children/young people		
Changes in employment		Incentive to not expand beyond 250 employees		**Employment**
Reductions in sharing of foods on social media	No change in advertising exposure	Exposure to fewer HFSS adverts	Less exposure to ads overall	
	Reduced influencer activity?	Children's exposure to TV advertising less affected than adults	Reduce exposure to HFSS ads (inc. through click through)	
	Change in amount of advertising or stay constant	Advertising has fewer implicit effects	Market HFSS products based on taste	
	Reduce HFSS adverts seen by children	More powerful	Only HFSS advertised post-9pm (more competitive)	**Exposure to advertising for regulated HFSS products**
	Positive vs. negative health messaging in advertising (aspirational)	Virtue signalling		
	Changes content of food information	Harder to distinguish between different food/drinks companies		
		Iterative approach to augmenting online adverts		
Replaced with something else (?) i.e., not HFSS	Advertising for non-HFSS products instead	Exposure to non-HFSS adverts	Focus marketing on lower sugar brands e.g., reformulated products from other measures	
Increase advertising of F&V, healthier products in brand	More advertising for healthier food products	Increased advertising for healthier products	More value deals are healthier	
	More advertising for 'unhealthy products' e.g., gambling	Increase in exposure to 'other' adverts	Cost of advertising slots after 9pm become very expensive	**Exposure to advertising for unregulated products**
		Continued advertising of salty products		
		Increased advertising for food delivery companies		

Workshop 1 (non-industry)	Workshop 2 (non-industry)	Workshop 3 (non-industry)	Workshop 4 (industry)	Final map
Balloon effect	Change in type of advertising	Increase in exposure to online, personalised/targeted advertising	Difficult to market fruit and veg as part of a meal (which may include HFSS e.g., Christmas) - depends on how regulation is specified e.g. 'hero' ingredient	**Exposure to unregulated marketing of HFSS products**
Increase in sponsorship	Increase in non-regulated marketing (e.g., sports sponsorship)	Advertising continues to appeal to children	Not able to show healthy food in context	
'Unrelated' advertising e.g., CSR	Increase in in-store tasting	Companies able to bypass enforcement	Not able to show 'treat' foods in appropriate	
Loopholes	More price reductions especially for large companies	Increase in online advertising (hard to measure)	Hard to make healthy products desirable	
Location based promotions	Increase in advertising among small companies (not franchises)	Companies able to bypass loopholes online	Focus diverted away from reformulation if unable to advertise	
Increase in price promotions	Changes nature of branding information	Shift in how sugary drinks are advertised (alcohol alternatives)	Not able to advertise cooking/food culture	
Brand awareness reduces	Increase in brand advertising among large brands	Reduction in offline advertising (e.g., TV, print - easy to measure)	Find alternative ways to market existing products (no product change)	
Brand engagement/identification decreases		Continued advertising of products in large portions e.g., fried chicken	Innovation in targeting adults with marketing	
Brand association decreases		Advertising HFSS by stealth		
		Increase in other forms of price promotion?		
		Regulation unable to capture what is appealing to children		
	Better wellbeing (e.g., reduced weight stigma)			**Health**
Alternative evidence of efficacy	Food industry resists other interventions	Industry able to lobby against policies in future		**Lobbying against further interventions**

Forde H, *et al. BMJ Open* 2022; 12:e060302. doi: 10.1136/bmjopen-2021-060302

Workshop 1 (non-industry)	Workshop 2 (non-industry)	Workshop 3 (non-industry)	Workshop 4 (industry)	Final map
Discredit public health evidence				
Concentration of HFSS market	Retain market share	Large companies continue to dominate food system		Market share
		Atomisation?		
		Continued advertising of products in large portions e.g., fried chicken	No incentive to market smaller products/portion sizes e.g., because based on NPM, content per 100g	Portion size
Change in affordability	More price reductions especially for large companies	Increase in other forms of price promotion?	Increase in product prices	Price
Reduction in price			Harder to run value deals e.g., price matching competitors	
Diversification of non-HFSS markets	More product innovation (NPD)	NPD for non-HFSS products	Could lead to NPD for exempt categories	Product innovation for unregulated products
Positive opportunity in supply chain	Reformulation of HFSS products in regulated categories to fit below threshold	Reformulation towards salty products	Some categories of food are hard to change	
Brand diversification		Reduced incentive to produce HFSS	NPD [and marketing] becomes difficult	
New product development (reformulation?)		Increased incentive to produce artificially sweetened products	No incentive to change products to meet threshold i.e., binary assessment is unattainable for some categories	
			No change or incentive to change for exempt categories	
			Innovation in product and ingredient development	
Change in public perception				Public awareness
Awareness of regulation				
Higher awareness of harms				

Workshop 1 (non-industry)	Workshop 2 (non-industry)	Workshop 3 (non-industry)	Workshop 4 (industry)	Final map
Better perception of eating healthy especially among younger people				
	Public support for interventions		Difficult to articulate to the public	**Public support**
	Public opposition/unease to interventions			
Change in purchasing	Decision to purchases HFSS product (who? Adult vs child)	Reduced purchasing/consumption of HFSS	Reduced purchases of HFSS	**Purchases of HFSS regulated products**
	Fewer purchases of HFSS food			
	Retain sales of HFSS			
Chang e in purchasing	Increase in sales of products in 'non-banned' healthier categories e.g., savoury crackers	Increased availability of salty products		**Purchases of unregulated products**
		Increase in production/sales of low or no alcoholic drinks		
Durability of interventions	Retailers support interventions	Volume-based price promotion restrictions	Wider political landscape (e.g., Brexit) other burdensome policies (e.g., plastic tax, covid)	**Regulatory and political landscape**
Interact with other aspects of DPH policy		Location-based price promotion restrictions	Volume and location price policies	
Location based promotions		Other restrictions warranted	Unnecessarily complicated landscape of policies to meet the same goal = costly	
Covid-19 raised awareness		Reinforces reformulation as 'solution'	Potential for a level playing field (if clear guidance)	
Current events in public health		Tax breaks/ad subsidy for SMEs producing healthy foods (?)	Insufficient warning to respond	
Volume based promotions restrictions			Difficult to implement e.g., differences in affected categories	
			Packaged products not able to go at the front of store based on conditions for restrictions (packaged/HFSS/product in	

**Workshop 1 (non-industry)**	**Workshop 2 (non-industry)**	**Workshop 3 (non-industry)**	**Workshop 4 (industry)**	**Final map**
			scope) - may relate to available nutritional info)	
Legal precedent	Influences implementation of other interventions	Influences capacity to make future policies for HFSS		**Regulatory precedent**
Decision norming (more regs seem possible)		Confusion for future policies for HFSS/loss of objectivity		
Empower policymakers		Increase in government sanctioned digital verification		
		Reduced risk of various NCDs		**Risk of diet-related NCDs**
Fewer social connotations around food	Change in social norms around food	Influences social norms e.g., social cooling		**Social norms around food**
Changes in social norms	Family dynamics, individual dynamics	Reduction in normalisation of HFSS		
Change in engagement with foods		Limits changes to food norms		
Associations with young people change (e.g., aspirational)		Greater influence on social/food norms		
				**Society**
Changes link between food and personal identity	Shift towards food citizenship mindset			**Societal shifts**
Different retail environment	Reduce consumerism mindset			
Less time spent on social media	Change in culture			
Polarisation				
Small producers meeting nutritional criteria/ambient food increase sales	Accessibility influences purchases	Increase in advertising from SMEs? If they can afford to compete	Companies with small portfolio of HFSS products most affected? e.g., chocolate	**Changes vary by…**
Different price sensitivity	Increase in advertising among small companies (not franchises)	Sustained sales of HFSS from SMEs	Those already experiencing overweight may be more likely to benefit (higher base exposure?)	
Importance of brand amplified for low-income people		Effects differ by age?	Disproportionately affects low-income people	

Workshop 1 (non-industry)	Workshop 2 (non-industry)	Workshop 3 (non-industry)	Workshop 4 (industry)	Final map
Importance of brand amplified for young people		Socioeconomic position		
Different groups more susceptible e.g., those exposed		Adolescents more susceptible to advertising		
Differential effects by socioeconomic position				
Baseline differences in health (socioeconomic gradient				
Reduce health inequalities				
Distinguish effects by brand strength e.g., better for established brands				

Notes: HFSS: high fat, salt and sugar; NCD: non-communicable disease; NPD: new product development; NPM: Nutrient Profile Model; SME: small and medium enterprises.

Forde H, *et al. BMJ Open* 2022; 12:e060302. doi: 10.1136/bmjopen-2021-060302
