# Supplementary material for: Exploring the potential impact of the proposed UK TV and online food advertising regulations: a concept mapping study
Source: BMJ Open. 2022 Jun 16;12(6):e060302. doi: 10.1136/bmjopen-2021-060302 (PMC9207937; doi:10.1136/bmjopen-2021-060302)
Supplement: Author's manuscript [file bmjopen-2021-060302.draft_revisions.pdf]

BMJ Open is committed to open peer review. As part of this commitment we make the peer review history of every article we publish publicly available.

When an article is published we post the peer reviewers' comments and the authors' responses online. We also post the versions of the paper that were used during peer review. These are the versions that the peer review comments apply to.

The versions of the paper that follow are the versions that were submitted during the peer review process. They are not the versions of record or the final published versions. They should not be cited or distributed as the published version of this manuscript.

BMJ Open is an open access journal and the full, final, typeset and author-corrected version of record of the manuscript is available on our site with no access controls, subscription charges or pay-per-view fees (http://bmjopen.bmj.com).

If you have any questions on BMJ Open's open peer review process please email info.bmjopen@bmj.com

BMJ Open

Exploring the potential impact of the proposed UK TV and online food advertising regulations: a concept mapping study

Journal:	*BMJ Open*
Manuscript ID	bmjopen-2021-060302
Article Type:	Original research
Date Submitted by the Author:	17-Dec-2021
Complete List of Authors:	Forde, Hannah; MRC Epidemiology Unit<br>Boyland, Emma; University of Liverpool Institute of Population Health, Department of Psychology<br>Scarborough, Peter; University of Oxford, Nuffield Department of Population Health; NIHR Oxford Biomedical Research Centre<br>Smith, Richard ; University of Exeter Medical School, Institute of Health Research<br>White, Martin; MRC Epidemiology Unit<br>Adams, J; MRC Epidemiology Unit
Keywords:	PUBLIC HEALTH, NUTRITION & DIETETICS, QUALITATIVE RESEARCH

SCHOLARONE™
Manuscripts

I, the Submitting Author has the right to grant and does grant on behalf of all authors of the Work (as defined in the below author licence), an exclusive licence and/or a non-exclusive licence for contributions from authors who are: i) UK Crown employees; ii) where BMJ has agreed a CC-BY licence shall apply, and/or iii) in accordance with the terms applicable for US Federal Government officers or employees acting as part of their official duties; on a worldwide, perpetual, irrevocable, royalty-free basis to BMJ Publishing Group Ltd ("BMJ") its licensees and where the relevant Journal is co-owned by BMJ to the co-owners of the Journal, to publish the Work in this journal and any other BMJ products and to exploit all rights, as set out in our licence.

The Submitting Author accepts and understands that any supply made under these terms is made by BMJ to the Submitting Author unless you are acting as an employee on behalf of your employer or a postgraduate student of an affiliated institution which is paying any applicable article publishing charge ("APC") for Open Access articles. Where the Submitting Author wishes to make the Work available on an Open Access basis (and intends to pay the relevant APC), the terms of reuse of such Open Access shall be governed by a Creative Commons licence – details of these licences and which Creative Commons licence will apply to this Work are set out in our licence referred to above.

Other than as permitted in any relevant BMJ Author's Self Archiving Policies, I confirm this Work has not been accepted for publication elsewhere, is not being considered for publication elsewhere and does not duplicate material already published. I confirm all authors consent to publication of this Work and authorise the granting of this licence.

For peer review only

Exploring the potential impact of the proposed UK TV and online food advertising regulations: a concept mapping study

Hannah Forde[a*], Emma Boyland[b], Peter Scarborough[c,d], Richard Smith[e], Martin White[a], Jean Adams[a]

[a] MRC Epidemiology Unit, University of Cambridge School of Clinical Medicine, Box 285 Institute of Metabolic Science, Cambridge Biomedical Campus, Cambridge, CB2 0QQ

[b] Department of Psychology, Institute of Population Health, University of Liverpool, Eleanor Rathbone Building, Bedford Street South, Liverpool, L69 7ZA

[c] Nuffield Department of Population Health, University of Oxford, Old Road Campus, Roosevelt Drive, Headington, Oxford, OX3 7LF

[d] NIHR Oxford Biomedical Research Centre, The Joint Research Office, Second Floor, OUH Cowley Unipart House Business Centre, Garsington Road, Oxford, OX4 2PG

[e] Medical School Building, College of Medicine and Health, University of Exeter, St Luke's Campus, Heavitree Road, Exeter, EX1 2LU

*Correspondence to Hannah Forde: hf332@medschl.cam.ac.uk

MAIN MANUSCRIPT WORD COUNT: 4623

For peer review only

For peer review only - http://bmjopen.bmj.com/site/about/guidelines.xhtml

ABSTRACT

Objectives In July 2020 the UK Government announced an intention to restrict advertisements for products high in fat, salt and sugar on live broadcast, catch-up and on-demand television before 9pm; and paid for online advertising. As no other jurisdiction has implemented similar regulations, there is no empirical evidence about how they might perturb the food system. To guide the regulations' implementation and evaluation, we aimed to develop a concept map to hypothesise their potential consequences for the commercial food system, health and society.

Methods We used adapted group concept mapping in four workshops virtually with food marketing and regulation experts across academia, civil society, government organisations, and industry (N=14), supported by Miro software. We merged concepts derived from the four workshops to develop a master map and then invited feedback from participants via email to generate a final concept map.

Results The concept map shows how the reactions of stakeholders to the regulations may reinforce or undermine the impact on the commercial food system, health and society.

Conclusions We use the concept map to illustrate pathways in three potential scenarios: (i) adaptations are made to the regulations in ways that reinforce positive impacts on public health; (ii) adaptations are made to the regulations in ways that undermine impacts on public health; and (iii) technicalities of the regulations cover too few unhealthy food products and advertising opportunities to make a substantial difference to public health.

Prior to the regulations' initial implementation or subsequent iterations, they could be altered to maximise the potential for reinforcing adaptations, minimise the potential for undermining adaptations, and ensure they cover a wide range of advertising opportunities and foods. The concept map will also inform the design of an evaluation of the regulations and could be used to inform the design and evaluation of similar regulations elsewhere.

STRENGTHS AND LIMITATIONS OF THIS STUDY

- By including a diverse range of experts, we developed the first comprehensive articulation of the potential pathways through which new advertising regulations may impact on the commercial food system, health and society.
- Holding the workshops online may have facilitated greater attendance, particularly as we employed techniques to minimise the limitations of online data collection.
- Timing the workshops after sufficient details are known about the regulations allowed for a meaningful discussion about their impact but with enough time for the study's findings to feed into the regulations' design.

For peer review only

- Though we did not aim to achieve saturation in this study, we found it difficult to recruit participants from industry.
- We necessarily invited more individuals than those who ultimately participated, which may affect the transferability of the study's findings.

For peer review only

For peer review only - http://bmjopen.bmj.com/site/about/guidelines.xhtml

INTRODUCTION

The World Health Organization (WHO) recommends that member states limit children's exposure to marketing for less healthy foods.[1] The recommendation reflects evidence that marketing influences food preferences and consumption, both at an individual (micro-level impacts)[2,3] and societal level (macro-level impacts).[4] Marketing has been defined as "the activity, set of institutions, and processes for creating, communicating, delivering, and exchanging offerings that have value for customers, clients, partners, and society at large".[5] Marketing is exerted through a range of activities, including those related to the product, its place, price and promotion.[6] Promotion includes building games around products (advergames), social media 'influencers', and paid for advertising in any medium. Products high in fat, salt, or sugar (HFSS) are disproportionately advertised in the UK, with only 2.5% of total food and soft drink advertising spend going towards fruit and vegetables in 2020.[7] Though the causal pathways between advertising and obesity are likely to be complex,[8] it is estimated that 6.4% (95% confidence interval (CI): 2.0-13.8) of UK childhood obesity and 5.0% (95% CI: 1.5-10.9) of overweight is attributable to HFSS television advertising alone.[9]

To address concerns about the prevalence of childhood obesity, in July 2020 the United Kingdom's (UK) Government Department of Health and Social Care published an intention to restrict advertisements for HFSS food and drink products on live broadcast, catch-up and on-demand television ('TV') before 9pm and paid for online advertising ('online').[10] Current details of these proposed regulations are summarised in **Box 1**, and though they have passed through the House of Lords in the Health and Care Bill,[11] details of the regulations may change before they receive Royal Assent and are implemented.

INSERT BOX 1 HERE

The TV and online regulations proposed for the UK will be some of the most restrictive worldwide, and the first to explicitly address paid for online advertising.[12] Overall, 18% of UK advertising spend is for TV slots and at least 63% for online slots.[13] Though there has been a recent decline in broadcast TV viewing in the UK, average viewing time remains around three hours per day for ages 4 years and above.[13] The Covid-19 pandemic has accelerated use of subscription video-on-demand services, with viewing of services such as Netflix and Amazon Prime Video almost doubling in 2020 to an estimated 1 hour per person per day.[14] Such services would be covered by the proposed online regulation rather than the TV one. While the decline in broadcast TV viewing has been more pronounced among younger people (for 16-24 year olds down 18%, and for children 4-15 year olds down 16% in 2019),[13] this has corresponded with an increase in viewing of subscription video-on-demand services among younger people (by 55 minutes to an average of two hours per day between

For peer review only - http://bmjopen.bmj.com/site/about/guidelines.xhtml

April 2019 and April 2020).[14] It has been estimated that a pre-9pm ban on HFSS TV food advertising would result in a 4.6 (1.4-9.5)% reduction in childhood obesity and a 3.6 (1.1-7.4)% reduction in childhood overweight prevalence.[9] Effects were two-fold greater in the least compared to the most affluent social groups and would likely be amplified by comparable restrictions on online food promotion.[9] The ultimate results of such a regulation were predicted to depend on how HFSS advertising patterns change in response.[9]

Few evaluations of such food advertising restrictions have been conducted worldwide,[12] partly because there have been few comparable regulations. There are also challenges to evaluating this type of intervention that is delivered to whole populations and so is impractical to subject to experimental evaluation techniques such as randomised controlled trials.[15] Furthermore, the commercial food sector exhibits characteristics of a complex adaptive system.[16] Adaptations made by stakeholders residing in the system that is regulated may lead to both intended and unintended consequences that ultimately impact on the overall effectiveness of regulations.[16] The 'balloon effect' proposes that restrictions on one type of marketing can lead to increases in others,[17] as companies and other aspects of the food system adapt. Articulating these possible adaptations and their potential consequences should help refine details of the regulations before implementation. Understanding possible adaptations and consequences should also help inform the design of any evaluation.

To maximise the applicability of evaluation findings to policymakers outside of the UK, it is helpful for evaluators to test theories as well as evaluate interventions.[18] Theory-driven evaluation first requires the development and clear articulation of program theory.[19] Concept mapping is an approach particularly useful for public health researchers interested in developing theory.[20] A concept map is a "diagram of proposed relationships among a set of concepts….about a particular question….or topic".[21] Concept maps can be used to help organise ideas, demarcate an area of interest and plan evaluations. Group concept mapping is a structured approach involving group work that is flexible to many public health contexts.[22]

Objectives

In this study, we used an approach inspired by group concept mapping to develop a concept map of how the new TV and online advertising regulations may impact on the commercial food system, health and society. We aimed to describe how the regulations may interact with the food system so that evaluations of the regulations can be grounded in clearly articulated theory, and so that adaptations to the regulations that could improve the health impact can be identified before implementation.

For peer review only - http://bmjopen.bmj.com/site/about/guidelines.xhtml

METHODS

Study design

We created a concept map of the potential pathways through which the regulations may impact on the commercial food system, health and society. By 'food system' we mean the interdependent network of entities involved in agriculture and fisheries, food processing and production, storage and distribution, wholesaling and retailing, and preparation and marketing of raw, processed and ready to eat foods.[16] By 'society', we mean the wider social system in which the food system is embedded. We developed the map using an adapted version of a group concept mapping method in four workshops.[22] The study reporting adheres to the Consolidated Criteria for Reporting Qualitative Research (COREQ) (**Appendix 1**),[23] but recognises proposed amendments relating to gender.[24]

Participant recruitment

Workshop participants were recruited from academia, civil society, government organisations and industry (e.g., food industry, media, advertising). Individuals were eligible for inclusion if they had professional knowledge and experience of food marketing regulation within their sector and were based in the UK. We identified individuals from our existing contacts in these sectors and by searching the websites of relevant organisations. Individuals were invited by email to take part in the study. We aimed to recruit up to 20 individuals, approximately evenly distributed across the participant groups. As we were not aiming to reach 'saturation',[25] we decided on the number of people to recruit to the study pragmatically, based on the resources available to us but allowing for sufficient breadth.

Participants from industry attended a separate workshop to those from academia, civil society and government organisations due to the potential for conflicts of interests between sectors. We set a limit of 10 participants per workshop in addition to the facilitators (JA and HF, who both had qualitative research experience, e.g., [26,27]), which is considered a manageable total number of participants to permit dialogue and engagement.[22] Workshops were arranged around participants' availability in July and August 2021 and lasted 2 hours each.

Data collection

Building on previous work that has used group concept mapping to inform the design of evaluations of population health interventions,[28] we used the first three steps of group concept mapping (preparation, generation and structuring)[22] and added a fourth (reflection). The first three steps were achieved in the workshops, and the final step was achieved using an online feedback form. We held the workshops on Zoom, an online videoconferencing software (https://zoom.us/) to minimise time demands on participants and as data collection took place during COVID-19 restrictions. In the

For peer review only - http://bmjopen.bmj.com/site/about/guidelines.xhtml

workshops, we used a combination of pre-piloted Microsoft PowerPoint slides and Miro software (https://miro.com/) to provide instructions to participants and visualise their contributions as they were made, respectively. Our data consisted of screenshots of maps as they developed, the map from each workshop, audio recordings of the workshops, and post-workshop feedback returned through an online form. Workshops were held under the Chatham House Rule[29]: participants were told they could use the information discussed in the workshops, but they could not reveal the identity or affiliation of other participants. **Figure 1** summarises the method used to develop the final concept map.

INSERT FIGURE 1 HERE

Preparation

Preparation entailed setting out the aims and processes of the workshop and agreeing the focus area of the map.[22] At the beginning of each workshop, the workshop facilitators introduced the aims and processes. They reminded participants of the intervention details, the withdrawal process and that the workshops were being recorded. The facilitators proposed that the focus area was "what are the potential pathways through which the intervention might impact on health, the commercial food system and society?". Participants were invited to help refine this during a discussion of approximately 5 minutes.

Generation

Generation is a divergent process where participants individually brainstorm a long list of responses to the focus area and consider the relative importance of each response.[22] Participants were given around 10 minutes to independently generate a list of as many responses as possible to the refined focus area, including pathways to both positive and negative impacts arising from the regulations.

Structuring

Structuring is a convergent process where participants organise and critically reflect on ideas and relationships between concepts.[22] For approximately 60 minutes, participants were asked in turn to contribute responses to the focus area from their individual brainstorming in order of relative importance. These were structured and visualised in real-time using Miro, which was shared on-screen with participants, with new concepts and relationships added to a draft map as participants suggested them (see **Figure 2**). Once all responses were included, participants were invited to reflect on the map, adding additional concepts and relationships as required. We adopted an inclusive approach to adding concepts and relationships to maps, including everything mentioned and not deleting anything previously added.

For peer review only - http://bmjopen.bmj.com/site/about/guidelines.xhtml

INSERT FIGURE 2 HERE

Reflection

After the workshops, we merged the map from each workshop into one ‘master’ map. We used a method inspired by those employed in other mapping projects.[30] First, all concepts in the maps were documented in a Microsoft Excel sheet, and similar or identical concepts across the maps were grouped and refined into simplified concepts and accompanying descriptions. Second, these refined concepts were mapped in a way that corresponded with pathways depicted in the four separate maps. Concepts not immediately fitting anywhere were placed to the side for further deliberation.

We then circulated the master map to all workshop participants by email. The email contained a link to an online form issued via REDCap (https://www.project-redcap.org/) that asked questions about the map to seek suggested changes. We used the suggestions to produce a final concept map.

Analysis

Beyond merging the maps from each workshop into a master map, no formal analyses were conducted.

Ethics

The study received favourable review from the University of Cambridge School of Humanities and Social Science Research Ethics Committee in June 2021, reference number 21.276. Participants were provided with an information sheet about the study and provided informed consent before joining a workshop using an e-consent form issued via REDCap.

Patient and public involvement

Patients and/or the public were not involved in the design, conduct, reporting or dissemination plans of this research.

RESULTS

From four workshops with a total of 14 participants, we developed a concept map to describe how the proposed TV and online advertising regulations may impact on the commercial food system, health and society. Here we present the concept map and describe its component concepts.

For peer review only

For peer review only - http://bmjopen.bmj.com/site/about/guidelines.xhtml

Participant characteristics

We held four workshops: one with individuals from industry, and three with individuals from academia, civil society, and government organisations (see **Table 1**). As the focus was on generating the map as a group, we did not collate any demographic information about participants.[30]

Table 1 Sectors included in each workshop

Participant sectors per workshop	Workshop 1	Workshop 2	Workshop 3	Workshop 4	Total
Academia	2	1	1	0	4
Civil society	2	1	3	0	6
Government organisation	0	1	1	0	2
Industry	0	0	0	2	2
				**Grand total**	14

Concept map of anticipated adaptations to the regulations

The maps produced in each workshop are provided in **Appendix 2**, and they illustrate the nuance in focus between workshops. For example, the workshop with industry participants focused more on the technical difficulties presented by the regulations than in other workshops. The resultant conceptmap is presented in **Figure 3,** and it depicts the possible pathways of change that could follow the regulations**.** Colour coding is used to differentiate the groups of reactions to the regulations: government, food and beverage companies, public, society and health. Pathways depicted are not exhaustive, as it is possible that other links between concepts exist that were not captured in the workshops. The map is also accompanied by a list of factors that may modify the impact of pathways that it depicts, such as socioeconomic position and company size. The concepts contained in each workshop map, and the corresponding concepts they were assigned in the final concept map, are provided in **Appendix 3**. Concepts are described in more detail in **Table 2**.

INSERT FIGURE 3 HERE

Table 2 Description of concepts in the concept map

Statement	Description
**Anticipation**	Food and drink companies foresee the introduction of the regulations[a], and possibly other related legislation e.g., volume and location price promotion.
**Availability of HFSS products**	Availability of all HFSS foods and beverages, both within and outside the scope of the regulations[a], in physical and online shops.
**Bodyweight**	In terms of Body Mass Index (BMI), overweight or obesity status.

For peer review only - http://bmjopen.bmj.com/site/about/guidelines.xhtml

Statement	Description
**Calorie consumption**	Total energy intake of individuals.
**Child purchasing requests for HFSS products**	Degree to which children make purchasing requests to caregivers for all HFSS products, both within and outside the scope of the regulations[a].
**Commercial food system**	Interdependent networks of commercial entities involved in agriculture and fisheries, food processing and production, storage and distribution, wholesaling and retailing, and preparation and marketing of raw, processed, and ready to eat foods.[16]
**Company engagement with health issues**	Degree to which food and beverage companies orientate their business around public health goals.
**Company profitability**	A company's ability to make profit.
**Consumption of regulated HFSS products**	Individual's intake of foods and beverages within the scope of the regulations[a].
**Consumption of unregulated products**	Individual's intake of foods and beverages that are not within the scope of the regulations[a].
**Definitions**	Information used to define or enforce the regulation[a], including the UK Nutrient Profiling Model and the food categories form the Sugar Reduction Strategy. Importantly, the regulations[a] cover a group of foods that is different from those covered by other UK dietary public health regulations. Enforcement is based on information provided by companies.
**Demand for regulated HFSS products**	Public desire to purchase or consume foods and beverages within the scope of the regulations[a].
**Demand for unregulated products**	Public desire to purchase or consume foods and beverages outside of the scope of the regulations[a].
**Digital surveillance**	Digital data collated by website to inform regulation[a] enforcement.
**Employment**	Number of people employed in the commercial food system.
**Exposure to advertising for unregulated products**	Adverts for products outside of the scope of the regulations. For foods and beverages, this could be HFSS products within companies' portfolios that are outside of the scope of the regulations, healthier products (e.g., fruit and vegetables), or food delivery companies. Also includes non-food and beverage products and services, but not clear what health impacts they might have.
**Exposure to advertising for regulated HFSS products**	Exposure to advertising for food and beverages within the scope of the regulations[a].

For peer review only - http://bmjopen.bmj.com/site/about/guidelines.xhtml

Statement	Description
**Exposure to unregulated marketing of HFSS products**	Exposure to advertising for all HFSS products on media that are outside of the scope of the regulations[a]. Includes offline advertising (e.g., print media), forms of marketing online that are exempt from the regulations (e.g., in owned media), sponsorship, brand advertising and creative modes of marketing that are hard to capture with regulation.
**Health**	Overall health, including and beyond bodyweight and non-communicable diseases (NCDs).
**Lobbying against further interventions**	Activities undertaken by, or on behalf of, food and beverage companies to resist further policy or regulations.
**Market share**	The size of the total market held by a company. Few companies that each hold a large market share creates a concentrated market.
**Portion size**	Size of food and beverage products in grams or calories, or recommended portion size.
**Price**	Price of food and beverage products, including price discounts.
**Product innovation for unregulated products**	Developing new products that are outside of the scope of the regulations[a], or reformulating existing products so they are no longer within the scope of the regulations. Could include reformulation using artificial ingredients or developing e.g., saltier products that are currently an exempt category. Some categories of products are easier to change than others, and some companies are better able to respond in this way than others.
**Public awareness**	Degree of public awareness of both the regulations[a] and the problems they are trying to address.
**Public support**	Degree of public support for the regulations[a].
**Purchases of regulated HFSS products**	Sales (from company perspective) or purchases (from individual perspective) of food and beverage products within the scope of the regulations[a].
**Purchases of unregulated products**	Sales (from company perspective) or purchases (from individual perspective) of food and beverage products outside of the scope of the regulations[a].
**Regulatory and political landscape**	Wider landscape of regulation and policy, including others relating to marketing (e.g., location and volume price regulations) and COVID-19. The degree to which the regulations[a] harmonise with the wider political and regulatory landscape.
**Regulatory precedent**	Implementation of the regulations[a] serves as precedent for any future regulation.
**Risk of diet-related NCDs**	Risk of developing NCDs influenced by dietary behaviours.
**Social norms around food**	Implicit or explicit beliefs, attitudes, or behaviours about eating, at both an individual and family level.

For peer review only - http://bmjopen.bmj.com/site/about/guidelines.xhtml

Statement	Description
**Society**	The wider social system in which the food system is embedded.
**Societal shifts**	Exposure to advertising effects social norms and may contribute to societal changes in consumerism and culture.

Notes: BMI = body mass index; HFSS = high fat, salt, and sugar; NCD = Non-communicable disease.

[a] The regulations apply to online and TV advertising for a subset of HFSS products, defined by the 2004 to 2005 UK Nutrient Profiling Model and within particular categories from the Sugar Reduction Strategy. This means there are HFSS products (unregulated HFSS) and non-HFSS products outside of the scope of the regulations.

DISCUSSION

Overview of findings

Using an adapted group concept mapping method in four expert workshops, we developed a concept map to visualise how the proposed TV and online food advertising regulations may impact on the commercial food system, health and society. The concept map illustrates that the pathways between the regulations and these impact domains will be determined by the reactions of stakeholders.

Strengths and limitations

To our knowledge, this is the first cross-sectoral attempt to explicitly theorise how regulations of this kind may impact on the commercial food system, health and society. Incorporating the views of a range of experts with different perspectives and interests allowed us to create a comprehensive articulation of the ways the regulations may positively or negatively affect public health. As with any qualitative research, our map does not claim to be representative of views of the wider groups that participants represent.[30] Instead, we intended to sample a diverse range of expert views related to food marketing and its regulation. Including participants from diverse sectors is a strength of the study as it enabled the proposed regulations to be theorised expansively.

We necessarily invited more individuals than those who ultimately participated. The timing of the data collection period was a common reason for non-participation in the workshops, as it coincided with summer and school holidays in the UK, which may have made it difficult for those with child caring responsibilities to attend. To accommodate individuals' other commitments, we held smaller workshops across various times and days. Doing so increased the participation in our study, but it may have lost some discussion and synergy that larger groups allow.

For peer review only - http://bmjopen.bmj.com/site/about/guidelines.xhtml

We found it difficult to recruit individuals from industry and government organisations. Employees from these sectors rarely have their contact details listed on public-facing websites, unlike those from academia and civil society. Government organisations expressed reluctance to contribute information beyond what was already in the public domain.[31] There may have also been reluctance from industry to engage with our research due to inherent differences between the goals of public health researchers and of the food industry. Industry perspectives in our study may therefore be more sympathetic to public health goals than those of the wider sector. Participants may have also taken part in our study to pursue their own agenda, as industry actors have previously sought to undermine food advertising regulations.[32,33] There are some differences in the contributions made by industry participants compared with non-industry ones (**Appendix 2** and **3**). However, the nature of the workshop content, holding workshops with experts from non-industry sectors, and verifying findings with all participants, left little room for industry interests to overly-dominate our concept map.

Conducting the workshops in person may have achieved different results, as some participants may have felt more able to share sensitive information in person. However, online workshops widened attendance to those who would have been unable to attend in-person. To avoid some of the potential challenges of collecting data using Zoom, we employed several recommended strategies.[34] This included using screen-sharing and clear greetings to develop rapport, using back-up recording devices, holding facilitator briefings to avoid technical issues, and establishing 'house rules' to ease participants' experiences.[34] To maintain participant engagement, workshop duration was limited to two hours, and primarily focused on capturing concepts rather than exhaustively detailing the pathways between them. Though it may have increased participant fatigue and burden, holding longer workshops may have allowed time to capture additional concepts and pathways. As a form of member-checking,[35] we verified the master map with all workshop participants by email, in a further attempt to ensure the final concept map accurately represented participants' contributions and to allow additional comments.

Interpretation of findings

The concept map can be used to illustrate pathways through which the reactions of food and drink companies may serve or undermine the public health goals of the regulations. Here, we describe three potential scenarios: (i) adaptations are made to the regulations in ways that reinforce positive impacts on public health; (ii) adaptations are made to the regulations in ways that undermine impacts on public health; and (iii) technicalities of the regulations cover too few unhealthy food products and advertising opportunities to make a substantial difference to public health. As it is unlikely all companies will

For peer review only - http://bmjopen.bmj.com/site/about/guidelines.xhtml

respond uniformly, a combination of the three scenarios may follow the implementation of the regulations.

Scenario 1: adaptations reinforce positive impacts of the regulations on public health

Companies may reduce their TV and online advertising for regulated HFSS products, as they will have less opportunity for advertisements. Doing so reduces people's exposure to HFSS adverts, which may prompt corresponding reductions in demand, purchases and consumption of the associated HFSS products. Consequently, this will reduce the total number of calories consumed by individuals, improving health outcomes both associated with, and independent of, body weight.

To make up lost revenue from fewer HFSS product purchases, companies may increase TV and online advertising for their products that are out of the scope of the regulations (e.g., 'spotlighting' low-fat, -salt and -sugar alternatives). They may also engage with diet-related health issues, which could include developing and advertising new products that are out of scope of the regulations, particularly if there is public support for the regulations and corresponding falls in demand for HFSS products. Doing so reduces the proportion of HFSS products (relative to non-HFSS) available in the food system.

Reduced exposure to HFSS adverts may change social norms about the acceptability of consuming HFSS products. It may also change a consumerism mindset that may be encouraged by adverts to over-purchase and consume products. These changes could contribute to societal shifts that reinforce lower demand for HFSS products and change macro-level eating behaviours.

Scenario 2: adaptations undermine impacts of the regulations on public health

Food and drink companies could also minimise losses incurred by the regulations by redirecting their efforts towards unregulated forms of marketing ('balloon effect'). Companies could increase their expenditure on brand advertising, sports sponsorship, or advertising outdoors or in print or audio media, none of which are intended to be covered by the regulations. It is unclear how this may affect people's total exposure to marketing, and their resultant demand for HFSS products. Companies may also fear the implementation of further regulations that could affect their performance, and so may lobby against them. Lobbying could change future regulations such that their impact is limited, and in turn, may mean that other, comparable regulations also have less chance of being implemented.

To implement regulations, companies may increase the amount of data they collect about the population. Such data gathering constitutes greater digital surveillance that impacts society (for example, privacy rights),[36] but could also inform more targeted marketing that is known to be highly effective at encouraging sales and consumption.[37–39]

For peer review only - http://bmjopen.bmj.com/site/about/guidelines.xhtml

Scenario 3: technicalities hinder potential impacts of the regulations on public health

The regulations have a specific set of HFSS withing scope, which has notable exemptions such as some salty foods. TV and online advertising for products exempt from the regulations may continue, as may the corresponding purchasing and consumption of these products. Some participants reported that the proposed scope of the regulations differ to that of other policies. Lack of consistency with other regulations may make it costly – perhaps to the point of being futile – for companies to respond to the regulations by developing new products that are compliant with all related regulations. Limited development of new products would restrict the degree of transformation in the food system. Furthermore, unlike other regulations, these advertising regulations are not defined by portion size nor are smaller portion sizes an explicit objective of the regulations. This means there is no incentive for companies to produce smaller product sizes, which could otherwise contribute towards reducing calorie consumption via HFSS products.

As advertising by small and medium enterprises are also exempt from the regulations, larger companies may 'atomise' by creating smaller off-shoot companies, which can continue to advertise and sell HFSS products without limitation by the regulations. Advertising of HFSS outside of the watershed hours will still be permitted on TV and on-demand services, and large HFSS companies can afford the high price of advertising slots likely to occur after 9pm. TV advertising after 9pm may therefore become saturated with HFSS products, which may limit the impact of the regulations on adults' and older teenagers' consumption habits and, by extension, that of the children they are responsible for.

Comparison to existing literature

Many existing models exist to illustrate how food marketing affects behaviour and health (e.g.,[8]) and logic models are regularly produced to illustrate how other diet-related public health regulations may work. Methods for developing such models have evolved to appreciate the complexity of the surrounding system in which they reside,[40] but to our knowledge, these have been rarely applied in the context of diet-related health interventions,[41] and not applied to food advertising regulations before. The concept map we developed here is the first we are aware of to show how food marketing regulations may work by interacting with their surrounding system.

The concept map we developed illustrates ways that reactions to the regulations will reinforce or undermine their impact on public health, reinforcing the hypotheses of earlier work. [9] The potential for some of these pathways to exist has been evidenced elsewhere. Analyses have found that 57 of 65 brands associated with HFSS had an easily identifiable HFSS product, and the majority (84%) of

For peer review only - http://bmjopen.bmj.com/site/about/guidelines.xhtml

these products had an alternative non-HFSS product from the same brand, master brand, parent company, or license holder company brand portfolio in the UK.[42] Evidence also indicates that HFSS companies have reformulated and developed new products in responses to diet-related polices in the UK, such as the Soft Drinks Industry Levy.[43,44] This evidence corresponds with pathways in the map that show how companies could redistribute advertising from regulated to unregulated products.

Pathways that illustrate the risk of food companies undermining the regulations may be particularly plausible given existing evidence has documented industry opposition to HFSS advertising regulations in the UK.[32,33] The UK government's Department for Digital, Culture, Media & Sport impact assessment of the regulations also assumed that a degree of HFSS advertising will be displaced to other media,[31] as has existing research on the TV regulation specifically.[9,45] It is also widely documented in broader literature that efforts to undermine such regulations often form part of wider market strategies that, when exerted by powerful and global corporations, are difficult to address with singular regulations.[46] Our concept map builds on this evidence by elucidating pathways through which regulation may be undermined, from which it may be possible to adapt the proposed regulations or implement additional, complementary ones to maximise the likelihood of the regulations achieving their public health goals.

Implications and further research

As the TV and online advertising regulations are not yet implemented, our findings could be used to augment the proposed legislation to encourage stakeholder reactions that maximise the regulations potential benefits. Ensuring that definitions underpinning the legislation, particularly those relating to product categories, harmonise with other legislation affecting commercial food providers may double-down the incentive to reformulate or develop new, non-HFSS products rather than market HFSS products by other means. Expanding the existing definition to a wider range of foods (e.g., salty snacks currently exempt) could have the same effect. Implementing comparable regulations on other forms of marketing, such as a ban on outdoor advertising of HFSS as has been seen in London,[47] would also limit opportunity to redistribute advertising spend for HFSS. Expediting the implementation of other regulations affecting the commercial food system, such as the proposed volume and location price promotion regulations,[48] has similar potential to maximise the benefit of the TV and online advertising ones by limiting opportunities for redistributing efforts to unregulated marketing. Some of these proposed alterations echo responses to the Department of Health and Social Care, and Department For Digital, Culture, Media and Sport 2020 policy consultation 2020 policy consultation.[49] That they were repeated and validated by experts in multiple related fields included in our study reinforce their potential benefit.

For peer review only - http://bmjopen.bmj.com/site/about/guidelines.xhtml

The concept map could be used to design a complexity-informed evaluation of the regulations. Complex explanations of intervention impacts appreciate that instead of a singular cause-effect pathway, interventions can act as stimuli that send reverberations across the system in which they reside.[50,51] Complex adaptive system methods also appreciate the role of relationships between actors contributing to a variety of processes operating at different levels and scale to produce intervention outcomes.[40] In doing so, they help avoid finding a wrong answer to important questions,[52,53] and may help measure the impact of unintended consequences alongside the outcomes that the policy sets out to achieve.[54] By explicitly exploring the connections in a complex system, these methods may also identify novel leverage points which could be targeted by future interventions. Though the map developed in our study was not explicitly conceived in systems thinking, it has many systemic qualities (e.g., emphasises the role of relationships) and correlates with other methods such as 'system mapping' that have been identified as a key component of systems-informed evaluations.[40] The concept map could be used to define focal areas for evaluative studies of both the intended and unintended consequences of the regulations or could form the basis of other systems evaluation methods.

A benefit of theory, here in the form of a concept map, is that it enables the application of findings elsewhere.[18,19] The presence of food marketing regulations in other countries[55] – albeit different to the ones proposed in the UK - suggests there may be political appetite to learn from the UK's experience. For example, policymakers could refer to the map to consider mechanisms and pathways that are particularly relevant to their country context, and thus important to consider in developing their legislation. Findings that emerge from an evaluation based on the map would also be particularly applicable in other countries and contexts, as the maps clarifies how they are embedded with other stakeholders' adaptations following the implementation of the regulations.

CONCLUSIONS

While the proposed UK TV and online food advertising regulations will be some of the most restrictive in the world, the concept map developed in this paper illustrates that the extent to which they improve diet-related health will ultimately be determined by stakeholder reactions in the surrounding system. The map may be used as a basis for establishing a comprehensive evaluation of the UK regulations, and to inform similar regulations elsewhere. To realise the full potential of the regulations, UK policymakers may also use the map to identify and prevent loopholes in the legislations before they are implemented.

FIGURE LEGEND

Figure 1 Summary of method used to develop the concept map

For peer review only - http://bmjopen.bmj.com/site/about/guidelines.xhtml

413 Figure 2 Examples of mapping concepts and pathways using Miro

414 Figure 3 Concept map of pathways through which the proposed UK TV and online advertising

415 regulations may affect the commercial food system, health and society

For peer review only

For peer review only - http://bmjopen.bmj.com/site/about/guidelines.xhtml

FUNDING

This project was funded by the National Institute for Health Research Public Health Research Programme (project number 133570). The views and opinions expressed therein are those of the authors and do not necessarily reflect those of the Public Health Research Programme, NIHR, NHS or the Department of Health. HF, MW, JA are supported by the MRC Epidemiology Unit, University of Cambridge [grant number MC/UU/00006/7].

COMPETING INTERESTS

JA and MW report research grants from the Medical Research Council, the Biotechnology and Biological Sciences Research Council, the Canadian Institutes of Health Research, the Department of Health & Social Care Policy Research Units, and National Institute for Health Research during the conduct of the study. MW is a member of the Expert Advisory Group of the Food Foundation, a Community Interest Company in the UK that is leading work on food insecurity, including coordinating an ongoing independent inquiry into childhood food insecurity, led by the All-Party Parliamentary Group on Hunger and Food Poverty. HF previously worked for a market research company, which conducts research on behalf of many companies, including those from the food and drink industry. HF, JA and MW have submitted evidence to the Department of Health and Social Care, and Department For Digital, Culture, Media and Sport 2020 consultation for the regulations under study, available here: https://www.cedar.iph.cam.ac.uk/resources/evidence-submissions/#HFSSadban. RS reports grants from the Medical Research Council, National Institute for Health Research, Wellcome Trust and Food Standards Agency during the conduct of the study. EB reports grants from Public Health England during the conduct of the study. PS reports grants from British Heart Foundation, National Institute for Health Research, Medical Research Council and Wellcome Trust during the conduct of the study. The authors declare no other conflicts of interest.

DATA AVAILABILITY STATEMENT

Each workshop map is available in **Appendix 2** but recordings are not available as it is not possible to sufficiently anonymise participants.

EXCLUSIVE LICENSE

I, the Submitting Author has the right to grant and does grant on behalf of all authors of the Work (as defined in the below author licence), an exclusive licence and/or a non-exclusive licence for contributions from authors who are: i) UK Crown employees; ii) where BMJ has agreed a CC-BY licence shall apply, and/or iii) in accordance with the terms applicable for US Federal Government officers or employees acting as part of their official duties; on a worldwide, perpetual, irrevocable,

For peer review only - http://bmjopen.bmj.com/site/about/guidelines.xhtml

royalty-free basis to BMJ Publishing Group Ltd ("BMJ") its licensees and where the relevant Journal is co-owned by BMJ to the co-owners of the Journal, to publish the Work in BMJ Open and any other BMJ products and to exploit all rights, as set out in our licence.

The Submitting Author accepts and understands that any supply made under these terms is made by BMJ to the Submitting Author unless you are acting as an employee on behalf of your employer or a postgraduate student of an affiliated institution which is paying any applicable article publishing charge ("APC") for Open Access articles. Where the Submitting Author wishes to make the Work available on an Open Access basis (and intends to pay the relevant APC), the terms of reuse of such Open Access shall be governed by a Creative Commons licence – details of these licences and which Creative Commons licence will apply to this Work are set out in our licence referred to above.

CONTRIBUTORS

EB, PS, RS, MW and JA conceived the study and acquired funding. HF and JA developed the methodology and accompanying resources and conducted the workshops. HF collated and validated the data. HF prepared the manuscript, and the draft versions were critically reviewed by EB, PS, RS, MA and JA. All authors approved the final manuscript.

For peer review only

REFERENCES

1 World Health Organization. Set of recommendations on the marketing of foods and non-alcoholic beverages to children. World Health Organization. 2010;:14.https://www.who.int/dietphysicalactivity/publications/recsmarketing/en/ (accessed 3 Nov 2017).

2 Boyland EJ, Nolan S, Kelly B, *et al.* Advertising as a cue to consume: A systematic review and meta-analysis of the effects of acute exposure to unhealthy food and nonalcoholic beverage advertising on intake in children and adults. *American Journal of Clinical Nutrition* 2016;**103**:519–33. doi:10.3945/ajcn.115.120022

3 Smith R, Kelly B, Yeatman H, *et al.* Food Marketing Influences Children's Attitudes, Preferences and Consumption: A Systematic Critical Review. *Nutrients* 2019;**11**:875. doi:10.3390/nu11040875

4 Cairns G. A critical review of evidence on the sociocultural impacts of food marketing and policy implications. *Appetite* 2019;**136**:193–207. doi:10.1016/j.appet.2019.02.002

5 American Marketing Association. Definitions of Marketing. American Marketing Association. https://www.ama.org/AboutAMA/Pages/Definition-of-Marketing.aspx (accessed 22 Oct 2017).

6 McCarthy JE. *Basic Marketing. A Managerial Approach*. Homewood, IL: : Irwin 1964.

7 The Food Foundation. The Broken Plate 2020: The state of the nations Food System. 2020.https://foodfoundation.org.uk/wp-content/uploads/2020/09/FF-Broken-Plate-2020-DIGITAL-FULL.pdf (accessed 6 Mar 2021).

8 Kelly B, King L, Chapman K, *et al.* A hierarchy of unhealthy food promotion effects: Identifying methodological approaches and knowledge gaps. American Journal of Public Health. 2015;**105**:e86–95. doi:10.2105/AJPH.2014.302476

9 Mytton O, Boyland E, Adams J, *et al.* The potential health impact of restricting less-healthy food and beverage advertising on UK television between 05.30 and 21.00 hours: a modelling study. *PLoS Medicine* 2020;**17**:e1003212. doi:10.1371/journal.pmed.1003212

10 Department of Health & Social Care. Tackling obesity: empowering adults and children to live heatlhier lives. 2020.https://www.gov.uk/government/publications/tackling-obesity%02government-strategy/tackling-obesity-empowering-adults-and-children-to-live-

For peer review only

For peer review only - http://bmjopen.bmj.com/site/about/guidelines.xhtml

healthier%02lives#empowering-everyone-with-the-right-information-to-make-healthier-choices (accessed 14 Sep 2020).

11 UK Government. Health and Care Bill. London, UK: : UK Parliament 2021. https://bills.parliament.uk/bills/3022

12 Taillie LS, Busey E, Stoltze FM, *et al.* Governmental policies to reduce unhealthy food marketing to children. *Nutrition Reviews* 2019;**0**:1–32. doi:10.1093/nutrit/nuz059

13 Ofcom. Media Nations 2020: UK report 2020. 2020. https://www.ofcom.org.uk/__data/assets/pdf_file/0010/200503/media-nations-2020-uk-report.pdf

14 Ofcom. Media nations: UK 2021. 2021.

15 Ogilvie D, Adams J, Bauman A, *et al.* Using natural experimental studies to guide public health action: turning the evidence-based medicine paradigm on its head. *Journal of Epidemiology and Community Health* 2020;**74**:203–8. doi:10.1136/jech-2019-213085

16 White M, Aguirre E, Finegood DT, *et al.* What role should the commercial food system play in promoting health through better diet? *BMJ* 2020;**368**:m545. doi:10.1136/bmj.m545

17 Mora F. Victims of the Balloon Effect: Drug Trafficking and U.S. Policy in Brazil and the Southern Cone of Latin America. *The Journal of Social, Political, and Economic Studies* 1996;**21**:115.

18 Ogilvie D, Cummins S, Petticrew M, *et al.* Assessing the Evaluability of Complex Public Health Interventions: Five Questions for Researchers, Funders, and Policymakers. *Milbank Quarterly* 2011;**89**:206–25. doi:10.1111/j.1468-0009.2011.00626.x

19 Rosas SR. Concept mapping as a technique for program theory development: An illustration using family support programs. *American Journal of Evaluation* 2005;**26**:389–401. doi:10.1177/1098214005278760

20 Burke JG, O'Campo P, Peak GL, *et al.* An introduction to concept mapping as a participatory public health research method. *Qualitative Health Research* 2005;**15**:1392–410. doi:10.1177/1049732305278876

21 Anderson LA, Slonim A. Perspectives on the strategic uses of concept mapping to address public health challenges. *Evaluation and Program Planning* 2017;**60**:194–201. doi:10.1016/j.evalprogplan.2016.08.011

For peer review only

For peer review only - http://bmjopen.bmj.com/site/about/guidelines.xhtml

22 Trochim WMK. An introduction to concept mapping for planning and evaluation. *Evaluation and Program Planning* 1989;**12**. doi:10.1016/0149-7189(89)90016-5

23 Tong A, Sainsbury P, Craig J. Consolidated criteria for reporting qualitative research (COREQ): a 32-item checklist for interviews and focus groups. *International Journal for Quality in Health Care* 2007;**19**:349–57. doi:10.1093/intqhc/mzm042

24 Albury C, Pope C, Shaw S, *et al.* Gender in the consolidated criteria for reporting qualitative research (COREQ) checklist. *International Journal for Quality in Health Care* 2021;**33**:2021. doi:10.1093/intqhc/mzab123

25 Braun V, Clarke V. To saturate or not to saturate? Questioning data saturation as a useful concept for thematic analysis and sample-size rationales. *Qualitative Research in Sport, Exercise and Health* 2019;:1–16. doi:10.1080/2159676X.2019.1704846

26 van der Graaf P, Forrest LF, Adams J, *et al.* How do public health professionals view and engage with research? A qualitative interview study and stakeholder workshop engaging public health professionals and researchers. *BMC Public Health* 2017;**17**:1–10. doi:10.1186/s12889-017-4896-1

27 Forde H, Solomon-Moore E. A Qualitative Study to Understand the Potential Efficacy of an Information-Based Sugar Reduction Intervention among Low Socioeconomic Individuals in the UK. *International Journal of Environmental Research and Public Health* 2019;**16**:413. doi:10.3390/ijerph16030413

28 Penney T, Adams J, Briggs A, *et al.* Evaluation of the impacts on health of the proposed UK industry levy on sugar sweetened beverages: developing a systems map and data platform, and collection of baseline and early impact data. 2017.

29 Chatham House. Chatham House Rule. 2021.https://www.chathamhouse.org/about-us/chatham-house-rule (accessed 18 Aug 2021).

30 Savona N, Macauley T, Aguiar A, *et al.* Identifying the views of adolescents in five European countries on the drivers of obesity using group model building. *European Journal Of Public Health* 2021;:ckaa251. doi:10.1093/eurpub/ckaa251

31 Department for Digital Culture Media and Sport. Impact assessment: introducing a 2100 to 0530 watershed on TV and online restriction for paid advertising of food and drink that are high in fat, salt and sugar (HFSS) products. London, UK: 2021.

For peer review only

For peer review only - http://bmjopen.bmj.com/site/about/guidelines.xhtml

32 Carters-White L, Chambers S, Skivington K, *et al.* Whose rights deserve protection? Framing analysis of responses to the 2016 committee of advertising practice consultation on the non-broadcast advertising of foods and soft drinks to children. *Food Policy* 2021;**104**:102139. doi:10.1016/j.foodpol.2021.102139

33 Lauber K, Hunt D, Gilmore A, *et al.* Corporate political activity in the context of unhealthy food advertising restrictions across Transport for London: A qualitative case study. *PLoS Medicine* 2021;**18**:e1003695. doi:10.1371/journal.pmed.1003695

34 Boland J, Banks S, Krabbe R, *et al.* A COVID-19-era rapid review: using Zoom and Skype for qualitative group research. *Public Health Research & Practice* 2021;:1–9. doi:10.17061/phrp31232112

35 Birt L, Scott S, Cavers D, *et al.* Member Checking: A Tool to Enhance Trustworthiness or Merely a Nod to Validation? *Qualitative Health Research* 2016;**26**:1802–11. doi:10.1177/1049732316654870

36 Tatlow-Golden M, Garde A. Digital food marketing to children: Exploitation, surveillace and rights violations. *Global Food Security* 2020;**27**:100423. doi:10.1016/j.gfs.2020.100423

37 Montgomery KC, Chester J. Interactive Food and Beverage Marketing: Targeting Adolescents in the Digital Age. Journal of Adolescent Health. 2009;**45**. doi:10.1016/j.jadohealth.2009.04.006

38 de Jans S, van de Sompel D, Hudders L, *et al.* Advertising targeting young children: an overview of 10 years of research (2006–2016). *International Journal of Advertising* 2019;**38**:173–206. doi:10.1080/02650487.2017.1411056

39 Harris JL, Frazier III W, Kumanyika SK, *et al.* Increasing disparities in unhealthy food advertising targeted to Hispanic and Black youth. 2019.

40 McGill E, Er V, Penney T, *et al.* Evaluation of public health interventions from a complex systems perspective: a research methods review. *Social Science & Medicine* 2021;**272**:113697. doi:10.1016/j.socscimed.2021.113697

41 Penney TL, Adams J, Briggs A, *et al.* Theorising how the UK soft drinks industry levy could impact population level diet and health: development of a multi-sectoral systems map. In: *International Society for Behavioural Nutrition and Physical Activity*. 2017. 203.

42 Cheek O, Froguel A, Clark M. Analysis on the potential for UK's leading food and soft drink brands to switch to marketing their non-HFSS products. London: 2021.

For peer review only - http://bmjopen.bmj.com/site/about/guidelines.xhtml

43 Pell D, Mytton O, Penney TL, *et al.* Changes in soft drinks purchased by British households associated with the UK soft drinks industry levy: controlled interrupted time series analysis. *The BMJ* 2021;**372**:n254. doi:10.1136/bmj.n254

44 Pell D, Penney T, Mytton O, *et al.* Anticipatory changes in British household purchases of soft drinks associated with the announcement of the Soft Drinks Industry Levy: a controlled interrupted time series analysis. *PLoS Medicine* 2020;**17**:e1003269. doi:10.1371/journal.pmed.1003269

45 Griffith R, Connell MO, Smith K, *et al.* The potential impacts of banning television advertising of HFSS food and drink before the watershed: Report produced for the Obesity Policy Research Unit. Institute for Fiscal Studies. 2019.https://www.ucl.ac.uk/obesity-policy-research-unit/sites/obesity-policy-research-unit/files/potentialimpactofban_new.pdf (accessed 11 Jan 2021).

46 Wood B, Williams O, Nagarajan V, *et al.* Market strategies used by processed food manufacturers to increase and consolidate their power: a systematic review and document analysis. *Globalization and health* 2021;**17**:17. doi:10.1186/s12992-021-00667-7

47 Mayor of London. Press release: Mayor confirms ban on junk food advertising on transport network. Mayor of London. 2018.https://www.london.gov.uk/press-releases/mayoral/ban-on-junk-food-advertising-on-transport-network-0 (accessed 24 Mar 2021).

48 Department of Health and Social Care. Restricting promotions of products high in fat, sugar and salt by location and by price: government response to public consultation. 2020.https://www.gov.uk/government/consultations/restricting-promotions-of-food-and-drink-that-is-high-in-fat-sugar-and-salt/outcome/restricting-promotions-of-products-high-in-fat-sugar-and-salt-by-location-and-by-price-government-response-to-public-consultati (accessed 14 Mar 2021).

49 HM Government. Introducing further advertising restrictions on TV and online for products high in fat, sugar and salt (HFSS). 2019.https://assets.publishing.service.gov.uk/government/uploads/system/uploads/attachment_data/file/795412/hfss-advertising-consultation-10-april-2019.pdf (accessed 20 Jan 2021).

50 Shiell A, Hawe P, Gold L. Complex interventions or complex systems? Implications for health economic evaluation. *BMJ* 2008;**336**:1281–3. doi:10.1136/bmj.39569.510521.AD

For peer review only

For peer review only - http://bmjopen.bmj.com/site/about/guidelines.xhtml

51 Petticrew M. When are complex interventions “complex”? When are simple interventions “simple”? *European Journal of Public Health* 2011;**21**:397–8. doi:10.1093/eurpub/ckr084

52 Rutter H, Savona N, Glonti K, *et al.* The need for a complex systems model of evidence for public health. *The Lancet* 2017;**390**:2602–4. doi:10.1016/S0140-6736(17)31267-9

53 Fink DS, Keyes KM. Wrong answers: when simple interpretations create complex problems. In: El-Sayed AM, Galea S, eds. *Systems science and population health*. New York: : Oxford University Press 2017. 25–36.

54 Roux AVD. Complex systems thinking and current impasses in health disparities research. *American Journal of Public Health* 2011;**101**:1627–34. doi:10.2105/AJPH.2011.300149

55 World Cancer Research Fund International. NOURISHING Database. https://www.wcrf.org/int/policy/nourishing-database (accessed 11 Jun 2019).

For peer review only - http://bmjopen.bmj.com/site/about/guidelines.xhtml

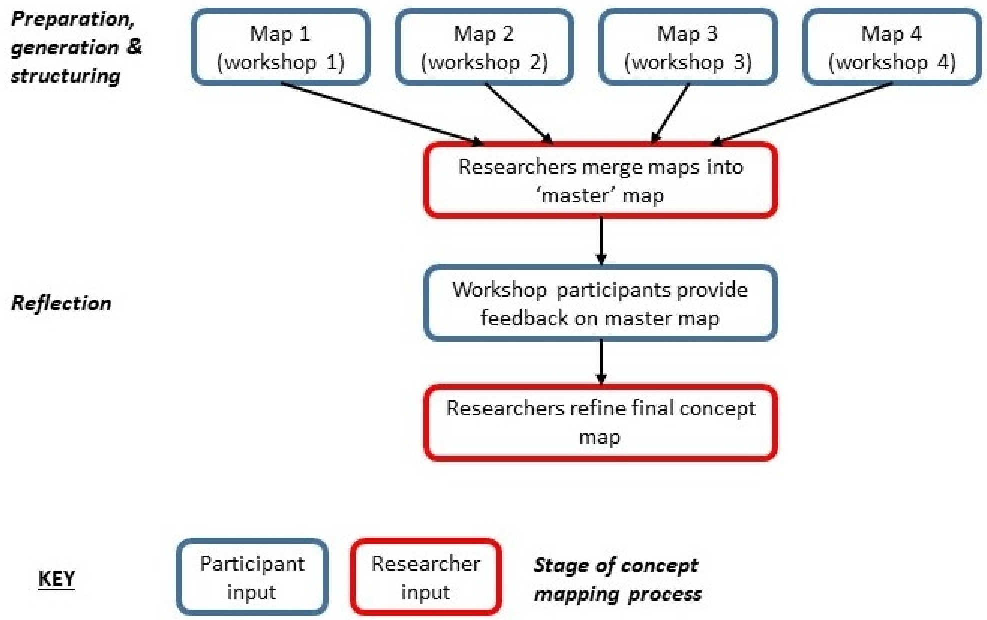

Figure 1 Summary of method used to develop the concept map

176x115mm (96 x 96 DPI)

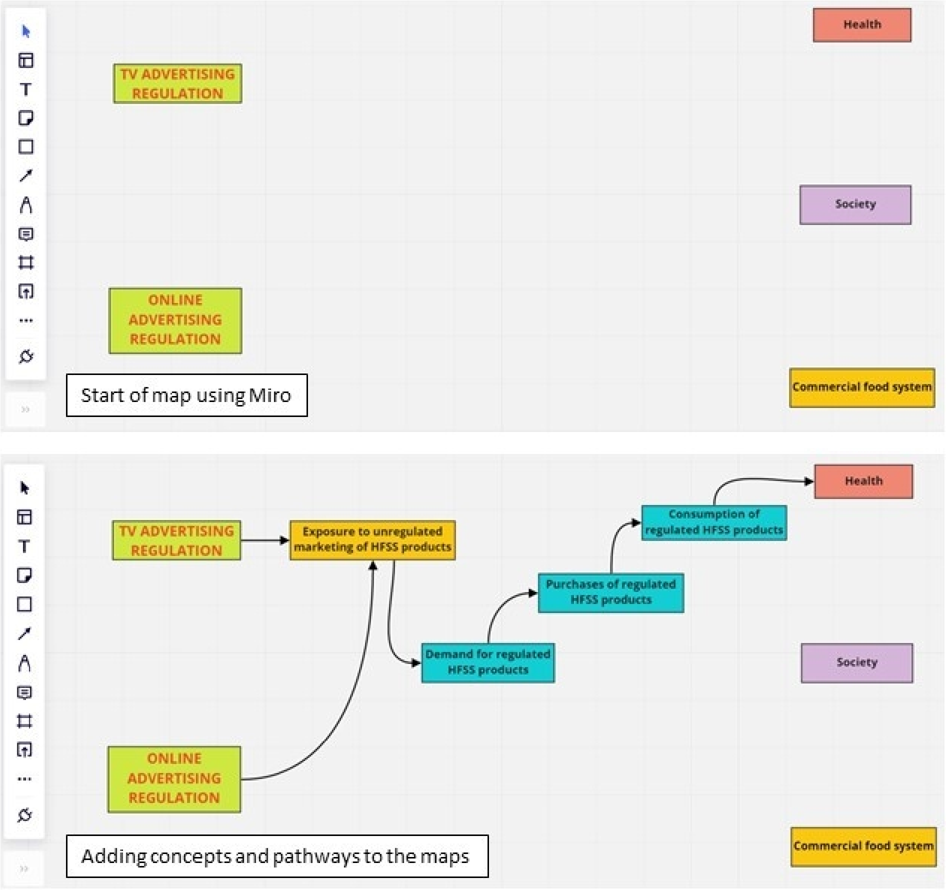

Figure 2 Examples of mapping concepts and pathways using Miro

176x158mm (96 x 96 DPI)

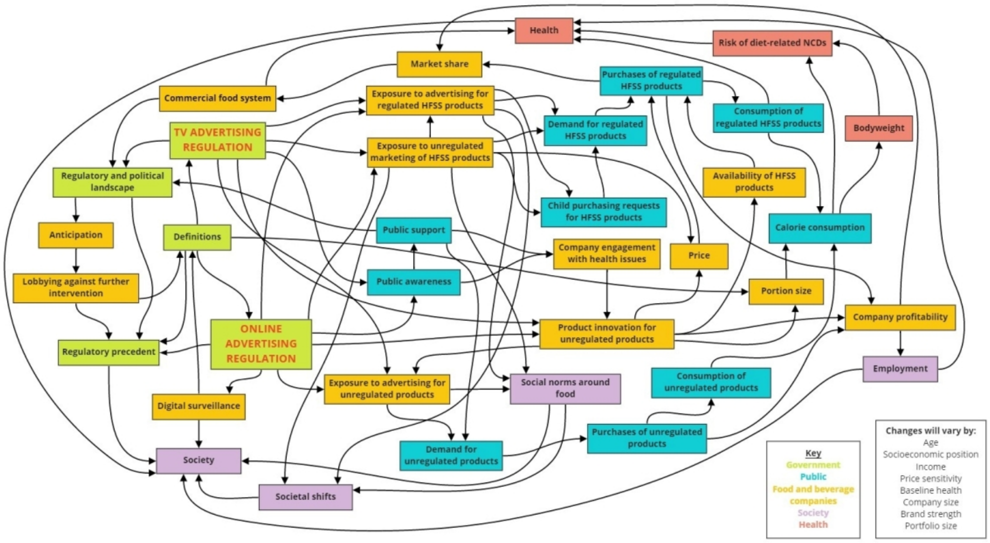

Figure 3 Concept map of pathways through which the proposed UK TV and online advertising regulations may affect the commercial food system, health

425x236mm (96 x 96 DPI)

BOX 1: Regulation details

It is expected that two new regulations will be implemented before the end of 2022:

1. A ban on advertisements for HFSS products shown on live broadcast TV from 0530-2100 ('TV advertising watershed'), including:
    a. on-demand programme services under the jurisdiction of the UK.
2. A ban on online advertisements for HFSS products, including:
    a. Non-UK regulated on-demand programme services;
    b. Social media influencers, commercial text messaging and email, all website advertising, paid-for search listings, preferential listings on price comparison sites, in-game advertisements, in-app advertising, advergames and advertorial, online display and online video.

Restrictions will not apply to 'owned media' (online property owned and controlled, usually by a brand), brand advertising, small and media enterprises (fewer than 250 employees), audio and broadcast radio, business to business (online only), or transactional content.

'HFSS' will be defined by the 2004/2005 UK Nutrient Profiling Model and within particular categories from the Sugar Reduction Strategy. Details of the regulations may change in the lead up to implementation.

Government will appoint Ofcom as the statutory regulator, who will then appoint a day-to-day regulator (expected to be ASA). [23]

For peer review only

Appendix 2: Maps 1-4 produced by each workshop

Workshop 1 Map

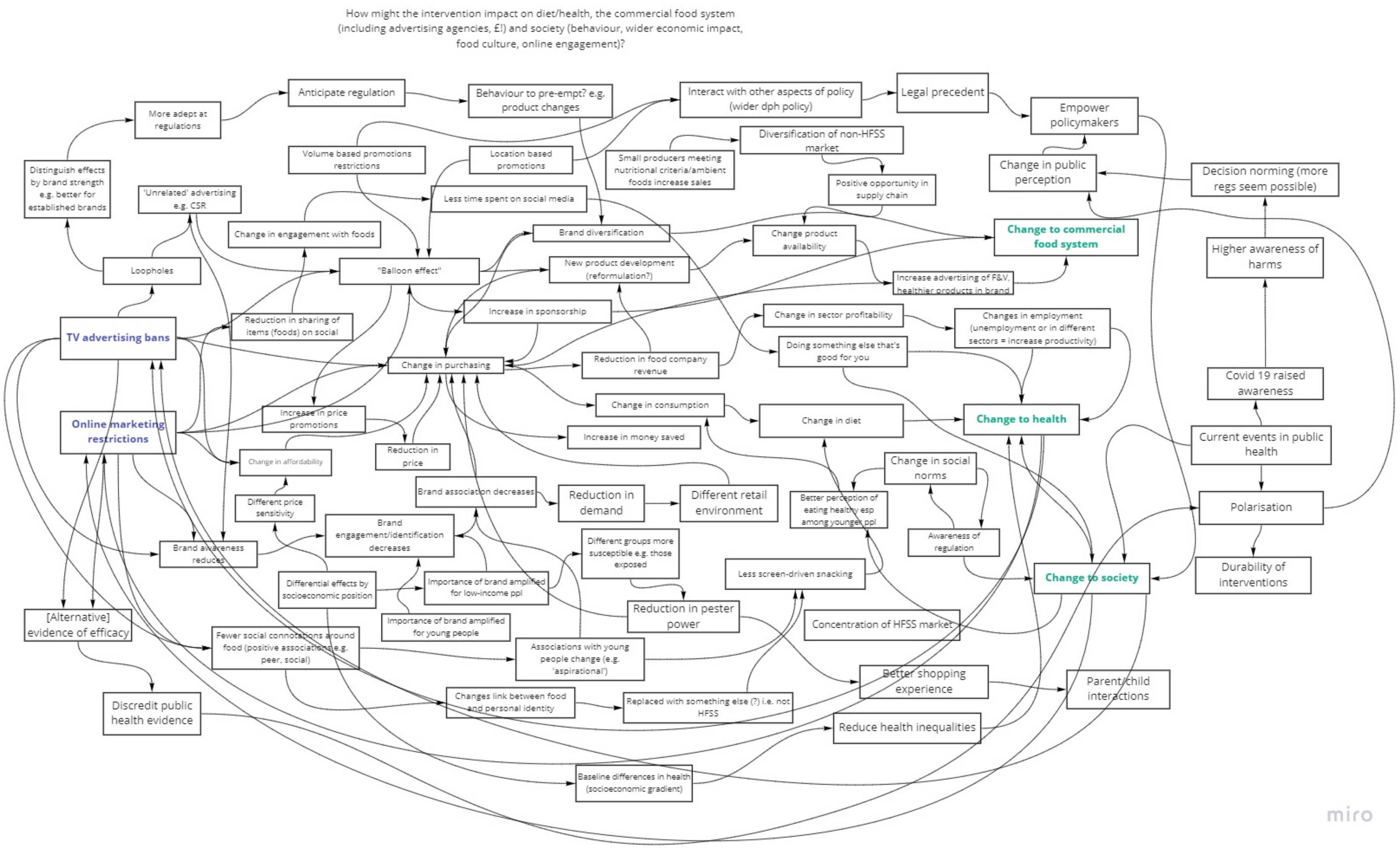

Workshop 2 Map

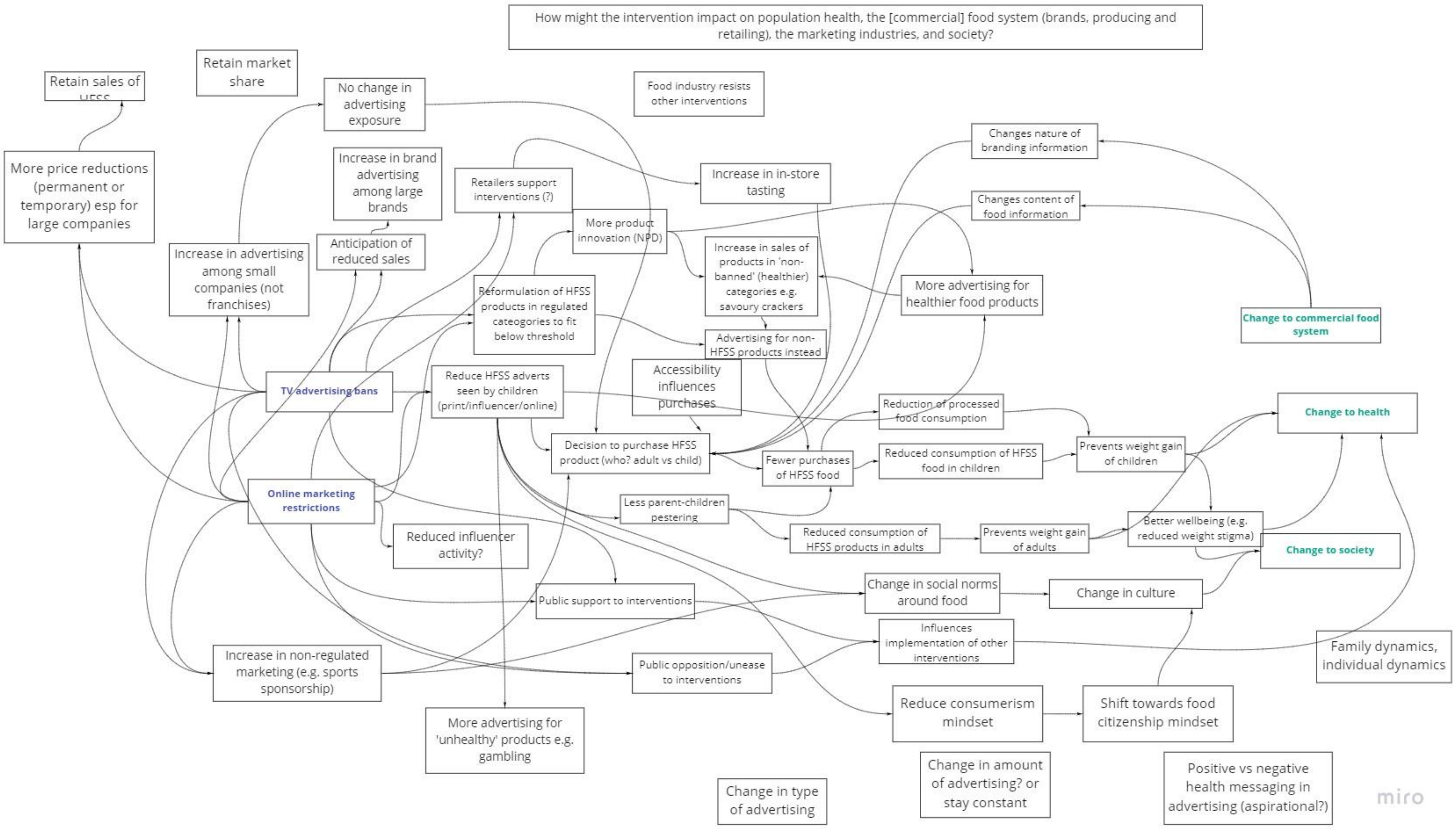

Workshop 3 Map

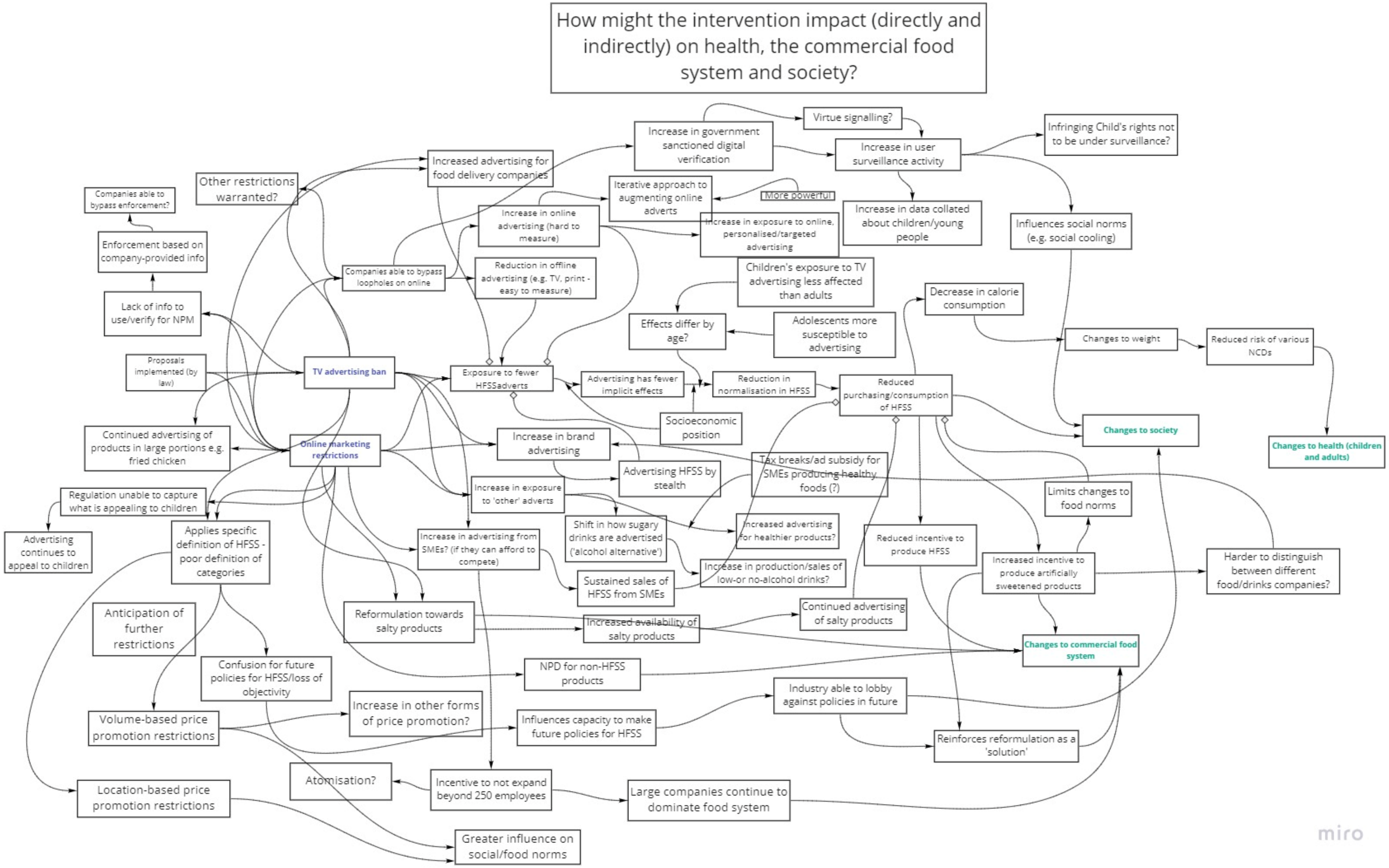

Workshop 4 Map

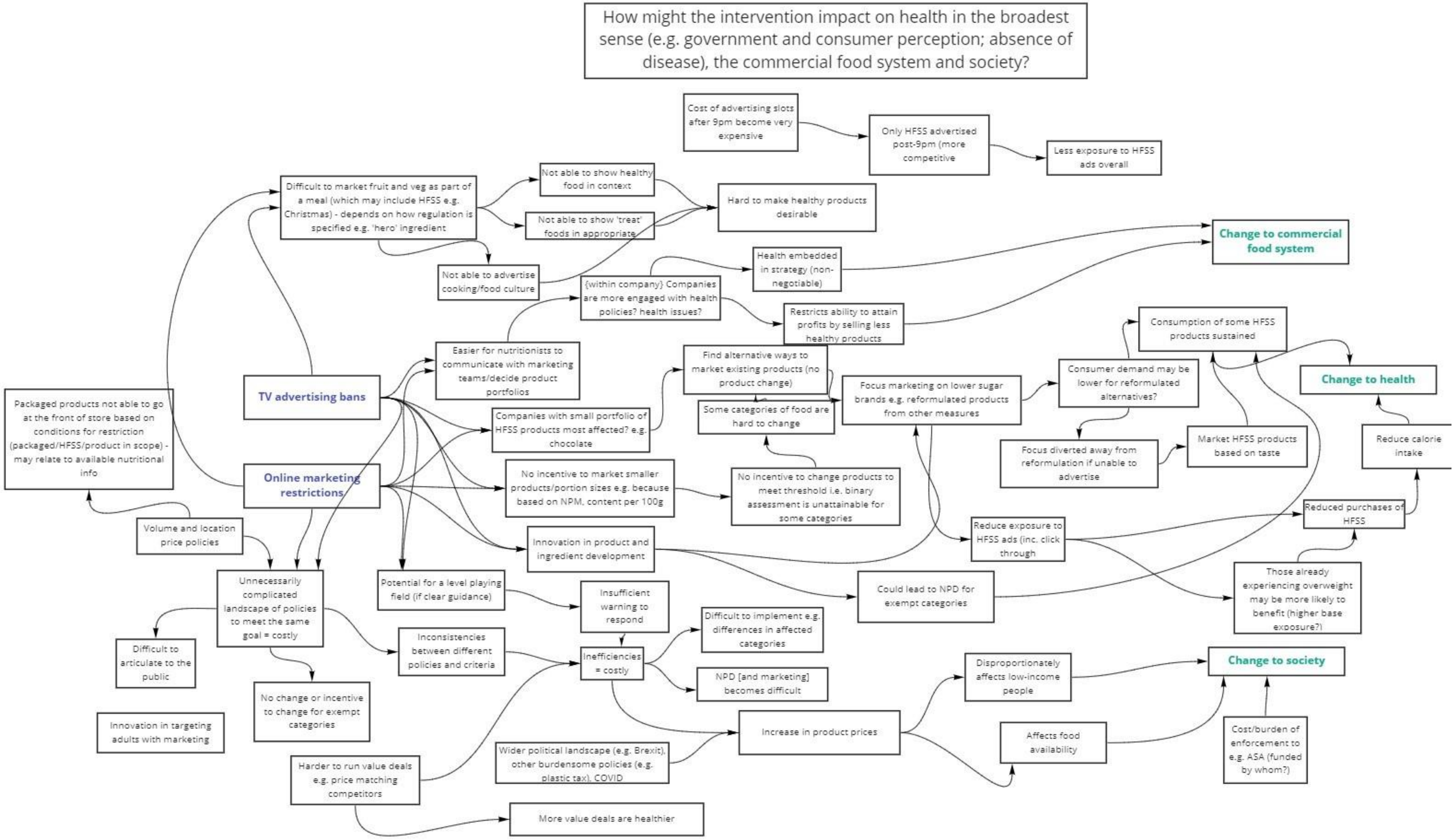

Appendix 3: Concepts from workshop maps and resulting concepts in the final map

Workshop 1 (non-industry)	Workshop 2 (non-industry)	Workshop 3 (non-industry)	Workshop 4 (industry)	Final map
More adept at regulations	Anticipation of reduced sales	Anticipating further restrictions		**Anticipation**
Behaviour to pre-empt? e.g., product changes		Proposals implemented by law		
Anticipate regulation				
Change product availability			Affects food availability	**Availability of HFSS products**
	Prevents weight gain of children	Changes to weight		**Bodyweight**
	Prevents weight gain of adults			
		Decrease in calorie consumption	Reduced calorie intake	**Calorie consumption**
Parent/child interactions	Less parent-children pestering			**Child purchasing requests for HFSS products**
Reduction in pester power				
Better shopping experience				
				**Commercial food system**
			{within company} companies are more engaged with health policies? Health issues?	**Company engagement with health issues**
			Health embedded in strategy (non-negotiable)	
			Easier for nutritionists to communicate with marketing teams	
Reduction in food company revenue			Restricts ability to attain profits by selling less healthy products	**Company profitability**

For peer review only - http://bmjopen.bmj.com/site/about/guidelines.xhtml

**Workshop 1 (non-industry)**	**Workshop 2 (non-industry)**	**Workshop 3 (non-industry)**	**Workshop 4 (industry)**	**Final map**
Change in sector profitability			Cost/burden of enforcement to e.g. ASA (funded by whom?)	
			Inefficiencies = costly	
Less screen-driven snacking	Reduced consumption of HFSS products in adults	Reduced purchasing/ consumption of HFSS	Consumption of some HFSS products sustained	**Consumption of regulated HFSS products**
Change in consumption	Reduction of processed food consumption			
Doing something else that's good for you	Reduced consumption of HFSS products in children			
Change in diet				**Consumption of unregulated products**
		Applies specific definition of HFSS - poor definitions of categories	Inconsistencies between different policies and criteria	**Definitions**
		Enforcement based on company-provided info		
		Lack of info to use/verify for NPM		
Reduction in demand				**Demand for regulated HFSS products**
Increase in money saved				
			Consumer demands may be lower for reformulated alternatives?	**Demand for unregulated products**
		Increase in user surveillance activity		**Digital surveillance**
		Infringing Child's rights not to be under surveillance?		
		Increase in surveillance activity		

For peer review only - http://bmjopen.bmj.com/site/about/guidelines.xhtml

Workshop 1 (non-industry)	Workshop 2 (non-industry)	Workshop 3 (non-industry)	Workshop 4 (industry)	Final map
		Increase in data collated about children/young people		
Changes in employment		Incentive to not expand beyond 250 employees		**Employment**
Reductions in sharing of foods on social media	No change in advertising exposure	Exposure to fewer HFSS adverts	Less exposure to ads overall	**Exposure to advertising for regulated HFSS products**
	Reduced influencer activity?	Children's exposure to TV advertising less affected than adults	Reduce exposure to HFSS ads (inc. through click through)	
	Change in amount of advertising or stay constant	Advertising has fewer implicit effects	Market HFSS products based on taste	
	Reduce HFSS adverts seen by children	More powerful	Only HFSS advertised post-9pm (more competitive)	
	Positive vs. negative health messaging in advertising (aspirational)	Virtue signalling		
	Changes content of food information	Harder to distinguish between different food/drinks companies		
		Iterative approach to augmenting online adverts		
Replaced with something else (?) i.e., not HFSS	Advertising for non-HFSS products instead	Exposure to non-HFSS adverts	Focus marketing on lower sugar brands e.g., reformulated products from other measures	**Exposure to advertising for unregulated products**
Increase advertising of F&V, healthier products in brand	More advertising for healthier food products	Increased advertising for healthier products	More value deals are healthier	
	More advertising for 'unhealthy products' e.g., gambling	Increase in exposure to 'other' adverts	Cost of advertising slots after 9pm become very expensive	
		Continued advertising of salty products		
		Increased advertising for food delivery companies		

For peer review only - http://bmjopen.bmj.com/site/about/guidelines.xhtml

Workshop 1 (non-industry)	Workshop 2 (non-industry)	Workshop 3 (non-industry)	Workshop 4 (industry)	Final map
Balloon effect	Change in type of advertising	Increase in exposure to online, personalised/targeted advertising	Difficult to market fruit and veg as part of a meal (which may include HFSS e.g., Christmas) - depends on how regulation is specified e.g. 'hero' ingredient	**Exposure to unregulated marketing of HFSS products**
Increase in sponsorship	Increase in non-regulated marketing (e.g., sports sponsorship)	Advertising continues to appeal to children	Not able to show healthy food in context	
'Unrelated' advertising e.g., CSR	Increase in in-store tasting	Companies able to bypass enforcement	Not able to show 'treat' foods in appropriate	
Loopholes	More price reductions especially for large companies	Increase in online advertising (hard to measure)	Hard to make healthy products desirable	
Location based promotions	Increase in advertising among small companies (not franchises)	Companies able to bypass loopholes online	Focus diverted away from reformulation if unable to advertise	
Increase in price promotions	Changes nature of branding information	Shift in how sugary drinks are advertised (alcohol alternatives)	Not able to advertise cooking/food culture	
Brand awareness reduces	Increase in brand advertising among large brands	Reduction in offline advertising (e.g., TV, print - easy to measure)	Find alternative ways to market existing products (no product change)	
Brand engagement/identification decreases		Continued advertising of products in large portions e.g., fried chicken	Innovation in targeting adults with marketing	
Brand association decreases		Advertising HFSS by stealth		
		Increase in other forms of price promotion?		
		Regulation unable to capture what is appealing to children		
	Better wellbeing (e.g., reduced weight stigma)			**Health**
Alternative evidence of efficacy	Food industry resists other interventions	Industry able to lobby against policies in future		**Lobbying against further interventions**

For peer review only - http://bmjopen.bmj.com/site/about/guidelines.xhtml

Workshop 1 (non-industry)	Workshop 2 (non-industry)	Workshop 3 (non-industry)	Workshop 4 (industry)	Final map
Discredit public health evidence				
Concentration of HFSS market	Retain market share	Large companies continue to dominate food system		**Market share**
		Atomisation?		
		Continued advertising of products in large portions e.g., fried chicken	No incentive to market smaller products/portion sizes e.g., because based on NPM, content per 100g	**Portion size**
Change in affordability	More price reductions especially for large companies	Increase in other forms of price promotion?	Increase in product prices	**Price**
Reduction in price			Harder to run value deals e.g., price matching competitors	
Diversification of non-HFSS markets	More product innovation (NPD)	NPD for non-HFSS products	Could lead to NPD for exempt categories	**Product innovation for unregulated products**
Positive opportunity in supply chain	Reformulation of HFSS products in regulated categories to fit below threshold	Reformulation towards salty products	Some categories of food are hard to change	
Brand diversification		Reduced incentive to produce HFSS	NPD [and marketing] becomes difficult	
New product development (reformulation?)		Increased incentive to produce artificially sweetened products	No incentive to change products to meet threshold i.e., binary assessment is unattainable for some categories	
			No change or incentive to change for exempt categories	
			Innovation in product and ingredient development	
Change in public perception				**Public awareness**
Awareness of regulation				
Higher awareness of harms				

For peer review only - http://bmjopen.bmj.com/site/about/guidelines.xhtml

Workshop 1 (non-industry)	Workshop 2 (non-industry)	Workshop 3 (non-industry)	Workshop 4 (industry)	Final map
Better perception of eating healthy especially among younger people				
	Public support for interventions		Difficult to articulate to the public	**Public support**
	Public opposition/unease to interventions			
Change in purchasing	Decision to purchases HFSS product (who? Adult vs child)	Reduced purchasing/consumption of HFSS	Reduced purchases of HFSS	**Purchases of HFSS regulated products**
	Fewer purchases of HFSS food			
	Retain sales of HFSS			
Chang e in purchasing	Increase in sales of products in 'non-banned' healthier categories e.g., savoury crackers	Increased availability of salty products		**Purchases of unregulated products**
		Increase in production/sales of low or no alcoholic drinks		
Durability of interventions	Retailers support interventions	Volume-based price promotion restrictions	Wider political landscape (e.g., Brexit) other burdensome policies (e.g., plastic tax, covid)	**Regulatory and political landscape**
Interact with other aspects of DPH policy		Location-based price promotion restrictions	Volume and location price policies	
Location based promotions		Other restrictions warranted	Unnecessarily complicated landscape of policies to meet the same goal = costly	
Covid-19 raised awareness		Reinforces reformulation as 'solution'	Potential for a level playing field (if clear guidance)	
Current events in public health		Tax breaks/ad subsidy for SMEs producing healthy foods (?)	Insufficient warning to respond	
Volume based promotions restrictions			Difficult to implement e.g., differences in affected categories	
			Packaged products not able to go at the front of store based on conditions for restrictions (packaged/HFSS/product in	

For peer review only - http://bmjopen.bmj.com/site/about/guidelines.xhtml

Workshop 1 (non-industry)	Workshop 2 (non-industry)	Workshop 3 (non-industry)	Workshop 4 (industry)	Final map
			scope) - may relate to available nutritional info)	
Legal precedent	Influences implementation of other interventions	Influences capacity to make future policies for HFSS		**Regulatory precedent**
Decision norming (more regs seem possible)		Confusion for future policies for HFSS/loss of objectivity		
Empower policymakers		Increase in government sanctioned digital verification		
		Reduced risk of various NCDs		**Risk of diet-related NCDs**
Fewer social connotations around food	Change in social norms around food	Influences social norms e.g., social cooling		**Social norms around food**
Changes in social norms	Family dynamics, individual dynamics	Reduction in normalisation of HFSS		
Change in engagement with foods		Limits changes to food norms		
Associations with young people change (e.g., aspirational)		Greater influence on social/food norms		
				**Society**
Changes link between food and personal identity	Shift towards food citizenship mindset			**Societal shifts**
Different retail environment	Reduce consumerism mindset			
Less time spent on social media	Change in culture			
Polarisation				
Small producers meeting nutritional criteria/ambient food increase sales	Accessibility influences purchases	Increase in advertising from SMEs? If they can afford to compete	Companies with small portfolio of HFSS products most affected? e.g., chocolate	**Changes vary by…**
Different price sensitivity	Increase in advertising among small companies (not franchises)	Sustained sales of HFSS from SMEs	Those already experiencing overweight may be more likely to benefit (higher base exposure?)	
Importance of brand amplified for low-income people		Effects differ by age?	Disproportionately affects low-income people	

**Workshop 1 (non-industry)**	**Workshop 2 (non-industry)**	**Workshop 3 (non-industry)**	**Workshop 4 (industry)**	**Final map**
Importance of brand amplified for young people		Socioeconomic position		
Different groups more susceptible e.g., those exposed		Adolescents more susceptible to advertising		
Differential effects by socioeconomic position				
Baseline differences in health (socioeconomic gradient				
Reduce health inequalities				
Distinguish effects by brand strength e.g., better for established brands				

Notes: HFSS: high fat, salt and sugar; NCD: non-communicable disease; NPD: new product development; NPM: Nutrient Profile Model; SME: small and medium enterprises.

For peer review only

For peer review only - http://bmjopen.bmj.com/site/about/guidelines.xhtml

Appendix 1: Consolidated criteria for reporting qualitative studies (COREQ): 32-item checklist

No.	Item	Guide questions/description	Page #
**Domain 1: Research team and reflexivity**			
Personal characteristics			
1.	Interviewer/facilitator	Which author/s conducted the interviews or focus groups?	6
2.	Credentials	What were the researcher's credentials? e.g., PhD, MD	Title page
3.	Occupation	What was their occupation at the time of the study?	Title page
4.	Gender	Was the researcher male or female?	See note
5.	Experience and training	What experience or training did the researcher have?	6
Relationship with participants			
6.	Relationship established	Was a relationship established prior to study commencement?	6
7.	Participant knowledge of the interviewer	What did the participants know about the research? e.g., personal goals, reasons for doing the research	6
8.	Interviewer characteristics	What characteristics were reported about the interviewer/facilitator? e.g., bias, assumptions, reasons and interests in the research topic	N/A
**Domain 2: study design**			
Theoretical framework			
9.	Methodological orientation and Theory	What methodological orientation was stated to underpin the study? e.g., grounded theory, discourse analysis, ethnography, phenomenology, content analysis	6
Participant selection			
10.	Sampling	How were the participants selected? e.g., purposive, convenience, consecutive, snowball	6
11.	Method of approach	How were participants approached? e.g., face-to-face, telephone, mail, email	6
12.	Sample size	How many participants were in the study?	8
13.	Non-participation	How many people refused to participate or dropped out? Reasons?	N/A
Setting			
14.	Setting of data collection	Where was the data collected? e.g., home, clinic, workplace	6-7
15.	Presence of non-participants	Was anyone else present besides the participants and researchers?	N/A
16.	Description of sample	What are the important characteristics in the sample? e.g., demographic data, date	8
Data collection			
17.	Interview guide	Were questions, prompts, guides provided by the authors? Was it pilot tested?	6
18.	Repeat interviews	Were repeat interviews carried out? If yes, how many?	N/A
19.	Audio/visual recording	Did the research use audio or visual recording to collect the data?	7
20.	Field notes	Were field notes made during and/or after the interview or focus group?	7
21.	Duration	What was the duration of the interviews or focus group?	6
22.	Data saturation	Was data saturation discussed?	6
23.	Transcripts returned	Were transcripts returned to participants for comment and/or correction?	8

For peer review only - http://bmjopen.bmj.com/site/about/guidelines.xhtml

No.	Item	Guide questions/description	Page #
**Domain 3: analysis and findings**			
Data analysis			
24.	Number of data coders	How many data coders coded the data?	N/A
25.	Description of the coding tree	Did authors provide a description of the coding tree?	N/A
26.	Derivation of themes	Were themes identified in advance or derived from the data?	N/A
27.	Software	What software, if applicable, was used to manage the data?	6-7
28.	Participant checking	Did participants provide feedback on the findings?	8
Reporting			
29.	Quotations presented	Were participant quotations presented to illustrate the themes/findings? Was each quotation identified? e.g., participant number	N/A
30.	Data and findings consistent	Was there consistency between the data presented and the findings?	9
31.	Clarity of major themes	Were major themes clearly presented in the findings?	N/A
32.	Clarity of minor themes	Is there a description of diverse cases or discussion of minor themes?	N/A

Taken from: Tong A, Sainsbury P, Craig J. Consolidated criteria for reporting qualitative research (COREQ): a 32-item checklist for interviews and focus groups. *Int J Qual Health Care*. 2007;19(6):349-357. doi:10.1093/intqhc/mzm042

Question on gender omitted in response to recent update: Albury C, Pope C, Shaw S, et al. Gender in the consolidated criteria for reporting qualitative research (COREQ) checklist. *International Journal for Quality in Health Care*. 2021;33(4):2021. doi:10.1093/intqhc/mzab12

For peer review only

BMJ Open

Exploring the potential impact of the proposed UK TV and online food advertising regulations: a concept mapping study

Journal:	*BMJ Open*
Manuscript ID	bmjopen-2021-060302.R1
Article Type:	Original research
Date Submitted by the Author:	05-Apr-2022
Complete List of Authors:	Forde, Hannah; MRC Epidemiology Unit<br>Boyland, Emma; University of Liverpool Institute of Population Health, Department of Psychology<br>Scarborough, Peter; University of Oxford, Nuffield Department of Population Health; NIHR Oxford Biomedical Research Centre<br>Smith, Richard ; University of Exeter Medical School, Institute of Health Research<br>White, Martin; MRC Epidemiology Unit<br>Adams, J; MRC Epidemiology Unit
**Primary Subject Heading**:	Public health
Secondary Subject Heading:	Global health, Health policy, Qualitative research
Keywords:	PUBLIC HEALTH, NUTRITION & DIETETICS, QUALITATIVE RESEARCH

SCHOLARONE™
Manuscripts

I, the Submitting Author has the right to grant and does grant on behalf of all authors of the Work (as defined in the below author licence), an exclusive licence and/or a non-exclusive licence for contributions from authors who are: i) UK Crown employees; ii) where BMJ has agreed a CC-BY licence shall apply, and/or iii) in accordance with the terms applicable for US Federal Government officers or employees acting as part of their official duties; on a worldwide, perpetual, irrevocable, royalty-free basis to BMJ Publishing Group Ltd ("BMJ") its licensees and where the relevant Journal is co-owned by BMJ to the co-owners of the Journal, to publish the Work in this journal and any other BMJ products and to exploit all rights, as set out in our licence.

The Submitting Author accepts and understands that any supply made under these terms is made by BMJ to the Submitting Author unless you are acting as an employee on behalf of your employer or a postgraduate student of an affiliated institution which is paying any applicable article publishing charge ("APC") for Open Access articles. Where the Submitting Author wishes to make the Work available on an Open Access basis (and intends to pay the relevant APC), the terms of reuse of such Open Access shall be governed by a Creative Commons licence – details of these licences and which Creative Commons licence will apply to this Work are set out in our licence referred to above.

Other than as permitted in any relevant BMJ Author's Self Archiving Policies, I confirm this Work has not been accepted for publication elsewhere, is not being considered for publication elsewhere and does not duplicate material already published. I confirm all authors consent to publication of this Work and authorise the granting of this licence.

For peer review only

Exploring the potential impact of the proposed UK TV and online food advertising regulations: a concept mapping study

Hannah Forde[a*], Emma Boyland[b], Peter Scarborough[c,d], Richard Smith[e], Martin White[a], Jean Adams[a]

[a] MRC Epidemiology Unit, University of Cambridge School of Clinical Medicine, Box 285 Institute of Metabolic Science, Cambridge Biomedical Campus, Cambridge, CB2 0QQ

[b] Department of Psychology, Institute of Population Health, University of Liverpool, Eleanor Rathbone Building, Bedford Street South, Liverpool, L69 7ZA

[c] Nuffield Department of Population Health, University of Oxford, Old Road Campus, Roosevelt Drive, Headington, Oxford, OX3 7LF

[d] NIHR Oxford Biomedical Research Centre, The Joint Research Office, Second Floor, OUH Cowley Unipart House Business Centre, Garsington Road, Oxford, OX4 2PG

[e] Medical School Building, College of Medicine and Health, University of Exeter, St Luke's Campus, Heavitree Road, Exeter, EX1 2LU

*Correspondence to Hannah Forde: hf332@medschl.cam.ac.uk

MAIN MANUSCRIPT WORD COUNT: 5,555

For peer review only

For peer review only - http://bmjopen.bmj.com/site/about/guidelines.xhtml

ABSTRACT

Objectives In July 2020 the UK Government announced an intention to restrict advertisements for products high in fat, salt or sugar on live broadcast, catch-up and on-demand television before 9pm; and paid for online advertising. As no other jurisdiction has implemented similar regulations, there is no empirical evidence about how they might perturb the food system. To guide the regulations' implementation and evaluation, we aimed to develop a concept map to hypothesise their potential consequences for the commercial food system, health and society.

Methods We used adapted group concept mapping in four virtual workshops with food marketing and regulation experts across academia, civil society, government organisations, and industry (N=14), supported by Miro software. We merged concepts derived from the four workshops to develop a master map and then invited feedback from participants via email to generate a final concept map.

Results The concept map shows how the reactions of stakeholders to the regulations may reinforce or undermine the impact on the commercial food system, health and society. The map shows adaptations made by stakeholders that could reinforce, or undermine, positive impacts on public health. It also illustrates potential weaknesses in the design and implementation of the regulations that could result in little substantial difference to public health.

Conclusions

Prior to the regulations' initial implementation or subsequent iterations, they could be altered to maximise the potential for reinforcing adaptations, minimise the potential for undermining adaptations, and ensure they cover a wide range of advertising opportunities and foods. The concept map will also inform the design of an evaluation of the regulations and could be used to inform the design and evaluation of similar regulations elsewhere.

Strengths and limitations of this study

- By including a diverse range of experts, we developed the first comprehensive articulation of the potential pathways through which new advertising regulations may impact on the commercial food system, health and society.
- Holding the workshops online may have facilitated greater attendance, particularly as we employed techniques to minimise the limitations of online data collection.
- Timing the workshops after sufficient details were known about the regulations allowed for a meaningful discussion about their impact but with enough time for the study's findings to feed into the regulations' design.

For peer review only - http://bmjopen.bmj.com/site/about/guidelines.xhtml

- Though we did not aim to achieve saturation in this study, we found it difficult to recruit participants from industry.
- We necessarily invited more individuals than those who ultimately participated, which may affect the transferability of the study's findings.

For peer review only

For peer review only - http://bmjopen.bmj.com/site/about/guidelines.xhtml

INTRODUCTION

The World Health Organization (WHO) recommends that member states limit children's exposure to marketing for less healthy foods.[1] The recommendation reflects evidence that marketing influences food preferences and consumption, both at an individual (micro-level impacts)[2,3] and societal level (macro-level impacts).[4] Marketing has been defined as "the activity, set of institutions, and processes for creating, communicating, delivering, and exchanging offerings that have value for customers, clients, partners, and society at large".[5] Marketing is exerted through a range of activities, including those related to the product, its place, price and promotion.[6] Promotion includes building games around products (advergames), social media 'influencers', and paid for advertising in any medium. Products high in fat, salt, or sugar (HFSS) are disproportionately advertised in the UK, with only 2.5% of total food and soft drink advertising spend going towards fruit and vegetables in 2020.[7] Though the causal pathways between advertising and obesity are likely to be complex,[8] it is estimated that 6.4% (95% confidence interval (CI): 2.0-13.8) of UK childhood obesity and 5.0% (95% CI: 1.5-10.9) of overweight is attributable to HFSS television advertising alone.[9]

To address concerns about the prevalence of childhood obesity, in July 2020 the United Kingdom's (UK) Government Department of Health and Social Care published an intention to restrict advertisements for HFSS food and drink products on live broadcast, catch-up and on-demand television ('TV') before 9pm and paid for online advertising ('online').[10] Current details of these proposed regulations are summarised in **Box 1**, and though they have passed through the House of Lords in the Health and Care Bill,[11] details of the regulations may change before they receive Royal Assent and are implemented. Although these regulations are likely to impact on both TV and online advertising content that adults see, they have been consistently framed in policy documents as focusing on tackling childhood obesity. The first government document they were proposed in was a Childhood Obesity Strategy,[12] and subsequent strategies and policy documents have repeatedly referred to them in the context of childhood obesity.[10,13] Further, the design of the TV aspect (banning HFSS adverts from 0530-2100) reflects hours when children are most likely to be watching.

For peer review only - http://bmjopen.bmj.com/site/about/guidelines.xhtml

BOX 1: Regulation details

It is expected that two new regulations will be implemented before the end of 2022:

1. *A ban on advertisements for HFSS products shown on live broadcast TV from 0530-2100 ('TV advertising watershed'), including:*
   a. *on-demand programme services under the jurisdiction of the UK.*
2. *A ban on online advertisements for HFSS products, including:*
   a. *Non-UK regulated on-demand programme services;*
   b. *Social media influencers, commercial text messaging and email, all website advertising, paid-for search listings, preferential listings on price comparison sites, in-game advertisements, in-app advertising, advergames and advertorial, online display and online video.*

Restrictions will not apply to 'owned media' (online property owned and controlled, usually by a brand), brand advertising, small and medium enterprises (fewer than 250 employees), audio and broadcast radio, business to business (online only), or transactional content.

'HFSS' will be defined by the 2004/2005 UK Nutrient Profiling Model and within particular categories from the Sugar Reduction Strategy. Details of the regulations may change in the lead up to implementation.

Government will appoint Ofcom as the statutory regulator, who will then appoint a day-to-day regulator (expected to be ASA).[13]

The TV and online regulations proposed for the UK will be some of the most restrictive worldwide, and the first to explicitly address paid for online advertising.[14] Overall, 18% of UK advertising spend is for TV slots and at least 63% for online slots.[15] Though there has been a recent decline in broadcast TV viewing in the UK, average viewing time remains around three hours per day for ages 4 years and above.[15] The Covid-19 pandemic has accelerated use of subscription video-on-demand services, with viewing of services such as Netflix and Amazon Prime Video almost doubling in 2020 to an estimated 1 hour per person per day.[16] Such services would be covered by the proposed online regulation rather than the TV one. While the decline in broadcast TV viewing has been more pronounced among younger people (for 16-24 year olds down 18%, and for children 4-15 year olds down 16% in 2019),[15] this has corresponded with an increase in viewing of subscription video-on-demand services among younger people (by 55 minutes to an average of two hours per day between April 2019 and April 2020).[16] It has been estimated that a pre-9pm ban on HFSS TV food advertising would result in a 4.6 (1.4-9.5)% reduction in childhood obesity and a 3.6 (1.1-7.4)% reduction in childhood overweight prevalence.[9] Effects were two-fold greater in the least compared to the most affluent social groups and would likely be amplified by comparable restrictions on online food

For peer review only - http://bmjopen.bmj.com/site/about/guidelines.xhtml

promotion.[9] The ultimate results of such a regulation were predicted to depend on how HFSS advertising patterns change in response.[9] Though less is known about the potential effects of an online ban, emerging evidence indicates that online marketing techniques (e.g., use of social media influencers) may be particularly pervasive and persuasive.[17–19]

Few evaluations of such food advertising restrictions have been conducted worldwide,[14] partly because there have been few comparable regulations. There are also challenges to evaluating this type of intervention that is delivered to whole populations and so is impractical to subject to experimental evaluation techniques such as randomised controlled trials.[20] Furthermore, the commercial food sector exhibits characteristics of a complex adaptive system.[21] Adaptations made by stakeholders residing in the system that is regulated may lead to both intended and unintended consequences that ultimately impact on the overall effectiveness of regulations.[21] The 'balloon effect' proposes that restrictions on one type of marketing can lead to increases in others,[22] as companies and other aspects of the food system adapt. Articulating these possible adaptations and their potential consequences should help refine details of the regulations before implementation. Understanding possible adaptations and consequences should also help inform the design of any evaluation.

Some other countries are following a similar path of legislation in this realm – though more often through industry self-regulation [23–27] – emphasising the need to develop generalisable evidence about the impact of the UK regulations. To maximise the applicability of evaluation findings to policymakers outside of the UK, it is helpful for evaluators to test theories as well as evaluate interventions.[28] Theory-driven evaluation first requires the development and clear articulation of program theory.[29] Concept mapping is an approach particularly useful for public health researchers interested in developing theory.[30] A concept map is a "diagram of proposed relationships among a set of concepts….about a particular question….or topic".[31] Concept maps can be used to help organise ideas, demarcate an area of interest and plan evaluations. Group concept mapping is a structured approach involving group work that is flexible to many public health contexts.[32]

Objectives

In this study, we used an approach inspired by group concept mapping to develop a concept map of how the new TV and online advertising regulations may impact on the commercial food system, health and society. We aimed to describe how the regulations may interact with the food system so that evaluations of the regulations can be grounded in clearly articulated theory, and so that adaptations to the regulations that could improve the health impact can be identified before implementation.

For peer review only - http://bmjopen.bmj.com/site/about/guidelines.xhtml

METHODS

Study design

We created a concept map of the potential pathways through which the regulations may impact on the commercial food system, health and society. By 'food system' we mean the interdependent network of entities involved in agriculture and fisheries, food processing and production, storage and distribution, wholesaling and retailing, and preparation and marketing of raw, processed and ready to eat foods.[21] By 'society', we mean the wider social system in which the food system is embedded. We developed the map using an adapted version of a group concept mapping method in four workshops.[32] The study reporting adheres to the Consolidated Criteria for Reporting Qualitative Research (COREQ) (**Appendix 1**),[33] but recognises proposed amendments relating to gender.[34]

Participant recruitment

Workshop participants were recruited from academia, civil society, government organisations and industry (e.g., food industry, media, advertising). Individuals were eligible for inclusion if they had professional knowledge and experience of food marketing regulation within their sector and were based in the UK. We identified individuals from our existing contacts in these sectors and by searching the websites of relevant organisations. In total, 63 individuals were invited by email to take part in the study (8 from academia, 15 from civil society, 11 from government organisations, and 29 from industry). We aimed to recruit up to 20 individuals, approximately evenly distributed across the participant groups. As we were not aiming to reach 'saturation',[35] we decided on the number of people to recruit to the study pragmatically, based on the resources available to us but allowing for sufficient breadth.

Participants from industry attended a separate workshop to those from academia, civil society and government organisations due to the potential for conflicts of interests between sectors. We set a limit of 10 participants per workshop in addition to the facilitators (JA and HF, who both had qualitative research experience, e.g., [36,37]), which is considered a manageable total number of participants to permit dialogue and engagement.[32] Workshops were arranged around participants' availability in July and August 2021 and lasted 2 hours each.

Data collection

Building on previous work that has used group concept mapping to inform the design of evaluations of population health interventions,[38] we used the first three steps of group concept mapping (preparation, generation and structuring)[32] and added a fourth (reflection). The first three steps were achieved in the workshops, and the final step was achieved using an online feedback form. We

For peer review only - http://bmjopen.bmj.com/site/about/guidelines.xhtml

held the workshops on Zoom, an online videoconferencing software (https://zoom.us/) to minimise time demands on participants and as data collection took place during COVID-19 restrictions. In the workshops, we used a combination of pre-piloted Microsoft PowerPoint slides and Miro software (https://miro.com/) to provide instructions to participants and visualise their contributions as they were made, respectively. Our data consisted of screenshots of maps as they developed, the map from each workshop, audio recordings of the workshops, and post-workshop feedback returned through an online form. Workshops were held under the Chatham House Rule[39]: participants were told they could use the information discussed in the workshops, but they could not reveal the identity or affiliation of other participants. **Figure 1** summarises the method used to develop the final concept map.

INSERT FIGURE 1 HERE

Preparation

Preparation entailed setting out the aims and processes of the workshop and agreeing the focus area of the map.[32] At the beginning of each workshop, the workshop facilitators introduced the aims and processes. They reminded participants of the intervention details, the withdrawal process and that the workshops were being recorded. The facilitators proposed that the focus area was "what are the potential pathways through which the intervention might impact on health, the commercial food system and society?". Participants were invited to help refine this during a discussion of approximately 5 minutes.

Generation

Generation is a divergent process where participants individually brainstorm a long list of responses to the focus area and consider the relative importance of each response.[32] Participants were given around 10 minutes to independently generate a list of as many responses as possible to the refined focus area, including pathways to both positive and negative impacts arising from the regulations.

Structuring

Structuring is a convergent process where participants organise and critically reflect on ideas and relationships between concepts.[32] For approximately 60 minutes, participants were asked in turn to contribute responses to the focus area from their individual brainstorming in order of relative importance. These were structured and visualised in real-time using Miro, which was shared on-screen with participants, with new concepts and relationships added to a draft map as participants suggested them (see **Figure 2**). Once all responses were included, participants were invited to reflect on the map, adding additional concepts and relationships as required. We adopted an inclusive approach to adding

For peer review only - http://bmjopen.bmj.com/site/about/guidelines.xhtml

concepts and relationships to maps, including everything mentioned and not deleting anything previously added.

INSERT FIGURE 2 HERE

Reflection

After the workshops, we merged the map from each workshop into one 'master' map. We used a method inspired by those employed in other mapping projects.[40] First, HF charted all concepts in the maps into a Microsoft Excel sheet, and similar or identical concepts across the maps were grouped and refined into simplified concepts and accompanying descriptions. Second, these refined concepts were mapped in a way that corresponded with pathways depicted in the four separate maps. Concepts not immediately fitting anywhere were placed to the side for further deliberation with JA. As we took an inclusive approach, all concepts from the individual maps contributed to the master map. The master map was discussed with the wider research team (EB, PS, MW, RS) and steering committee, prompting some minor changes but notably, no areas of significant disagreement.

We then circulated the master map to all workshop participants by email. The email contained a link to an online form issued via REDCap (https://www.project-redcap.org/) that asked questions about the map to seek suggested changes. We used the suggestions to produce a final concept map.

Analysis

Beyond merging the maps from each workshop into a master map, no formal analyses were conducted.

Ethics

The study received favourable review from the University of Cambridge School of Humanities and Social Science Research Ethics Committee in June 2021, reference number 21.276. Participants were provided with an information sheet about the study and provided informed consent before joining a workshop using an e-consent form issued via REDCap.

Patient and public involvement

Patients and/or the public were not involved in the design, conduct, reporting or dissemination plans of this research.

For peer review only - http://bmjopen.bmj.com/site/about/guidelines.xhtml

215 **RESULTS**

216 From four workshops with a total of 14 participants, we developed a concept map to describe how the proposed TV and online advertising regulations may impact on the commercial food system, health and society. Here we present the concept map and describe its component concepts.

Participant characteristics

We held four workshops: one with individuals from industry, and three with individuals from academia, civil society, and government organisations (see **Table 1**). As the focus was on generating the map as a group, we did not collate any demographic information about participants.[40]

Table 1. Sectors included in each workshop

Participant sectors per workshop	Workshop 1	Workshop 2	Workshop 3	Workshop 4	Total
Academia	2	1	1	0	4
Civil society	2	1	3	0	6
Government organisation	0	1	1	0	2
Industry	0	0	0	2	2
				**Grand total**	14

Concept map of anticipated adaptations to the regulations

The maps produced in each workshop are provided in **Appendix 2**, and they illustrate the nuance in focus between workshops. For example, the workshop with industry participants focused more on the technical difficulties presented by the regulations than in other workshops. Six workshop participants provided feedback on the master map during the reflection stage (academia = 2, civil society = 3, government organisation = 1). In response to the feedback, we refined some of the connections between concepts (e.g., adding a direct link connecting health and employment), and highlighted the regulations to make them more visibly striking.

The resultant concept map is presented in **Figure 3,** and it depicts the possible pathways of change that could follow the regulations**.** Colour coding is used to differentiate the groups of reactions to the regulations: government, food and beverage companies, public, society and health. Pathways depicted are not exhaustive, as it is possible that other links between concepts exist that were not captured in the workshops. The map is also accompanied by a list of factors that may modify the impact of pathways that it depicts, such as socioeconomic position and company size. The concepts contained in each workshop map, and the corresponding concepts they were assigned in the final concept map, are provided in **Appendix 3**. Concepts are described in more detail in **Table 2**.

For peer review only - http://bmjopen.bmj.com/site/about/guidelines.xhtml

INSERT FIGURE 3 HERE

For peer review only

For peer review only - http://bmjopen.bmj.com/site/about/guidelines.xhtml

243 **Table 2. Description of concepts in the concept map**

Statement	Description
**Anticipation**	Food and drink companies foresee the introduction of the regulations[a], and possibly other related legislation e.g., volume and location price promotion.
**Availability of HFSS products**	Availability of all HFSS foods and beverages, both within and outside the scope of the regulations[a], in physical and online shops.
**Bodyweight**	In terms of Body Mass Index (BMI), overweight or obesity status.
**Calorie consumption**	Total energy intake of individuals.
**Child purchasing requests for HFSS products**	Degree to which children make purchasing requests to caregivers for all HFSS products, both within and outside the scope of the regulations[a].
**Commercial food system**	Interdependent networks of commercial entities involved in agriculture and fisheries, food processing and production, storage and distribution, wholesaling and retailing, and preparation and marketing of raw, processed, and ready to eat foods.[21]
**Company engagement with health issues**	Degree to which food and beverage companies orientate their business around public health goals.
**Company profitability**	A company's ability to make profit.
**Consumption of regulated HFSS products**	Individual's intake of foods and beverages within the scope of the regulations[a].
**Consumption of unregulated products**	Individual's intake of foods and beverages that are not within the scope of the regulations[a].
**Definitions**	Information used to define or enforce the regulation[a], including the UK Nutrient Profiling Model and the food categories form the Sugar Reduction Strategy. Importantly, the regulations[a] cover a group of foods that is different from those covered by other UK dietary public health regulations. Enforcement is based on information provided by companies.
**Demand for regulated HFSS products**	Public desire to purchase or consume foods and beverages within the scope of the regulations[a].
**Demand for unregulated products**	Public desire to purchase or consume foods and beverages outside of the scope of the regulations[a].
**Digital surveillance**	Digital data collated by website to inform regulation[a] enforcement.
**Employment**	Number of people employed in the commercial food system.
**Exposure to advertising for unregulated products**	Exposure[b] to adverts for products outside of the scope of the regulations. For foods and beverages, this could be HFSS products within companies' portfolios that are outside of the scope of the regulations, healthier products (e.g., fruit and vegetables), or food delivery companies. Also includes non-food and beverage products and services, but not clear what health impacts they might have.
**Exposure to advertising for regulated HFSS products**	Exposure[b] to advertising for food and beverages within the scope of the regulations[a].

For peer review only - http://bmjopen.bmj.com/site/about/guidelines.xhtml

Statement	Description
**Exposure to unregulated marketing of HFSS products**	Exposure[b] to advertising for all HFSS products on media that are outside of the scope of the regulations[a]. Includes offline advertising (e.g., print media), forms of marketing online that are exempt from the regulations (e.g., in owned media), sponsorship, brand advertising and creative modes of marketing that are hard to capture with regulation.
**Health**	Overall health, including and beyond bodyweight and non-communicable diseases (NCDs).
**Lobbying against further interventions**	Activities undertaken by, or on behalf of, food and beverage companies to resist further policy or regulations.
**Market share**	The size of the total market held by a company. Few companies that each hold a large market share creates a concentrated market.
**Portion size**	Size of food and beverage products in grams or calories, or recommended portion size.
**Price**	Price of food and beverage products, including price discounts.
**Product innovation for unregulated products**	Developing new products that are outside of the scope of the regulations[a], or reformulating existing products so they are no longer within the scope of the regulations. Could include reformulation using artificial ingredients or developing e.g., saltier products that are currently an exempt category. Some categories of products are easier to change than others, and some companies are better able to respond in this way than others.
**Public awareness**	Degree of public awareness of both the regulations[a] and the problems they are trying to address.
**Public support**	Degree of public support for the regulations[a].
**Purchases of regulated HFSS products**	Sales (from company perspective) or purchases (from individual perspective) of food and beverage products within the scope of the regulations[a].
**Purchases of unregulated products**	Sales (from company perspective) or purchases (from individual perspective) of food and beverage products outside of the scope of the regulations[a].
**Regulatory and political landscape**	Wider landscape of regulation and policy, including others relating to marketing (e.g., location and volume price regulations) and COVID-19. The degree to which the regulations[a] harmonise with the wider political and regulatory landscape.
**Regulatory precedent**	Implementation of the regulations[a] serves as precedent for any future regulation.
**Risk of diet-related NCDs**	Risk of developing NCDs influenced by dietary behaviours.
**Social norms around food**	Implicit or explicit beliefs, attitudes, or behaviours about eating, at both an individual and family level.
**Society**	The wider social system in which the food system is embedded.
**Societal shifts**	Exposure[b] to advertising effects social norms and may contribute to societal changes in consumerism and culture.

244 Notes: BMI = body mass index; HFSS = high fat, salt, and sugar; NCD = Non-communicable disease.

For peer review only - http://bmjopen.bmj.com/site/about/guidelines.xhtml

[a] The regulations apply to online and TV advertising for a subset of HFSS products, defined by the 2004 to 2005 UK Nutrient Profiling Model and within particular categories from the Sugar Reduction Strategy. This means there are HFSS products (unregulated HFSS) and non-HFSS products outside of the scope of the regulations.

[b] Exposure is a function of advertising prevalence, but is also dependent on individual-level factors (e.g., frequency of media use).

DISCUSSION

Overview of findings

Using an adapted group concept mapping method in four expert workshops, we developed a concept map to visualise how the proposed TV and online food advertising regulations may impact on the commercial food system, health and society. The concept map illustrates that the pathways between the regulations and these impact domains will be determined by the reactions of stakeholders.

Strengths and limitations

To our knowledge, this is the first cross-sectoral attempt to explicitly theorise how regulations of this kind may impact on the commercial food system, health and society. Incorporating the views of a range of experts with different perspectives and interests allowed us to create a comprehensive articulation of the ways the regulations may positively or negatively affect public health. As with any qualitative research, our map does not claim to be representative of all views, nor comprehensive, of the wider groups that participants represent.[40] Instead, we intended to sample a diverse range of expert views related to food marketing and its regulation. Including participants from diverse sectors is a strength of the study as it enabled the proposed regulations to be theorised expansively. Nonetheless, it is possible that other concepts and pathways may exist but were not captured by our map.

We necessarily invited more individuals than those who ultimately participated. The timing of the data collection period was a common reason for non-participation in the workshops, as it coincided with summer and school holidays in the UK, which may have made it difficult for those with child caring responsibilities to attend. To accommodate individuals' other commitments, we held smaller workshops across various times and days. Doing so increased the participation in our study, but it may have lost some discussion and synergy that larger groups allow.

For peer review only - http://bmjopen.bmj.com/site/about/guidelines.xhtml

We found it difficult to recruit individuals from industry and government organisations. Employees from these sectors rarely have their contact details listed on public-facing websites, unlike those from academia and civil society. Government organisations expressed reluctance to contribute information beyond what was already in the public domain.[41] There may have also been reluctance from industry to engage with our research due to inherent differences between the goals of public health researchers and of the food industry. Including a relatively small number of industry representatives may have limited our final map, and those industry perspectives in our study may be more sympathetic to public health goals than those of the wider sector. However, one of the representatives of industry we did include worked for an umbrella group and so may have a particularly broad perspective to bring. Some of our participants representing other sectors also had previous experience of working with industry. Participants may have also taken part in our study to pursue their own agenda, as industry actors have previously sought to undermine food advertising regulations.[42,43] There are some differences in the contributions made by industry participants compared with non-industry ones (**Appendix 2** and **3**). However, the nature of the workshop content, holding workshops with experts from non-industry sectors, and verifying findings with all participants, left little room for industry interests to overly-dominate our concept map.

Conducting the workshops in person may have achieved different results, as some participants may have felt more able to share sensitive information in person. However, online workshops widened attendance to those who would have been unable to attend in-person. To avoid some of the potential challenges of collecting data using Zoom, we employed several recommended strategies.[44] This included using screen-sharing and clear greetings to develop rapport, using back-up recording devices, holding facilitator briefings to avoid technical issues, and establishing 'house rules' to ease participants' experiences.[44] To maintain participant engagement, workshop duration was limited to two hours, and primarily focused on capturing concepts rather than exhaustively detailing the pathways between them. Though it may have increased participant fatigue and burden, holding longer workshops may have allowed time to capture additional concepts and pathways. As a form of member-checking,[45] we verified the master map with all workshop participants by email, in a further attempt to ensure the final concept map accurately represented participants' contributions and to allow additional comments.

Interpretation of findings

The concept map can be used to illustrate pathways through which the reactions of food and drink companies may serve or undermine the public health goals of the regulations. Here, we describe three potential scenarios: (i) adaptations are made to the regulations in ways that reinforce positive impacts

For peer review only - http://bmjopen.bmj.com/site/about/guidelines.xhtml

on public health (see **Figure 4**); (ii) adaptations are made to the regulations in ways that undermine impacts on public health (see **Figure 5**); and (iii) technicalities of the regulations cover too few unhealthy food products and advertising opportunities to make a substantial difference to public health (see **Figure 6**). As it is unlikely all companies will respond uniformly, a combination of the three scenarios may follow the implementation of the regulations.

Scenario 1: adaptations reinforce positive impacts of the regulations on public health

INSERT FIGURE 4 HERE

Companies may reduce their TV and online advertising for regulated HFSS products, as they will have less opportunity for advertisements. Doing so reduces people's exposure to HFSS adverts, which may prompt corresponding reductions in demand, purchases and consumption of the associated HFSS products. Consequently, this will reduce the total number of calories consumed by individuals, improving health outcomes both associated with, and independent of, body weight.

To make up lost revenue from fewer HFSS product purchases, companies may increase TV and online advertising for their products that are out of the scope of the regulations (e.g., 'spotlighting' low-fat, -salt or -sugar alternatives). They may also engage with diet-related health issues, which could include developing and advertising new products that are out of scope of the regulations, particularly if there is public support for the regulations and corresponding falls in demand for HFSS products. Doing so reduces the proportion of HFSS products (relative to non-HFSS) available in the food system.

Reduced exposure to HFSS adverts may change social norms about the acceptability of consuming HFSS products. It may also change a consumerism mindset that may be encouraged by adverts to over-purchase and consume products. These changes could contribute to societal shifts that reinforce lower demand for HFSS products and change macro-level eating behaviours.

Scenario 2: adaptations undermine impacts of the regulations on public health

INSERT FIGURE 5 HERE

Food and drink companies could also minimise losses incurred by the regulations by redirecting their efforts towards unregulated forms of marketing ('balloon effect'). Companies could increase their expenditure on brand advertising, sports sponsorship, or advertising outdoors or in print or audio media, none of which are intended to be covered by the regulations. In their marketing messaging, companies could also reframe diet-related health issues to position inactive lifestyles as a more substantial contribution to NCDs. It is unclear how this may affect people's total exposure to marketing, and their resultant demand for HFSS products. Companies may also fear the

For peer review only

implementation of further regulations that could affect their performance, and so may lobby against them. Lobbying could change future regulations such that their impact is limited, and in turn, may mean that other, comparable regulations also have less chance of being implemented.

To implement regulations, companies may increase the amount of data they collect about the population. Such data gathering constitutes greater digital surveillance that impacts society (for example, privacy rights),[46] but could also inform more targeted marketing that is known to be highly effective at encouraging sales and consumption.[47–49]

Scenario 3: technicalities hinder potential impacts of the regulations on public health

INSERT FIGURE 6 HERE

The regulations have a specific set of HFSS withing scope, which has notable exemptions such as some salty foods. TV and online advertising for products exempt from the regulations may continue, as may the corresponding purchasing and consumption of these products. Some participants reported that the proposed scope of the regulations differ to that of other policies. Lack of consistency with other regulations may make it costly – perhaps to the point of being futile – for companies to respond to the regulations by developing new products that are compliant with all related regulations. Limited development of new products would restrict the degree of transformation in the food system. Furthermore, unlike other regulations, these advertising regulations are not defined by portion size nor are smaller portion sizes an explicit objective of the regulations. This means there is no incentive for companies to produce smaller product sizes, which could otherwise contribute towards reducing calorie consumption via HFSS products.

As advertising by small and medium enterprises are also exempt from the regulations, larger companies may 'atomise' by creating smaller off-shoot companies, which can continue to advertise and sell HFSS products without limitation by the regulations. Advertising of HFSS outside of the watershed hours will still be permitted on TV and on-demand services, and large HFSS companies can afford the high price of advertising slots likely to occur after 9pm. TV advertising after 9pm may therefore become saturated with HFSS products, which may limit the impact of the regulations on adults' and older teenagers' consumption habits and, by extension, that of the children they are responsible for.

Comparison to existing literature

Many existing models exist to illustrate how food marketing affects behaviour and health (e.g.,[8]) and logic models are regularly produced to illustrate how other diet-related public health regulations may work. Methods for developing such models have evolved to appreciate the complexity of the

For peer review only - http://bmjopen.bmj.com/site/about/guidelines.xhtml

For peer review only

surrounding system in which they reside,[50] but to our knowledge, these have been rarely applied in the context of diet-related health interventions,[51] and not applied to food advertising regulations before. The concept map we developed here is the first we are aware of to show how food marketing regulations may work by interacting with their surrounding system.

The concept map we developed illustrates ways that reactions to the regulations will reinforce or undermine their impact on public health, reinforcing the hypotheses of earlier work. [9] The potential for some of these pathways to exist has been evidenced elsewhere. Analyses have found that 57 of 65 brands associated with HFSS had an easily identifiable HFSS product, and the majority (84%) of these products had an alternative non-HFSS product from the same brand, master brand, parent company, or license holder company brand portfolio in the UK.[52] Evidence also indicates that HFSS companies have reformulated and developed new products in responses to diet-related polices in the UK, such as the Soft Drinks Industry Levy.[53,54] This evidence corresponds with pathways in the map that show how companies could redistribute advertising from regulated to unregulated products.

Pathways that illustrate the risk of food companies undermining the regulations may be particularly plausible given existing evidence has documented industry opposition to HFSS advertising regulations in the UK.[42,43] The UK government's Department for Digital, Culture, Media & Sport impact assessment of the regulations also assumed that a degree of HFSS advertising will be displaced to other media,[31] as has existing research on the TV regulation specifically.[9,55] It is also widely documented in broader literature that efforts to undermine such regulations often form part of wider market strategies that, when exerted by powerful and global corporations, are difficult to address with singular regulations.[56] Our concept map builds on this evidence by elucidating pathways through which regulation may be undermined, from which it may be possible to adapt the proposed regulations or implement additional, complementary ones to maximise the likelihood of the regulations achieving their public health goals.

Implications and further research

As the TV and online advertising regulations are not yet implemented, our findings could be used to augment the proposed legislation to encourage stakeholder reactions that maximise the regulations potential benefits. Ensuring that definitions underpinning the legislation, particularly those relating to product categories, harmonise with other legislation affecting commercial food providers may double-down the incentive to reformulate or develop new, non-HFSS products rather than market HFSS products by other means. Expanding the existing definition to a wider range of foods (e.g., salty snacks currently exempt) could have the same effect. Implementing comparable regulations on other forms of marketing, such as a ban on outdoor advertising of HFSS as has been seen in London,[57] would

For peer review only - http://bmjopen.bmj.com/site/about/guidelines.xhtml

also limit opportunity to redistribute advertising spend for HFSS. Expediting the implementation of other regulations affecting the commercial food system, such as the proposed volume and location price promotion regulations,[58] has similar potential to maximise the benefit of the TV and online advertising ones by limiting opportunities for redistributing efforts to unregulated marketing. Some of these proposed alterations echo responses to the Department of Health and Social Care, and Department For Digital, Culture, Media and Sport 2020 policy consultation.[13] That they were repeated and validated by experts in multiple related fields included in our study reinforce their potential benefit.

The concept map could be used to design a complexity-informed evaluation of the regulations. Complex explanations of intervention impacts appreciate that instead of a singular cause-effect pathway, interventions can act as stimuli that send reverberations across the system in which they reside.[59,60] Complex adaptive system methods also appreciate the role of relationships between actors contributing to a variety of processes operating at different levels and scale to produce intervention outcomes.[50] In doing so, they help avoid finding a wrong answer to important questions,[61,62] and may help measure the impact of unintended consequences alongside the outcomes that the policy sets out to achieve.[63] By explicitly exploring the connections in a complex system, these methods may also identify novel leverage points which could be targeted by future interventions. Though the map developed in our study was not explicitly conceived in systems thinking, it has many systemic qualities (e.g., emphasises the role of relationships) and correlates with other methods such as 'system mapping' that have been identified as a key component of systems-informed evaluations.[50] The concept map could be used to define focal areas for evaluative studies of both the intended and unintended consequences of the regulations or could form the basis of other systems evaluation methods. This could also help establish the relative 'strength' of each relationship.

A benefit of theory, here in the form of a concept map, is that it enables the application of findings elsewhere.[28,29] The presence of food marketing regulations in other countries[64] – albeit different to the ones proposed in the UK - suggests there may be political appetite to learn from the UK's experience. For example, policymakers could refer to the map to consider mechanisms and pathways that are particularly relevant to their country context, and thus important to consider in developing their legislation. Findings that emerge from an evaluation based on the map would also be particularly applicable in other countries and contexts, as the maps clarifies how they are embedded with other stakeholders' adaptations following the implementation of the regulations.

For peer review only - http://bmjopen.bmj.com/site/about/guidelines.xhtml

CONCLUSIONS

While the proposed UK TV and online food advertising regulations will be some of the most restrictive in the world, the concept map developed in this paper illustrates that the extent to which they improve diet-related health will ultimately be determined by stakeholder reactions in the surrounding system. The map may be used as a basis for establishing a comprehensive evaluation of the UK regulations, and to inform similar regulations elsewhere. To realise the full potential of the regulations, UK policymakers may also use the map to identify and prevent loopholes in the legislations before they are implemented.

Contributors

EB, PS, RS, MW AND JA CONCEIVED THE STUDY AND ACQUIRED FUNDING. HF AND JA DEVELOPED THE METHODOLOGY AND ACCOMPANYING RESOURCES AND CONDUCTED THE WORKSHOPS. HF COLLATED AND VALIDATED THE DATA. HF PREPARED THE MANUSCRIPT, AND THE DRAFT VERSIONS WERE CRITICALLY REVIEWED BY EB, PS, RS, MA AND JA. ALL AUTHORS APPROVED THE FINAL MANUSCRIPT.

Funding

This project was funded by the National Institute for Health Research Public Health Research Programme (project number 133570). The views and opinions expressed therein are those of the authors and do not necessarily reflect those of the Public Health Research Programme, NIHR, NHS or the Department of Health. HF, MW, JA are supported by the MRC Epidemiology Unit, University of Cambridge [grant number MC/UU/00006/7].

Competing interests

JA and MW report research grants from the Medical Research Council, the Biotechnology and Biological Sciences Research Council, the Canadian Institutes of Health Research, the Department of Health & Social Care Policy Research Units, and National Institute for Health Research during the conduct of the study. MW is a member of the Expert Advisory Group of the Food Foundation, a Community Interest Company in the UK that is leading work on food insecurity, including coordinating an ongoing independent inquiry into childhood food insecurity, led by the All-Party Parliamentary Group on Hunger and Food Poverty. HF previously worked for a market research company, which conducts research on behalf of many companies, including those from the food and drink industry. HF, JA and MW have submitted evidence to the Department of Health and Social

For peer review only - http://bmjopen.bmj.com/site/about/guidelines.xhtml

Care, and Department For Digital, Culture, Media and Sport 2020 consultation for the regulations under study, available here: https://www.cedar.iph.cam.ac.uk/resources/evidence-submissions/#HFSSadban. RS reports grants from the Medical Research Council, National Institute for Health Research, Wellcome Trust and Food Standards Agency during the conduct of the study. EB reports grants from Public Health England during the conduct of the study. PS reports grants from British Heart Foundation, National Institute for Health Research, Medical Research Council and Wellcome Trust during the conduct of the study. The authors declare no other conflicts of interest.

Data availability statement

Each workshop map is available in **Appendix 2** but recordings are not available as it is not possible to sufficiently anonymise participants.

Exclusive License

I, the Submitting Author has the right to grant and does grant on behalf of all authors of the Work (as defined in the below author licence), an exclusive licence and/or a non-exclusive licence for contributions from authors who are: i) UK Crown employees; ii) where BMJ has agreed a CC-BY licence shall apply, and/or iii) in accordance with the terms applicable for US Federal Government officers or employees acting as part of their official duties; on a worldwide, perpetual, irrevocable, royalty-free basis to BMJ Publishing Group Ltd (“BMJ”) its licensees and where the relevant Journal is co-owned by BMJ to the co-owners of the Journal, to publish the Work in BMJ Open and any other BMJ products and to exploit all rights, as set out in our licence.

The Submitting Author accepts and understands that any supply made under these terms is made by BMJ to the Submitting Author unless you are acting as an employee on behalf of your employer or a postgraduate student of an affiliated institution which is paying any applicable article publishing charge (“APC”) for Open Access articles. Where the Submitting Author wishes to make the Work available on an Open Access basis (and intends to pay the relevant APC), the terms of reuse of such Open Access shall be governed by a Creative Commons licence – details of these licences and which Creative Commons licence will apply to this Work are set out in our licence referred to above.

For peer review only

For peer review only - http://bmjopen.bmj.com/site/about/guidelines.xhtml

References

1 World Health Organization. Set of recommendations on the marketing of foods and non-alcoholic beverages to children. World Health Organization. 2010;:14.https://www.who.int/dietphysicalactivity/publications/recsmarketing/en/ (accessed 3 Nov 2017).

2 Boyland EJ, Nolan S, Kelly B, *et al.* Advertising as a cue to consume: A systematic review and meta-analysis of the effects of acute exposure to unhealthy food and nonalcoholic beverage advertising on intake in children and adults. *American Journal of Clinical Nutrition* 2016;**103**:519–33. doi:10.3945/ajcn.115.120022

3 Smith R, Kelly B, Yeatman H, *et al.* Food Marketing Influences Children's Attitudes, Preferences and Consumption: A Systematic Critical Review. *Nutrients* 2019;**11**:875. doi:10.3390/nu11040875

4 Cairns G. A critical review of evidence on the sociocultural impacts of food marketing and policy implications. *Appetite* 2019;**136**:193–207. doi:10.1016/j.appet.2019.02.002

5 American Marketing Association. Definitions of Marketing. American Marketing Association. https://www.ama.org/AboutAMA/Pages/Definition-of-Marketing.aspx (accessed 22 Oct 2017).

6 McCarthy JE. *Basic Marketing. A Managerial Approach*. Homewood, IL: : Irwin 1964.

7 The Food Foundation. The Broken Plate 2020: The state of the nations Food System. 2020.https://foodfoundation.org.uk/wp-content/uploads/2020/09/FF-Broken-Plate-2020-DIGITAL-FULL.pdf (accessed 6 Mar 2021).

8 Kelly B, King L, Chapman K, *et al.* A hierarchy of unhealthy food promotion effects: Identifying methodological approaches and knowledge gaps. American Journal of Public Health. 2015;**105**:e86–95. doi:10.2105/AJPH.2014.302476

9 Mytton O, Boyland E, Adams J, *et al.* The potential health impact of restricting less-healthy food and beverage advertising on UK television between 05.30 and 21.00 hours: a modelling study. *PLoS Medicine* 2020;**17**:e1003212. doi:10.1371/journal.pmed.1003212

10 Department of Health & Social Care. Tackling obesity: empowering adults and children to live heatlhier lives. 2020.https://www.gov.uk/government/publications/tackling-obesity%02government-strategy/tackling-obesity-empowering-adults-and-children-to-live-

For peer review only

For peer review only - http://bmjopen.bmj.com/site/about/guidelines.xhtml

healthier%02lives#empowering-everyone-with-the-right-information-to-make-healthier-choices (accessed 14 Sep 2020).

11 UK Government. Health and Care Bill. London, UK: : UK Parliament 2021. https://bills.parliament.uk/bills/3022

12 Department of Health and Social Care: Global Public Health Directorate. Childhood obesity: a plan for action, chapter 2. HM Government. 2018.https://assets.publishing.service.gov.uk/government/uploads/system/uploads/attachment_data/file/718903/childhood-obesity-a-plan-for-action-chapter-2.pdf (accessed 24 Mar 2021).

13 HM Government. Introducing further advertising restrictions on TV and online for products high in fat, sugar and salt (HFSS). 2019.https://assets.publishing.service.gov.uk/government/uploads/system/uploads/attachment_data/file/795412/hfss-advertising-consultation-10-april-2019.pdf (accessed 20 Jan 2021).

14 Taillie LS, Busey E, Stoltze FM, *et al.* Governmental policies to reduce unhealthy food marketing to children. *Nutrition Reviews* 2019;**0**:1–32. doi:10.1093/nutrit/nuz059

15 Ofcom. Media Nations 2020: UK report 2020. 2020. https://www.ofcom.org.uk/__data/assets/pdf_file/0010/200503/media-nations-2020-uk-report.pdf

16 Ofcom. Media nations: UK 2021. 2021.

17 Murphy G, Corcoran C, Tatlow-Golden M, *et al.* See, Like, Share, Remember: Adolescents' Responses to Unhealthy- , Healthy- and Non-Food Advertising in Social Media. *International Journal of Environmental Research and Public Health* 2020;**17**. doi:10.3390/ijerph17072181

18 Coates AE, Hardman CA, Halford JCG, *et al.* Social Media Influencer Marketing and Children's Food Intake: A Randomized Trial. *Pediatrics* 2019;**143**:e20182554. doi:10.1542/peds.2018-2554

19 Ertz M, le Bouhart G. The Other Pandemic: A Conceptual Framework and Future Research Directions of Junk Food Marketing to Children and Childhood Obesity. *Journal of Macromarketing* 2022;**42**:30–50. doi:10.1177/02761467211054354

20 Ogilvie D, Adams J, Bauman A, *et al.* Using natural experimental studies to guide public health action: turning the evidence-based medicine paradigm on its head. *Journal of Epidemiology and Community Health* 2020;**74**:203–8. doi:10.1136/jech-2019-213085

For peer review only - http://bmjopen.bmj.com/site/about/guidelines.xhtml

21 White M, Aguirre E, Finegood DT, *et al.* What role should the commercial food system play in promoting health through better diet? *BMJ* 2020;**368**:m545. doi:10.1136/bmj.m545

22 Mora F. Victims of the Balloon Effect: Drug Trafficking and U.S. Policy in Brazil and the Southern Cone of Latin America. *The Journal of Social, Political, and Economic Studies* 1996;**21**:115.

23 Boyland EJ, Harris JL. Regulation of food marketing to children: Are statutory or industry self-governed systems effective? *Public Health Nutrition* 2017;**20**:761–4. doi:10.1017/S1368980017000465

24 Coleman PC, Hanson P, Rens T van, *et al.* A rapid review of the evidence for children’s TV and online advertisement restrictions to fight obesity. *Preventive Medicine Reports* 2022;:101717. doi:10.1016/j.pmedr.2022.101717

25 Jiménez-Marín G, Zambrano RE, Galiano-Coronil A, *et al.* Food and Beverage Advertising Aimed at Spanish Children Issued through Mobile Devices: A Study from a Social Marketing and Happiness Management Perspective. *International Journal of Environmental Research and Public Health* 2020;**17**:1–18. doi:10.3390/ijerph17145056

26 Sing F, Mackay S, Culpin A, *et al.* Food Advertising to Children in New Zealand: A Critical Review of the Performance of a Self-Regulatory Complaints System Using a Public Health Law Framework. *Nutrients* 2020;**12**. doi:10.3390/nu12051278

27 Lee Y, Yoon J, Chung SJ, *et al.* Effect of TV food advertising restriction on food environment for children in South Korea. *Health Promotion International* 2017;**32**:25–34. doi:10.1093/heapro/dat078

28 Ogilvie D, Cummins S, Petticrew M, *et al.* Assessing the Evaluability of Complex Public Health Interventions: Five Questions for Researchers, Funders, and Policymakers. *Milbank Quarterly* 2011;**89**:206–25. doi:10.1111/j.1468-0009.2011.00626.x

29 Rosas SR. Concept mapping as a technique for program theory development: An illustration using family support programs. *American Journal of Evaluation* 2005;**26**:389–401. doi:10.1177/1098214005278760

30 Burke JG, O'Campo P, Peak GL, *et al.* An introduction to concept mapping as a participatory public health research method. *Qualitative Health Research* 2005;**15**:1392–410. doi:10.1177/1049732305278876

For peer review only

For peer review only - http://bmjopen.bmj.com/site/about/guidelines.xhtml

31 Anderson LA, Slonim A. Perspectives on the strategic uses of concept mapping to address public health challenges. *Evaluation and Program Planning* 2017;**60**:194–201. doi:10.1016/j.evalprogplan.2016.08.011

32 Trochim WMK. An introduction to concept mapping for planning and evaluation. *Evaluation and Program Planning* 1989;**12**. doi:10.1016/0149-7189(89)90016-5

33 Tong A, Sainsbury P, Craig J. Consolidated criteria for reporting qualitative research (COREQ): a 32-item checklist for interviews and focus groups. *International Journal for Quality in Health Care* 2007;**19**:349–57. doi:10.1093/intqhc/mzm042

34 Albury C, Pope C, Shaw S, *et al.* Gender in the consolidated criteria for reporting qualitative research (COREQ) checklist. *International Journal for Quality in Health Care* 2021;**33**:2021. doi:10.1093/intqhc/mzab123

35 Braun V, Clarke V. To saturate or not to saturate? Questioning data saturation as a useful concept for thematic analysis and sample-size rationales. *Qualitative Research in Sport, Exercise and Health* 2019;:1–16. doi:10.1080/2159676X.2019.1704846

36 van der Graaf P, Forrest LF, Adams J, *et al.* How do public health professionals view and engage with research? A qualitative interview study and stakeholder workshop engaging public health professionals and researchers. *BMC Public Health* 2017;**17**:1–10. doi:10.1186/s12889-017-4896-1

37 Forde H, Solomon-Moore E. A Qualitative Study to Understand the Potential Efficacy of an Information-Based Sugar Reduction Intervention among Low Socioeconomic Individuals in the UK. *International Journal of Environmental Research and Public Health* 2019;**16**:413. doi:10.3390/ijerph16030413

38 Penney T, Adams J, Briggs A, *et al.* Evaluation of the impacts on health of the proposed UK industry levy on sugar sweetened beverages: developing a systems map and data platform, and collection of baseline and early impact data. 2017.

39 Chatham House. Chatham House Rule. 2021.https://www.chathamhouse.org/about-us/chatham-house-rule (accessed 18 Aug 2021).

40 Savona N, Macauley T, Aguiar A, *et al.* Identifying the views of adolescents in five European countries on the drivers of obesity using group model building. *European Journal Of Public Health* 2021;:ckaa251. doi:10.1093/eurpub/ckaa251

For peer review only

For peer review only - http://bmjopen.bmj.com/site/about/guidelines.xhtml

41 Department for Digital Culture Media and Sport. Impact assessment: introducing a 2100 to 0530 watershed on TV and online restriction for paid advertising of food and drink that are high in fat, salt or sugar (HFSS) products. London, UK: 2021.

42 Carters-White L, Chambers S, Skivington K, *et al.* Whose rights deserve protection? Framing analysis of responses to the 2016 committee of advertising practice consultation on the non-broadcast advertising of foods and soft drinks to children. *Food Policy* 2021;**104**:102139. doi:10.1016/j.foodpol.2021.102139

43 Lauber K, Hunt D, Gilmore A, *et al.* Corporate political activity in the context of unhealthy food advertising restrictions across Transport for London: A qualitative case study. *PLoS Medicine* 2021;**18**:e1003695. doi:10.1371/journal.pmed.1003695

44 Boland J, Banks S, Krabbe R, *et al.* A COVID-19-era rapid review: using Zoom and Skype for qualitative group research. *Public Health Research & Practice* 2021;:1–9. doi:10.17061/phrp31232112

45 Birt L, Scott S, Cavers D, *et al.* Member Checking: A Tool to Enhance Trustworthiness or Merely a Nod to Validation? *Qualitative Health Research* 2016;**26**:1802–11. doi:10.1177/1049732316654870

46 Tatlow-Golden M, Garde A. Digital food marketing to children: Exploitation, surveillace and rights violations. *Global Food Security* 2020;**27**:100423. doi:10.1016/j.gfs.2020.100423

47 Montgomery KC, Chester J. Interactive Food and Beverage Marketing: Targeting Adolescents in the Digital Age. Journal of Adolescent Health. 2009;**45**. doi:10.1016/j.jadohealth.2009.04.006

48 de Jans S, van de Sompel D, Hudders L, *et al.* Advertising targeting young children: an overview of 10 years of research (2006–2016). *International Journal of Advertising* 2019;**38**:173–206. doi:10.1080/02650487.2017.1411056

49 Harris JL, Frazier III W, Kumanyika SK, *et al.* Increasing disparities in unhealthy food advertising targeted to Hispanic and Black youth. 2019.

50 McGill E, Er V, Penney T, *et al.* Evaluation of public health interventions from a complex systems perspective: a research methods review. *Social Science & Medicine* 2021;**272**:113697. doi:10.1016/j.socscimed.2021.113697

For peer review only

For peer review only - http://bmjopen.bmj.com/site/about/guidelines.xhtml

51 Penney TL, Adams J, Briggs A, *et al.* Theorising how the UK soft drinks industry levy could impact population level diet and health: development of a multi-sectoral systems map. In: *International Society for Behavioural Nutrition and Physical Activity*. 2017. 203.

52 Cheek O, Froguel A, Clark M. Analysis on the potential for UK's leading food and soft drink brands to switch to marketing their non-HFSS products. London: 2021.

53 Pell D, Mytton O, Penney TL, *et al.* Changes in soft drinks purchased by British households associated with the UK soft drinks industry levy: controlled interrupted time series analysis. *The BMJ* 2021;**372**:n254. doi:10.1136/bmj.n254

54 Pell D, Penney T, Mytton O, *et al.* Anticipatory changes in British household purchases of soft drinks associated with the announcement of the Soft Drinks Industry Levy: a controlled interrupted time series analysis. *PLoS Medicine* 2020;**17**:e1003269. doi:10.1371/journal.pmed.1003269

55 Griffith R, Connell MO, Smith K, *et al.* The potential impacts of banning television advertising of HFSS food and drink before the watershed: Report produced for the Obesity Policy Research Unit. Institute for Fiscal Studies. 2019.https://www.ucl.ac.uk/obesity-policy-research-unit/sites/obesity-policy-research-unit/files/potentialimpactofban_new.pdf (accessed 11 Jan 2021).

56 Wood B, Williams O, Nagarajan V, *et al.* Market strategies used by processed food manufacturers to increase and consolidate their power: a systematic review and document analysis. *Globalization and health* 2021;**17**:17. doi:10.1186/s12992-021-00667-7

57 Mayor of London. Press release: Mayor confirms ban on junk food advertising on transport network. Mayor of London. 2018.https://www.london.gov.uk/press-releases/mayoral/ban-on-junk-food-advertising-on-transport-network-0 (accessed 24 Mar 2021).

58 Department of Health and Social Care. Restricting promotions of products high in fat, sugar and salt by location and by price: government response to public consultation. 2020.https://www.gov.uk/government/consultations/restricting-promotions-of-food-and-drink-that-is-high-in-fat-sugar-and-salt/outcome/restricting-promotions-of-products-high-in-fat-sugar-and-salt-by-location-and-by-price-government-response-to-public-consultati (accessed 14 Mar 2021).

59 Shiell A, Hawe P, Gold L. Complex interventions or complex systems? Implications for health economic evaluation. *BMJ* 2008;**336**:1281–3. doi:10.1136/bmj.39569.510521.AD

For peer review only

For peer review only - http://bmjopen.bmj.com/site/about/guidelines.xhtml

60 Petticrew M. When are complex interventions “complex”? When are simple interventions “simple”? *European Journal of Public Health* 2011;**21**:397–8. doi:10.1093/eurpub/ckr084

61 Rutter H, Savona N, Glonti K, *et al.* The need for a complex systems model of evidence for public health. *The Lancet* 2017;**390**:2602–4. doi:10.1016/S0140-6736(17)31267-9

62 Fink DS, Keyes KM. Wrong answers: when simple interpretations create complex problems. In: El-Sayed AM, Galea S, eds. *Systems science and population health*. New York: : Oxford University Press 2017. 25–36.

63 Roux AVD. Complex systems thinking and current impasses in health disparities research. *American Journal of Public Health* 2011;**101**:1627–34. doi:10.2105/AJPH.2011.300149

64 World Cancer Research Fund International. NOURISHING Database. https://www.wcrf.org/int/policy/nourishing-database (accessed 11 Jun 2019).

FIGURE TITLES

Figure 1. Summary of method used to develop the concept map

Figure 2. Examples of mapping concepts and pathways using Miro

Figure 3. Concept map of pathways through which the proposed UK TV and online advertising regulations may affect the commercial food system, health and society

Figure 4. Illustration of concept map use: scenario 1

Figure 5. Illustration of concept map use: scenario 2

Figure 6. Illustration of concept map use: scenario 3

For peer review only

For peer review only - http://bmjopen.bmj.com/site/about/guidelines.xhtml

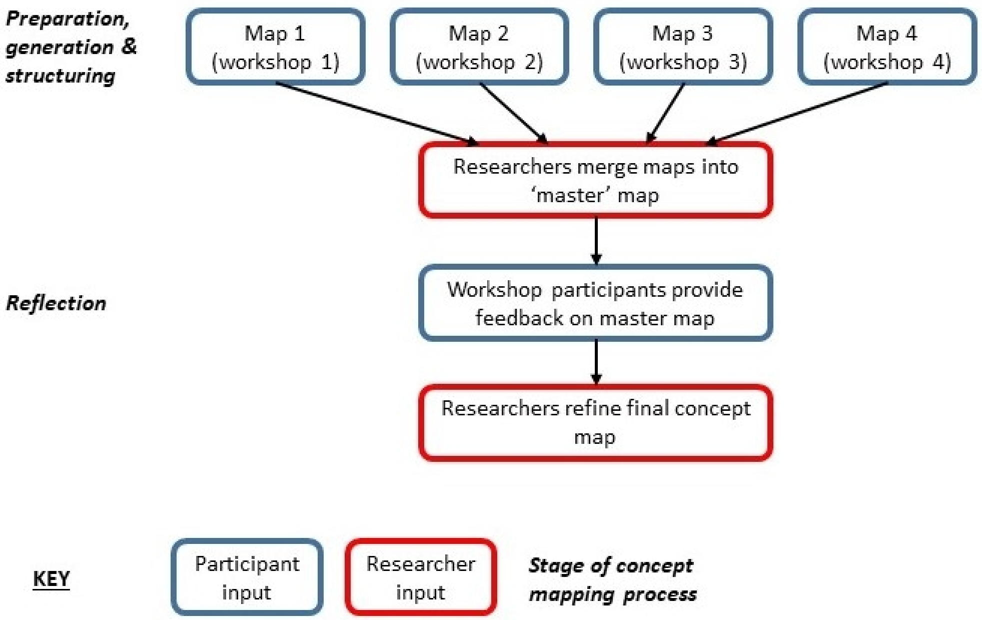

Figure 1 Summary of method used to develop the concept map

176x115mm (96 x 96 DPI)

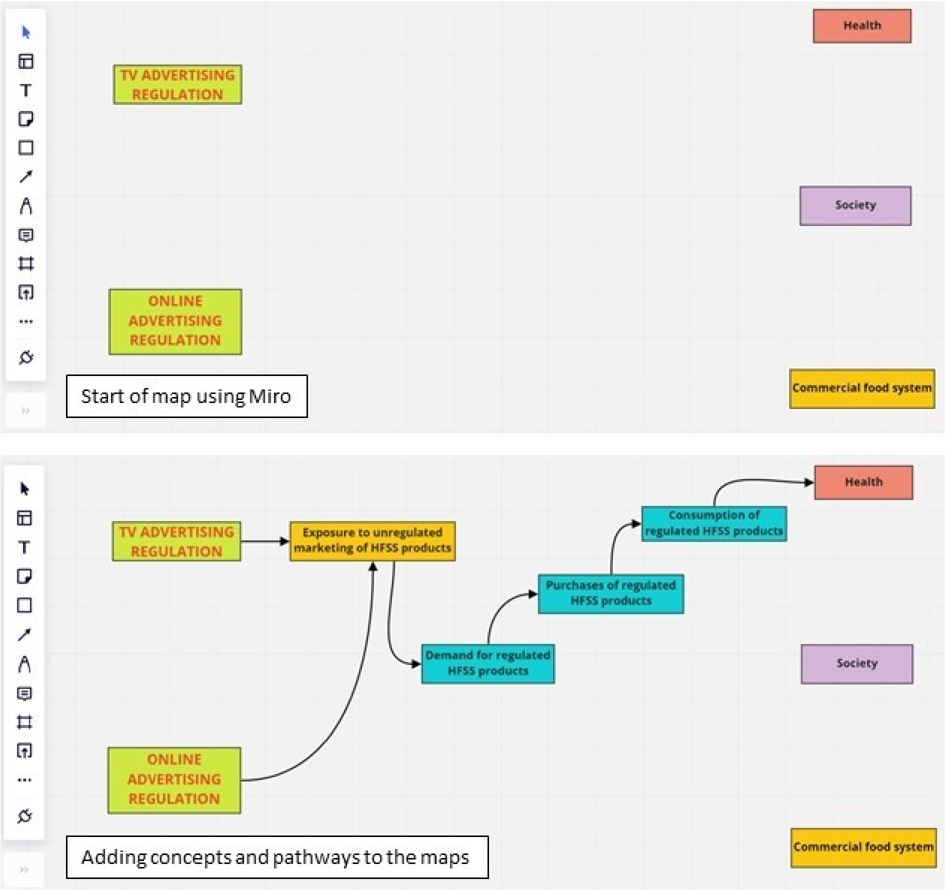

Figure 2 Examples of mapping concepts and pathways using Miro

176x158mm (96 x 96 DPI)

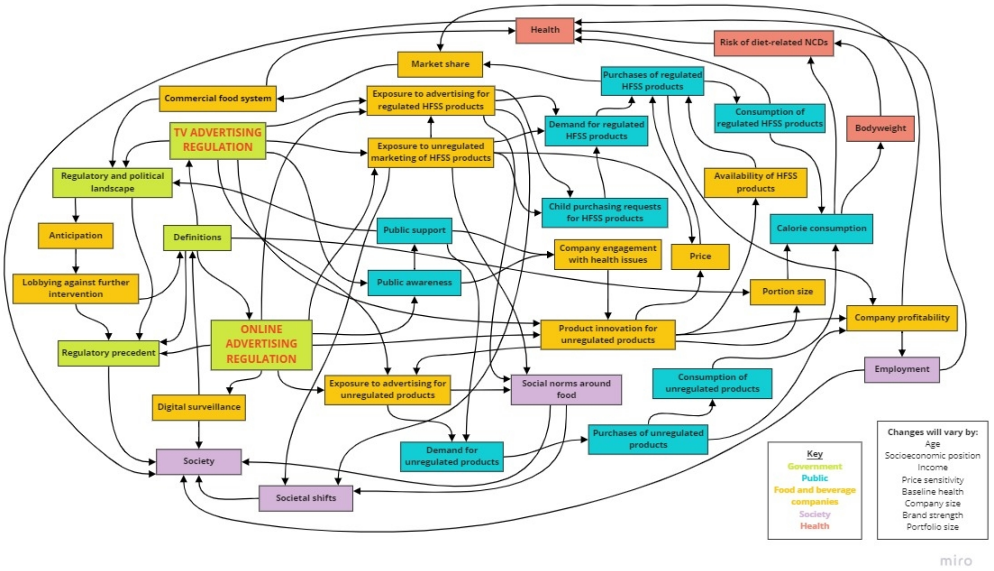

Figure 3 Concept map of pathways through which the proposed UK TV and online advertising regulations may affect the commercial food system, health

391x231mm (72 x 72 DPI)

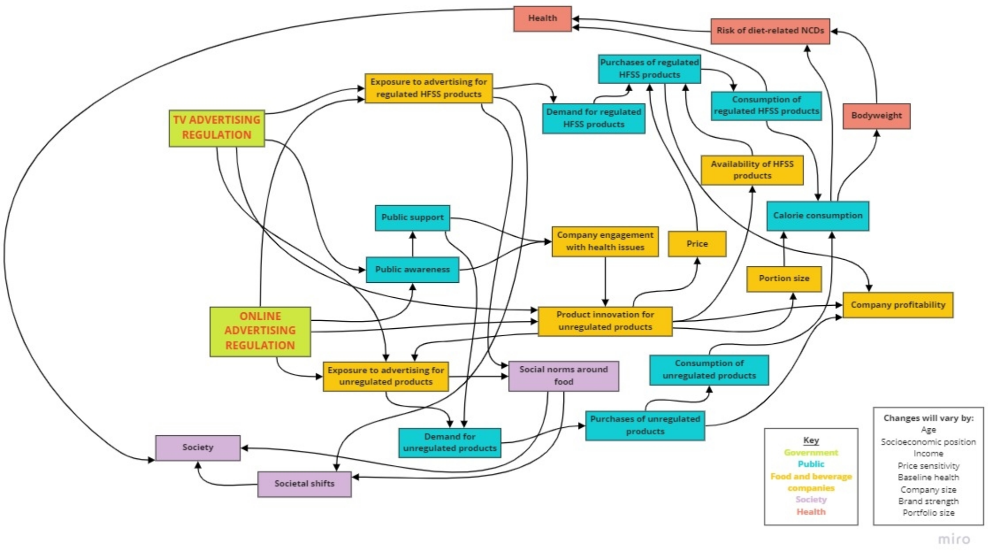

Scenario 1: adaptations reinforce positive impacts of the regulations on public health

398x226mm (72 x 72 DPI)

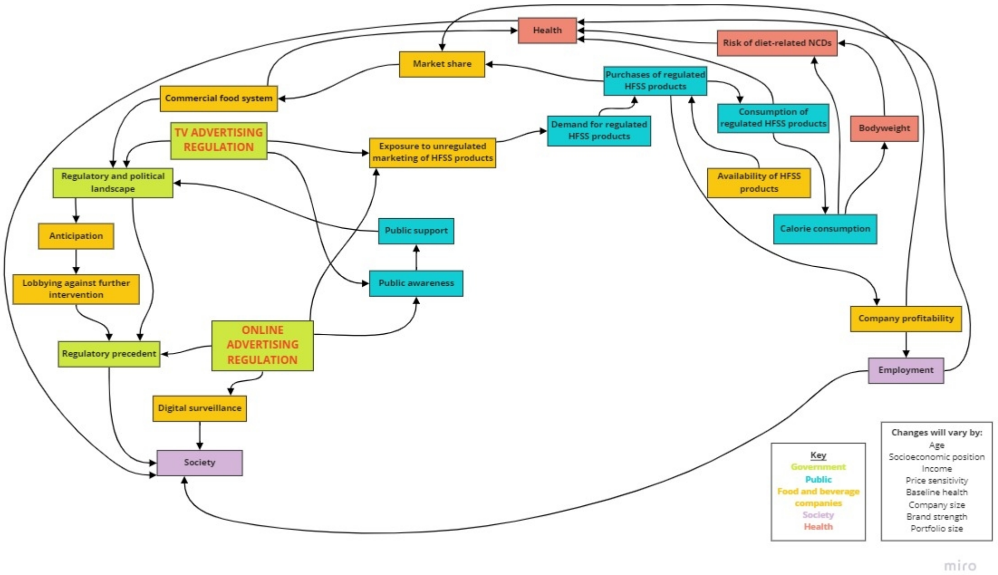

Scenario 2: adaptations undermine impacts of the regulations on public health

389x232mm (72 x 72 DPI)

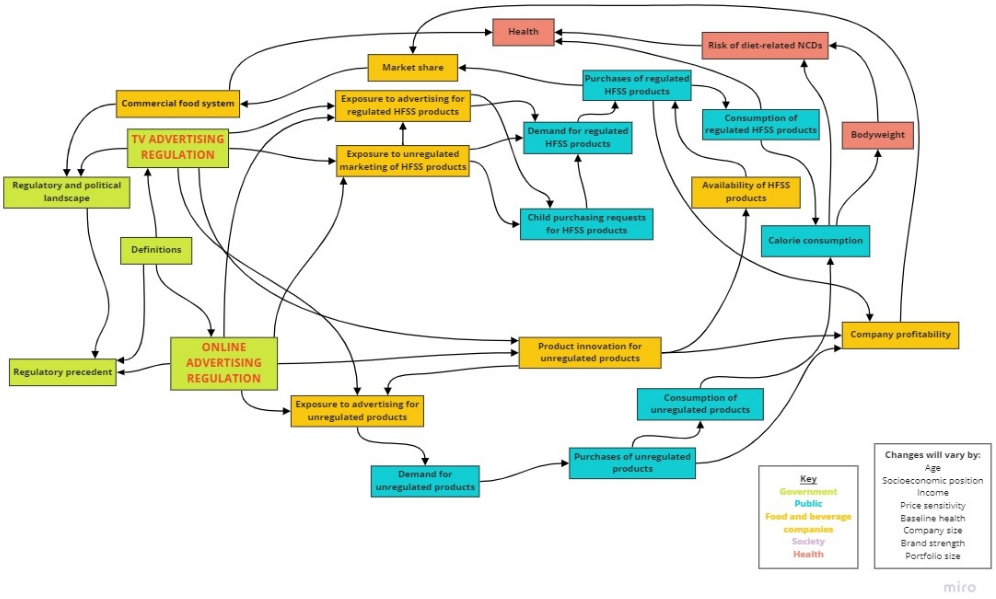

Scenario 3: technicalities hinder potential impacts of the regulations on public health

383x236mm (72 x 72 DPI)

Appendix 1: Consolidated criteria for reporting qualitative studies (COREQ): 32-item checklist

No.	Item	Guide questions/description	Section
**Domain 1: Research team and reflexivity**			
Personal characteristics			
1.	Interviewer/facilitator	Which author/s conducted the interviews or focus groups?	6
2.	Credentials	What were the researcher's credentials? e.g., PhD, MD	Title page
3.	Occupation	What was their occupation at the time of the study?	Title page
4.	Gender	Was the researcher male or female?	See note
5.	Experience and training	What experience or training did the researcher have?	6
Relationship with participants			
6.	Relationship established	Was a relationship established prior to study commencement?	6
7.	Participant knowledge of the interviewer	What did the participants know about the research? e.g., personal goals, reasons for doing the research	6
8.	Interviewer characteristics	What characteristics were reported about the interviewer/facilitator? e.g., bias, assumptions, reasons and interests in the research topic	N/A
**Domain 2: study design**			
Theoretical framework			
9.	Methodological orientation and Theory	What methodological orientation was stated to underpin the study? e.g., grounded theory, discourse analysis, ethnography, phenomenology, content analysis	6
Participant selection			
10.	Sampling	How were the participants selected? e.g., purposive, convenience, consecutive, snowball	6
11.	Method of approach	How were participants approached? e.g., face-to-face, telephone, mail, email	6
12.	Sample size	How many participants were in the study?	8
13.	Non-participation	How many people refused to participate or dropped out? Reasons?	N/A
Setting			
14.	Setting of data collection	Where was the data collected? e.g., home, clinic, workplace	6-7
15.	Presence of non-participants	Was anyone else present besides the participants and researchers?	N/A
16.	Description of sample	What are the important characteristics in the sample? e.g., demographic data, date	8
Data collection			
17.	Interview guide	Were questions, prompts, guides provided by the authors? Was it pilot tested?	6
18.	Repeat interviews	Were repeat interviews carried out? If yes, how many?	N/A
19.	Audio/visual recording	Did the research use audio or visual recording to collect the data?	7
20.	Field notes	Were field notes made during and/or after the interview or focus group?	7
21.	Duration	What was the duration of the interviews or focus group?	6
22.	Data saturation	Was data saturation discussed?	6
23.	Transcripts returned	Were transcripts returned to participants for comment and/or correction?	8

For peer review only - http://bmjopen.bmj.com/site/about/guidelines.xhtml

No.	Item	Guide questions/description	Section
**Domain 3: analysis and findings**			
Data analysis			
24.	Number of data coders	How many data coders coded the data?	N/A
25.	Description of the coding tree	Did authors provide a description of the coding tree?	N/A
26.	Derivation of themes	Were themes identified in advance or derived from the data?	N/A
27.	Software	What software, if applicable, was used to manage the data?	6-7
28.	Participant checking	Did participants provide feedback on the findings?	8
Reporting			
29.	Quotations presented	Were participant quotations presented to illustrate the themes/findings? Was each quotation identified? e.g., participant number	N/A
30.	Data and findings consistent	Was there consistency between the data presented and the findings?	9
31.	Clarity of major themes	Were major themes clearly presented in the findings?	N/A
32.	Clarity of minor themes	Is there a description of diverse cases or discussion of minor themes?	N/A

Taken from: Tong A, Sainsbury P, Craig J. Consolidated criteria for reporting qualitative research (COREQ): a 32-item checklist for interviews and focus groups. *Int J Qual Health Care*. 2007;19(6):349-357. doi:10.1093/intqhc/mzm042

Question on gender omitted in response to recent update: Albury C, Pope C, Shaw S, et al. Gender in the consolidated criteria for reporting qualitative research (COREQ) checklist. *International Journal for Quality in Health Care*. 2021;33(4):2021. doi:10.1093/intqhc/mzab12

For peer review only

Appendix 2*:* Maps 1-4 produced by each workshop

Workshop 1 Map

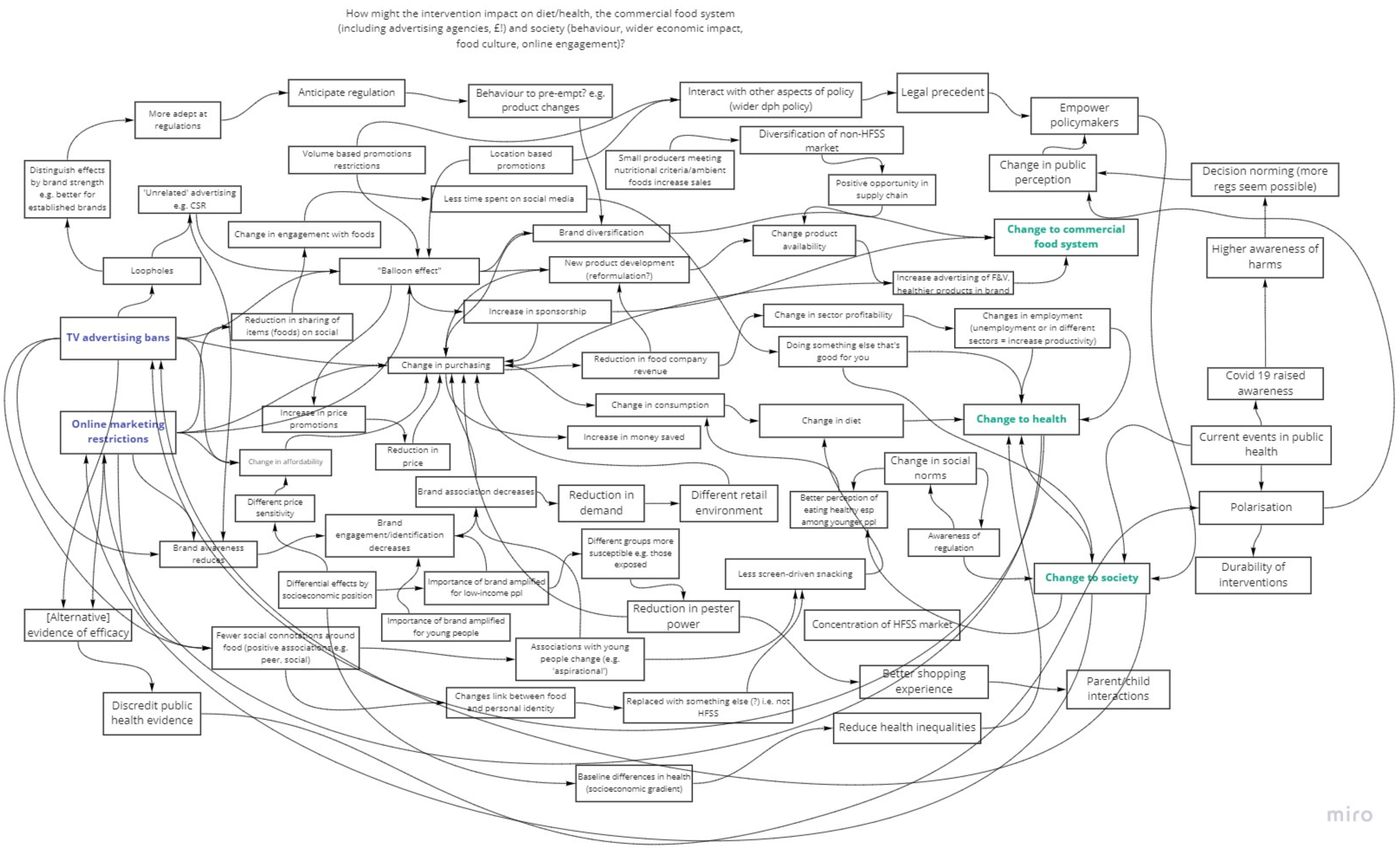

Workshop 2 Map

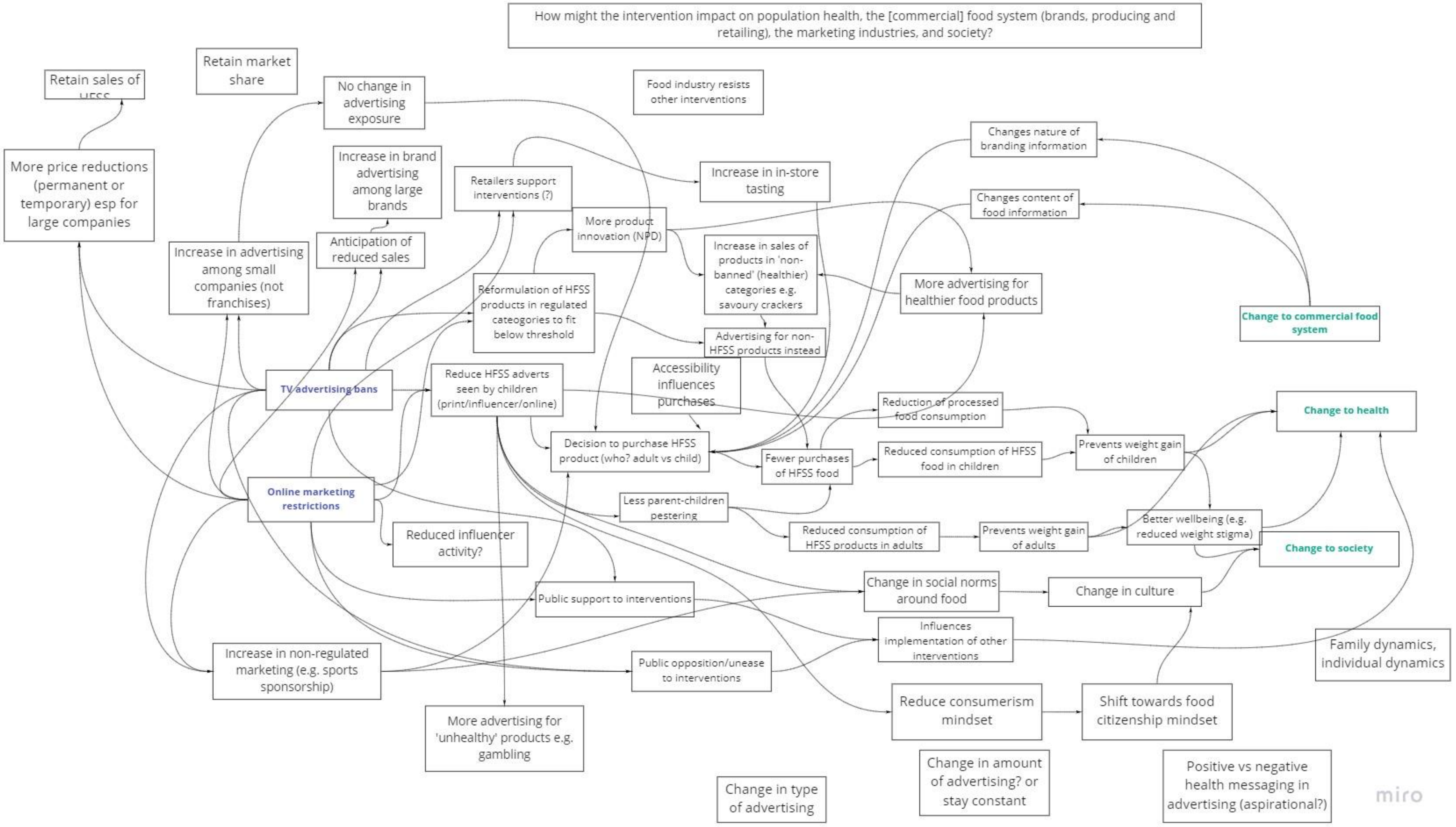

Workshop 3 Map

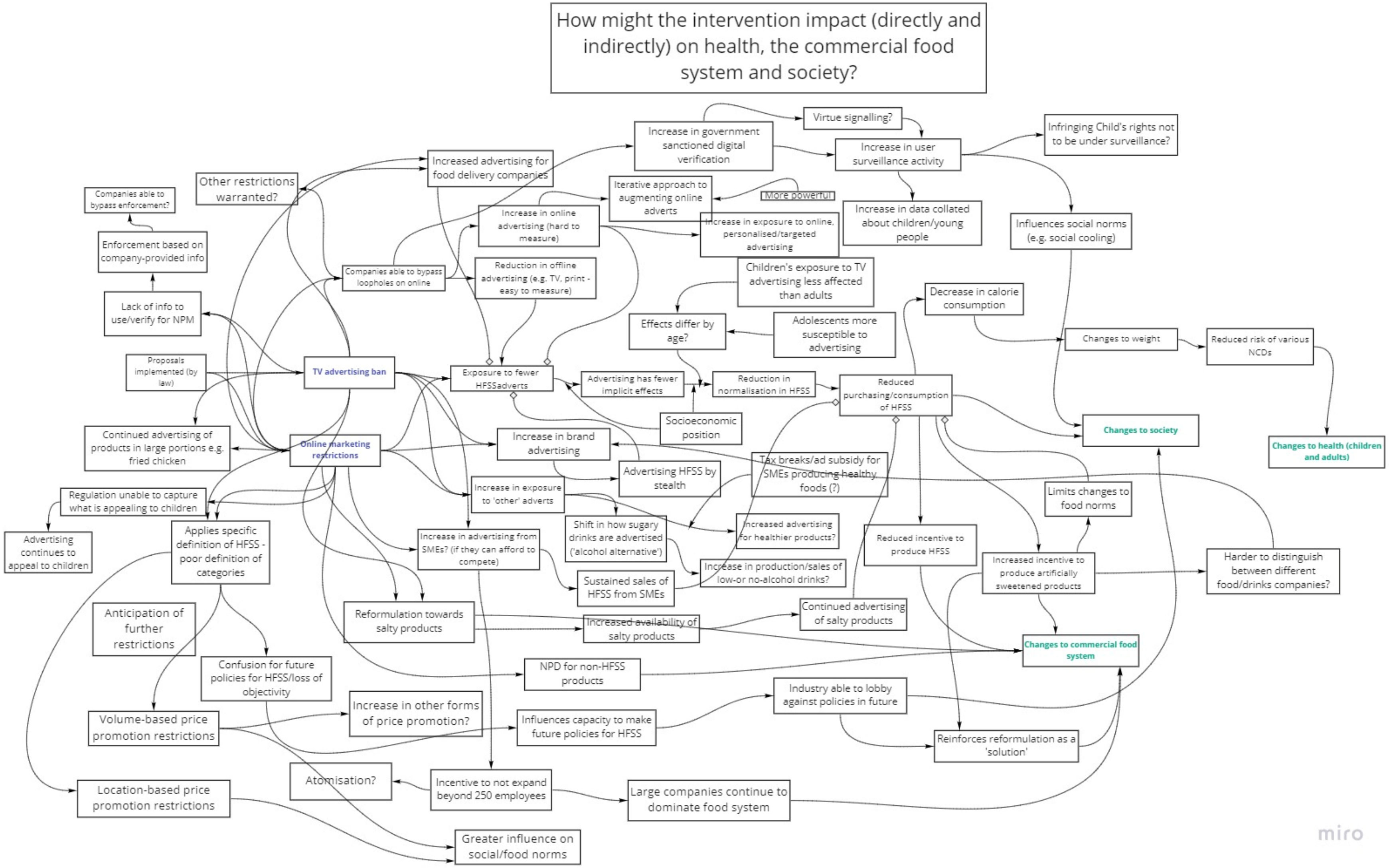

Workshop 4 Map

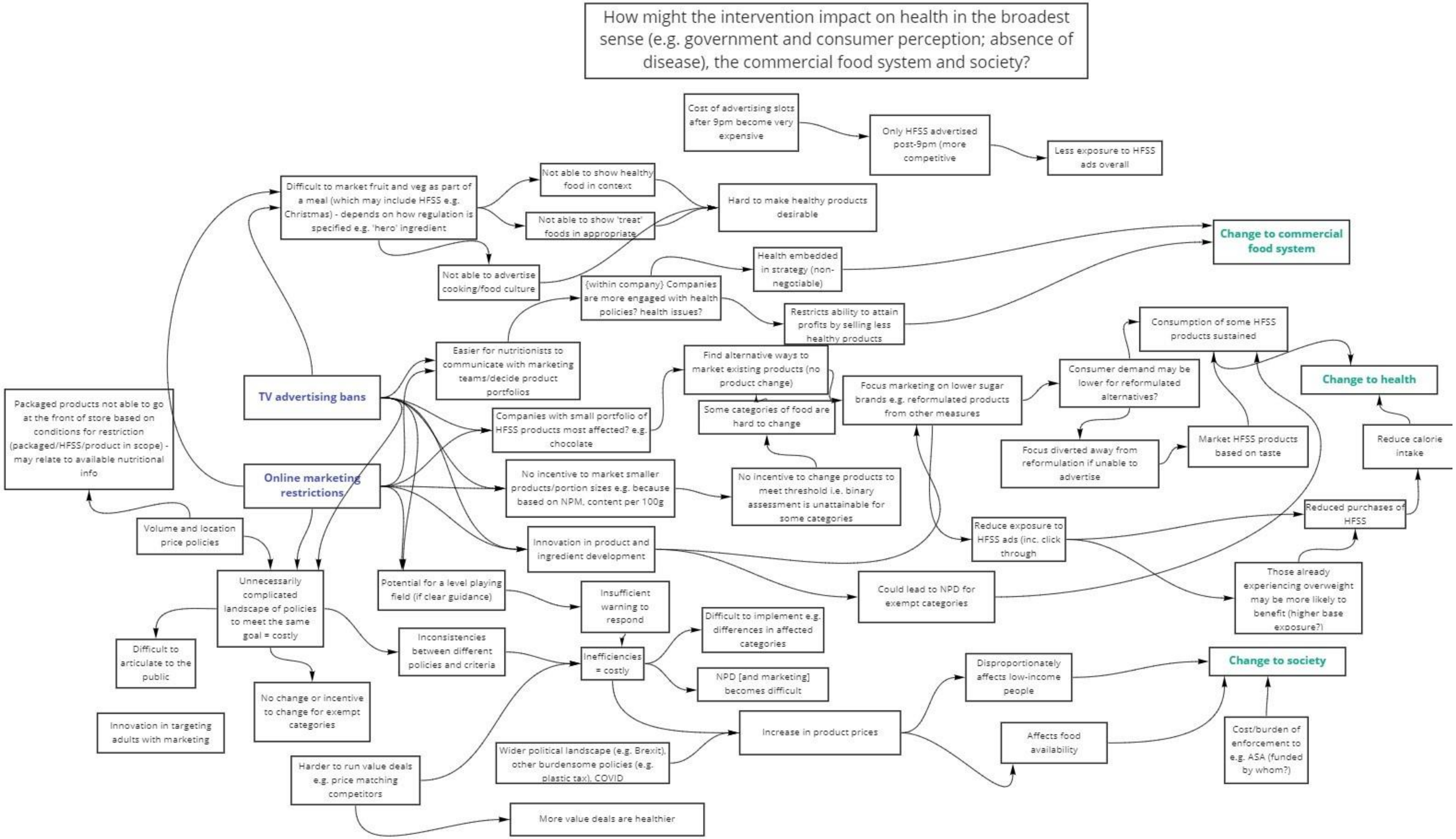

Appendix 3: Concepts from workshop maps and resulting concepts in the final map

Workshop 1 (non-industry)	Workshop 2 (non-industry)	Workshop 3 (non-industry)	Workshop 4 (industry)	Final map
More adept at regulations	Anticipation of reduced sales	Anticipating further restrictions		**Anticipation**
Behaviour to pre-empt? e.g., product changes		Proposals implemented by law		
Anticipate regulation				
Change product availability			Affects food availability	**Availability of HFSS products**
	Prevents weight gain of children	Changes to weight		**Bodyweight**
	Prevents weight gain of adults			
		Decrease in calorie consumption	Reduced calorie intake	**Calorie consumption**
Parent/child interactions	Less parent-children pestering			**Child purchasing requests for HFSS products**
Reduction in pester power				
Better shopping experience				
				**Commercial food system**
			{within company} companies are more engaged with health policies? Health issues?	**Company engagement with health issues**
			Health embedded in strategy (non-negotiable)	
			Easier for nutritionists to communicate with marketing teams	
Reduction in food company revenue			Restricts ability to attain profits by selling less healthy products	**Company profitability**

For peer review only - http://bmjopen.bmj.com/site/about/guidelines.xhtml

Workshop 1 (non-industry)	Workshop 2 (non-industry)	Workshop 3 (non-industry)	Workshop 4 (industry)	Final map
Change in sector profitability			Cost/burden of enforcement to e.g. ASA (funded by whom?)	
			Inefficiencies = costly	
Less screen-driven snacking	Reduced consumption of HFSS products in adults	Reduced purchasing/ consumption of HFSS	Consumption of some HFSS products sustained	**Consumption of regulated HFSS products**
Change in consumption	Reduction of processed food consumption			
Doing something else that's good for you	Reduced consumption of HFSS products in children			
Change in diet				**Consumption of unregulated products**
		Applies specific definition of HFSS - poor definitions of categories	Inconsistencies between different policies and criteria	**Definitions**
		Enforcement based on company-provided info		
		Lack of info to use/verify for NPM		
Reduction in demand				**Demand for regulated HFSS products**
Increase in money saved				
			Consumer demands may be lower for reformulated alternatives?	**Demand for unregulated products**
		Increase in user surveillance activity		**Digital surveillance**
		Infringing Child's rights not to be under surveillance?		
		Increase in surveillance activity		

For peer review only - http://bmjopen.bmj.com/site/about/guidelines.xhtml

Workshop 1 (non-industry)	Workshop 2 (non-industry)	Workshop 3 (non-industry)	Workshop 4 (industry)	Final map
		Increase in data collated about children/young people		
Changes in employment		Incentive to not expand beyond 250 employees		**Employment**
Reductions in sharing of foods on social media	No change in advertising exposure	Exposure to fewer HFSS adverts	Less exposure to ads overall	**Exposure to advertising for regulated HFSS products**
	Reduced influencer activity?	Children's exposure to TV advertising less affected than adults	Reduce exposure to HFSS ads (inc. through click through)	
	Change in amount of advertising or stay constant	Advertising has fewer implicit effects	Market HFSS products based on taste	
	Reduce HFSS adverts seen by children	More powerful	Only HFSS advertised post-9pm (more competitive)	
	Positive vs. negative health messaging in advertising (aspirational)	Virtue signalling		
	Changes content of food information	Harder to distinguish between different food/drinks companies		
		Iterative approach to augmenting online adverts		
Replaced with something else (?) i.e., not HFSS	Advertising for non-HFSS products instead	Exposure to non-HFSS adverts	Focus marketing on lower sugar brands e.g., reformulated products from other measures	**Exposure to advertising for unregulated products**
Increase advertising of F&V, healthier products in brand	More advertising for healthier food products	Increased advertising for healthier products	More value deals are healthier	
	More advertising for 'unhealthy products' e.g., gambling	Increase in exposure to 'other' adverts	Cost of advertising slots after 9pm become very expensive	
		Continued advertising of salty products		
		Increased advertising for food delivery companies		

For peer review only - http://bmjopen.bmj.com/site/about/guidelines.xhtml

Workshop 1 (non-industry)	Workshop 2 (non-industry)	Workshop 3 (non-industry)	Workshop 4 (industry)	Final map
Balloon effect	Change in type of advertising	Increase in exposure to online, personalised/targeted advertising	Difficult to market fruit and veg as part of a meal (which may include HFSS e.g., Christmas) - depends on how regulation is specified e.g. 'hero' ingredient	**Exposure to unregulated marketing of HFSS products**
Increase in sponsorship	Increase in non-regulated marketing (e.g., sports sponsorship)	Advertising continues to appeal to children	Not able to show healthy food in context	
‘Unrelated' advertising e.g., CSR	Increase in in-store tasting	Companies able to bypass enforcement	Not able to show 'treat' foods in appropriate	
Loopholes	More price reductions especially for large companies	Increase in online advertising (hard to measure)	Hard to make healthy products desirable	
Location based promotions	Increase in advertising among small companies (not franchises)	Companies able to bypass loopholes online	Focus diverted away from reformulation if unable to advertise	
Increase in price promotions	Changes nature of branding information	Shift in how sugary drinks are advertised (alcohol alternatives)	Not able to advertise cooking/food culture	
Brand awareness reduces	Increase in brand advertising among large brands	Reduction in offline advertising (e.g., TV, print - easy to measure)	Find alternative ways to market existing products (no product change)	
Brand engagement/identification decreases		Continued advertising of products in large portions e.g., fried chicken	Innovation in targeting adults with marketing	
Brand association decreases		Advertising HFSS by stealth		
		Increase in other forms of price promotion?		
		Regulation unable to capture what is appealing to children		
	Better wellbeing (e.g., reduced weight stigma)			**Health**
Alternative evidence of efficacy	Food industry resists other interventions	Industry able to lobby against policies in future		**Lobbying against further interventions**

Workshop 1 (non-industry)	Workshop 2 (non-industry)	Workshop 3 (non-industry)	Workshop 4 (industry)	Final map
Discredit public health evidence				
Concentration of HFSS market	Retain market share	Large companies continue to dominate food system		**Market share**
		Atomisation?		
		Continued advertising of products in large portions e.g., fried chicken	No incentive to market smaller products/portion sizes e.g., because based on NPM, content per 100g	**Portion size**
Change in affordability	More price reductions especially for large companies	Increase in other forms of price promotion?	Increase in product prices	**Price**
Reduction in price			Harder to run value deals e.g., price matching competitors	
Diversification of non-HFSS markets	More product innovation (NPD)	NPD for non-HFSS products	Could lead to NPD for exempt categories	
Positive opportunity in supply chain	Reformulation of HFSS products in regulated categories to fit below threshold	Reformulation towards salty products	Some categories of food are hard to change	
Brand diversification		Reduced incentive to produce HFSS	NPD [and marketing] becomes difficult	
New product development (reformulation?)		Increased incentive to produce artificially sweetened products	No incentive to change products to meet threshold i.e., binary assessment is unattainable for some categories	**Product innovation for unregulated products**
			No change or incentive to change for exempt categories	
			Innovation in product and ingredient development	
Change in public perception				
Awareness of regulation				**Public awareness**
Higher awareness of harms				

For peer review only - http://bmjopen.bmj.com/site/about/guidelines.xhtml

Workshop 1 (non-industry)	Workshop 2 (non-industry)	Workshop 3 (non-industry)	Workshop 4 (industry)	Final map
Better perception of eating healthy especially among younger people				
	Public support for interventions		Difficult to articulate to the public	**Public support**
	Public opposition/unease to interventions			
Change in purchasing	Decision to purchases HFSS product (who? Adult vs child)	Reduced purchasing/consumption of HFSS	Reduced purchases of HFSS	**Purchases of HFSS regulated products**
	Fewer purchases of HFSS food			
	Retain sales of HFSS			
Chang e in purchasing	Increase in sales of products in 'non-banned' healthier categories e.g., savoury crackers	Increased availability of salty products		**Purchases of unregulated products**
		Increase in production/sales of low or no alcoholic drinks		
Durability of interventions	Retailers support interventions	Volume-based price promotion restrictions	Wider political landscape (e.g., Brexit) other burdensome policies (e.g., plastic tax, covid)	
Interact with other aspects of DPH policy		Location-based price promotion restrictions	Volume and location price policies	
Location based promotions		Other restrictions warranted	Unnecessarily complicated landscape of policies to meet the same goal = costly	
Covid-19 raised awareness		Reinforces reformulation as 'solution'	Potential for a level playing field (if clear guidance)	**Regulatory and political landscape**
Current events in public health		Tax breaks/ad subsidy for SMEs producing healthy foods (?)	Insufficient warning to respond	
Volume based promotions restrictions			Difficult to implement e.g., differences in affected categories	
			Packaged products not able to go at the front of store based on conditions for restrictions (packaged/HFSS/product in	

For peer review only - http://bmjopen.bmj.com/site/about/guidelines.xhtml

Workshop 1 (non-industry)	Workshop 2 (non-industry)	Workshop 3 (non-industry)	Workshop 4 (industry)	Final map
			scope) - may relate to available nutritional info)	
Legal precedent	Influences implementation of other interventions	Influences capacity to make future policies for HFSS		**Regulatory precedent**
Decision norming (more regs seem possible)		Confusion for future policies for HFSS/loss of objectivity		
Empower policymakers		Increase in government sanctioned digital verification		
		Reduced risk of various NCDs		**Risk of diet-related NCDs**
Fewer social connotations around food	Change in social norms around food	Influences social norms e.g., social cooling		**Social norms around food**
Changes in social norms	Family dynamics, individual dynamics	Reduction in normalisation of HFSS		
Change in engagement with foods		Limits changes to food norms		
Associations with young people change (e.g., aspirational)		Greater influence on social/food norms		
				**Society**
Changes link between food and personal identity	Shift towards food citizenship mindset			**Societal shifts**
Different retail environment	Reduce consumerism mindset			
Less time spent on social media	Change in culture			
Polarisation				
Small producers meeting nutritional criteria/ambient food increase sales	Accessibility influences purchases	Increase in advertising from SMEs? If they can afford to compete	Companies with small portfolio of HFSS products most affected? e.g., chocolate	**Changes vary by…**
Different price sensitivity	Increase in advertising among small companies (not franchises)	Sustained sales of HFSS from SMEs	Those already experiencing overweight may be more likely to benefit (higher base exposure?)	
Importance of brand amplified for low-income people		Effects differ by age?	Disproportionately affects low-income people	

For peer review only - http://bmjopen.bmj.com/site/about/guidelines.xhtml

Workshop 1 (non-industry)	Workshop 2 (non-industry)	Workshop 3 (non-industry)	Workshop 4 (industry)	Final map
Importance of brand amplified for young people		Socioeconomic position		
Different groups more susceptible e.g., those exposed		Adolescents more susceptible to advertising		
Differential effects by socioeconomic position				
Baseline differences in health (socioeconomic gradient				
Reduce health inequalities				
Distinguish effects by brand strength e.g., better for established brands				

Notes: HFSS: high fat, salt and sugar; NCD: non-communicable disease; NPD: new product development; NPM: Nutrient Profile Model; SME: small and medium enterprises.

For peer review only
